# Decoding the Self: Single‐Trial Prediction of Self‐Boundary Meditation States From Magnetoencephalography Recordings

**DOI:** 10.1002/hbm.70440

**Published:** 2025-12-26

**Authors:** Henrik Röhr, Daniel A. Atad, Fynn‐Mathis Trautwein, Pedro A. M. Mediano, Yair Dor‐Ziderman, Yoav Schweitzer, Aviva Berkovich‐Ohana, Stefan Schmidt, Marieke K. van Vugt

**Affiliations:** ^1^ Bernoulli Institute for Mathematics, Computer Science and Artificial Intelligence, University of Groningen Groningen the Netherlands; ^2^ Institute for Psychosomatic Medicine and Psychotherapy, University Medical Center Freiburg Freiburg Germany; ^3^ Edmond Safra Brain Research Center, Faculty of Education University of Haifa Haifa Israel; ^4^ The Integrated Brain and Behavior Research Center (IBBRC) University of Haifa Haifa Israel; ^5^ Faculty of Education, School of Therapy, Counseling and Human Development University of Haifa Haifa Israel; ^6^ Department of Computing Imperial College London London UK; ^7^ Division of Psychology and Language Sciences University College London London UK

## Abstract

The sense of self is a multidimensional feature of human experience. Different dimensions of self‐experience can change drastically during altered states of consciousness induced through meditation or psychedelic drugs, as well as in a variety of mental disorders. Some experienced meditation practitioners are able to modulate their sense of self deliberately, which allows for a direct comparison between an active and suspended sense of self. Meditation therefore has the potential to serve as a model‐system for alterations in the sense of self. The current study aims to identify a neural marker of such meditation‐induced alterations in the sense of self based on magnetoencephalography (MEG) recordings of meditation practitioners (*N* = 41). Participants alternated between a state of reduced sense of self, termed self‐boundary dissolution, a resting state and a control meditation state of maintaining their sense of self. Machine learning methods were used to find multivariate patterns of brain activity which distinguish these states on a single‐trial basis. Source band power and Lempel‐Ziv complexity features allowed to predict the mental state from MEG recordings with significantly above‐chance accuracy (> 0.5). The highest performance was obtained for the self‐boundary dissolution versus rest classification based on Lempel‐Ziv complexity, which showed an average accuracy of ~0.64 when training and testing were performed on data from the same individual (within‐participant prediction) and ~0.57 when models trained on one group of individuals were tested on different participants (across‐participant prediction). Potential applications include decoded neurofeedback, for example, for clinical treatments of disorders of the sense of self, or for assistance in meditation training.

## Graphical abstract

Meditation‐induced self‐boundary dissolution can be predicted from magnetoencephalography recordings using Lempel‐Ziv complexity and oscillatory power as neural markers. We demonstrate significantly above‐chance classification of altered self‐experience on a single‐trial basis and show that it is mediated by complex activation patterns across brain regions.

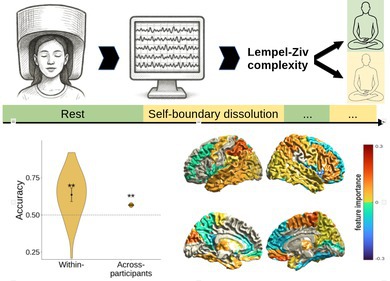

## 1 Introduction

A central feature of consciousness is the sense of self. This has been conceptualized in various ways, including notions such as the minimal self (Gallagher 2000), the pre‐reflective self (Legrand 2006), minimal phenomenal selfhood (Blanke and Metzinger 2009), the core self (Damasio 1999), and the embodied self (Varela et al. 1991). These frameworks specify the self through features such as the sense of self‐location, first‐person perspective, and agency. Despite subtle theoretical differences, these approaches overlap in demarcating basic aspects of self‐consciousness from more conceptual aspects of selfhood, such as autobiographical memory, self‐concept, and higher‐order cognitive processes. The present work aims to shed light on the malleability of this basic embodied sense of self, which can undergo profound changes during meditation or psychedelic states (Millière et al. 2018) and in certain mental disorders including schizophrenia (Sass and Parnas 2003; Sass 2013), depersonalization (Sierra and David 2011; Gerrans 2019), and dissociation (Whitmer 2001). We thus focus on the concept of the embodied self, which emphasizes how selfhood is shaped through continuous interaction with the environment and underscores its malleable and dynamic nature. This highlights that the self is not a fixed entity but rather a fluid construct that evolves in response to contextual and bodily changes (Varela et al. 1991; Kyselo 2014, Newen 2018).

A key scientific challenge is to identify neural markers of the embodied self that would allow states of altered selfhood to be tracked objectively. In the present study, we address this challenge by analyzing neurophysiological data from long‐term meditation practitioners, a population in which past studies reported reliable and deliberate modulations of the sense of self (Berkovich‐Ohana et al. 2020; Nave et al. 2021). Meditation therefore holds the potential to serve as a model for examining states of altered selfhood in a controlled setting.

The ability to characterize neural signatures of modulations of the embodied self has both scientific and translational implications. Scientifically, it advances our understanding of how neural dynamics relate to the phenomenology of the embodied self and its modulations. Moreover, it has been suggested that there may be structural commonalities between meditation‐induced alterations of selfhood and pathological self‐disturbances (Deane et al. 2020; Kirberg and Chadha 2024). Although self‐boundary dissolution in meditation differs markedly from psychopathological states in valence and degree of voluntary control, they also share phenomenological dimensions such as changes in ownership, agency, and first‐person perspective. Meditation might therefore provide a tractable, controlled, and ethically accessible window into self‐boundary modulation which could suggest candidate biomarkers or mechanistic hypotheses for self‐disorders. Translationally, such markers could inform the development of neurofeedback‐based interventions for disorders of the self or as a support for meditation training. Such neurofeedback approaches depend critically on reliable neural markers, making robust signatures of self‐modulation essential for their implementation. Recent work of decoding meditative depth from electroencephalography (EEG; Reggente et al. 2025) demonstrated that machine learning methods captured neural dynamics that conventional channel‐ and source‐level analyses failed to detect, highlighting the superior sensitivity of multivariate machine‐learning approaches over conventional EEG analyses. Building on prior work identifying neural markers of self‐boundary dissolution (Atad et al. 2025a; Dor‐Ziderman et al. 2016; Trautwein et al. 2024), we apply machine learning methods to capture multivariate neural patterns, a refinement essential both for advancing scientific understanding and for progressing toward translational applications.

While the dissolution of self‐boundaries has received wide interest in the cognitive and contemplative sciences (Kitson et al. 2020), this exploration is rooted in a long tradition of contemplative practices in which the investigation and transcendence of the sense of self has been described extensively. This is especially pronounced in Buddhism from which the modern practice of mindfulness is derived (Kabat‐Zinn 1990; Wahbeh et al. 2018). These changes can occur either as a byproduct of different meditation practices or through an intentional dissolution of the sense of self (e.g., Hanley et al. 2020; Yang et al. 2024; Ataria 2015). The phenomenological character of such changes has been investigated in a series of studies by Berkovich‐Ohana and colleagues (summarized in Berkovich‐Ohana et al. 2020). Their work has shown that such alterations can involve a profound modulation and dissolution of implicit distinctions of self and world. This was described as a sense of unity between self and world, as well as a weakening or absence of ownership of the body and distinct bodily experiences (Ataria 2015). This self‐world distinction has been termed ‘sense of boundaries’ (or self‐boundaries), and the dissolution of it as self‐boundary dissolution. Self‐boundaries are described as a construct which involves a dynamic distinction between oneself and the environment in which this self is immersed. This is not limited to the sense of the physical body and its sensations, but also includes spatial, perspectival, agentic and attentional features (Ataria et al. 2015; Nave et al. 2021). Thus, in boundary dissolution, the sense of self‐location and perspective becomes blurred or vanishes, agency is suspended and attention is open or even entirely formless. In other words, boundary dissolution describes a process where structures that delineate subject and object are increasingly lost.

On a neural level, changes in self‐boundaries have been investigated in two consecutive studies with experienced meditators in the Buddhist practice of Vipassana meditation (Dor‐Ziderman et al. 2016 with *N* = 1 participant and validation in another dataset of *N* = 10 participants; Trautwein et al. 2024 with an independent group of *N* = 46 participants). In both studies, MEG was recorded while participants entered a state of self‐boundary dissolution or a control state in which the boundary was maintained, and the degree of their dissolution was assessed through phenomenological interviews. Both studies found that a dissolution of self‐boundaries correlated with a reduction in oscillatory power in the beta band, with a peak at 27 Hz. These effects were source‐localized to posterior medial, frontoparietal and lateral parietal cortices. Besides oscillatory power, self‐boundary dissolution has also been found to be associated with an increase in temporal signal complexity (Atad et al. 2025a). Temporal signal complexity can be quantified as Lempel‐Ziv complexity (LZc), which measures the diversity of a signal by counting its unique patterns (Lempel and Ziv 1976). Based on MEG recordings of Vipassana meditators the same data used by Trautwein et al. (2024), Atad et al. (2025b) found robust and widespread increases in broadband LZc (alongside local reductions in the orbitofrontal cortex) in self‐boundary dissolution compared to rest. This is in line with the more general observation that temporal complexity of the signal time course is a reliable marker of different states of consciousness (reviewed in Sarasso et al. 2021). Complexity has therefore been suggested to index level of consciousness (with reductions in anaesthesia, sleep, etc., e.g., Casali et al. 2013) and altered states of consciousness (with increases in psychedelic, e.g., Mediano et al. 2024, and meditative states, reviewed in Atad et al. 2025b).

While neural markers of self‐boundary dissolution were found both in the frequency domain and in temporal complexity, these results were all based on univariate comparisons, that is, variables such as sensors, voxels or brain regions were compared individually. However, considering that self‐boundaries are a multidimensional construct which involves many different aspects of experience, it seems highly unlikely that their neural correlate is restrained to the brain regions found by Trautwein et al. (2024) and Atad et al. (2025b). Instead, self‐boundaries are likely mediated by complex patterns of neural activity across many different brain regions. Univariate analyses are unable to detect these multivariate patterns and therefore only use part of the available information. To increase the sensitivity of the objective marker of self‐boundary dissolution, we applied machine learning methods which allow us to detect patterns of information across different brain regions. Compared to relying on individual brain regions, this has the advantage of accumulating weak information from several locations and allows us to detect information which is contained in the patterns of several variables rather than being carried by a single variable (Tong and Pratte 2012). This technique has been applied to a range of single‐trial classification tasks in the areas of brain–computer interfacing and real‐time monitoring of mental states (reviewed in Müller et al. 2008).

Based on magneto‐ and electroencephalography (M/EEG) recordings, several studies have demonstrated the feasibility of using machine learning methods to distinguish meditation from non‐meditation and from other meditation states (e.g., Zhigalov et al. 2019 for MEG; see Kora et al. 2021 and Lin et al. 2025 for a review of EEG‐based studies). We therefore seek to demonstrate single‐trial prediction of self‐boundary dissolution against both a resting and a control meditation state. To this end, we examine two classification tasks: a within‐participant task, where training and testing data come from the same individual, and an across‐participant task, where the classifier is trained on one group of participants and tested on another. The within‐participant approach demonstrates the potential for applications in which training data can be collected from the same patient prior to an intervention, while the across‐participant approach supports broader use cases without prior data collection and offers insights into generalizable neural patterns underlying self‐boundary dissolution. Beyond insights into neural mechanisms underpinning the sense of self, the detection of self‐boundary states based on a neural marker promises practical applications, for example for decoded neurofeedback. Neurofeedback has been used in the past as a training tool to assist participants in modulating certain aspects of their neural activity (see Sitaram et al. 2017 for review), which might be useful as support for meditation training (Brandmeyer and Delorme 2013; Chen and Ziegler 2025). Moreover, if meditation is indeed a model‐system for states of altered selfhood in other contexts such as mental disorders of the self, our results might lay the foundation for EEG‐based biomarkers as well as potential new treatment approaches for such disorders.

## 2 Methods

### 2.1 Data Collection

#### 2.1.1 Participants

We used an existing dataset of MEG recordings which had been collected by Trautwein et al. (2024). The data set consists of recordings of 46 meditation practitioners (aged 26–72, mean age = 39.8 ± 10.9, 27 males and 19 females) with a wide variance of meditation experience (115–24,837 h, mean = 3832 ± 4845 h). Five of the recordings were incomplete due to technical issues, resulting in partial data that did not allow for comparable classifier training and were therefore not included in our analysis. This left us with *N* = 41 complete datasets. Participants were recruited mainly through Tovana (The Israel Insight Meditation Society). They were selected to represent a wide variety in expertise levels to allow the detection of expertise‐dependent effects. Inclusion criteria were the attendance of at least one meditation retreat (1 week or more) and a minimum of 1 year of practice. Exclusion criteria were active psychiatric disorders, current psychiatric medication, conditions that limit MEG data quality (artificial cardiac pacemakers, dental splints), and not having normal or corrected‐to‐normal vision or hearing. All participants had participated in a 3‐week meditation training program with a special focus on self‐boundaries in preparation for the laboratory sessions. For additional details on the training program, see Trautwein et al. (2024). The study was approved by the Institutional Review Board of the Faculty of Education, University of Haifa, Israel. Participants received the training free of charge and compensation for their travel expenses to the laboratory.

#### 2.1.2 MEG Recordings

MEG recordings were conducted using a whole‐head 248‐channel magnetometer array (4‐D Neuroimaging, Magnes 3600 WH) with a sampling rate of 1017.25 Hz and an online 1–400 Hz band‐pass filter in a magnetically shielded room. Environmental noise was removed by reference coils, and head shape and coil position were digitized. For additional details on the experimental setup, see Trautwein et al. (2023).

#### 2.1.3 Procedure

While being MEG recorded, participants were asked to alternate between a non‐meditative resting state in which they let their minds wander freely, a state of self‐boundary dissolution (SB−) in which they dissolved their sense of self‐boundary, and a state of self‐boundary maintenance in which they maintained a clear sense of their self‐boundaries (SB+). Self‐boundary maintenance involved a focused, embodied presence, where attention was actively directed toward localized bodily sensations, strengthening a clear sense of where the self ends and the world begins. In contrast, self‐boundary dissolution was described as a state of expanded or indeterminate self‐location, indistinct or nonlocal bodily sensations, as well as a passive sense of agency (non‐doing) and formless attention, which was often induced by a mental gesture of “letting go.” For a more detailed description of the phenomenology of SB− and SB+, see Nave et al. (2021).

**FIGURE 1:**
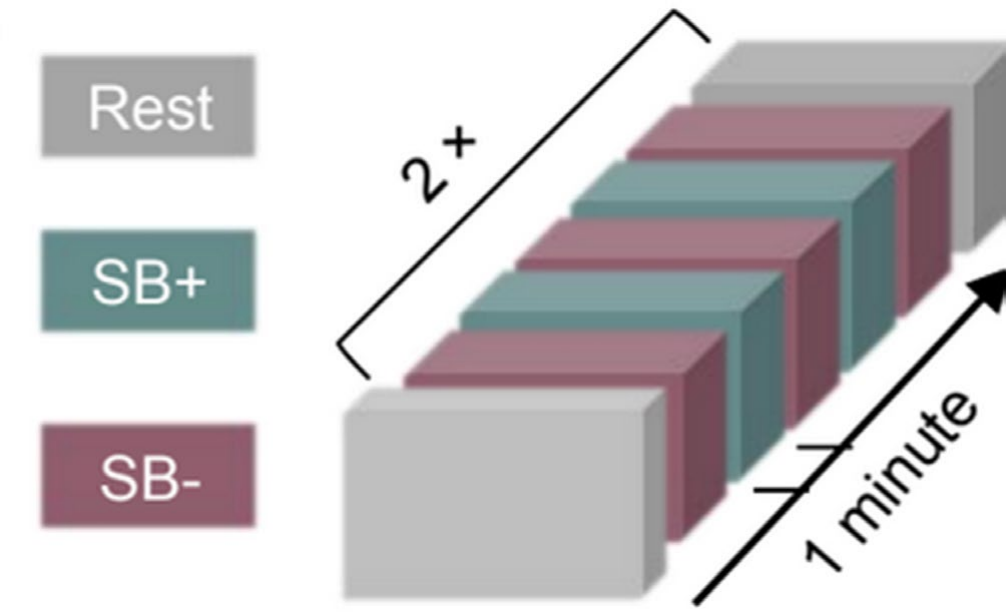
Representation of procedure of mental states during MEG recording. SB+: Self‐boundary maintenance; SB−: Self‐boundary dissolution. Reproduced with permission from Trautwein et al. (2024).

The procedure involved two blocks, each of which started with a resting state (100 s), followed by five alterations between SB− and SB+ (60 s each). A third resting state measure was added at the end. The entire recording amounted to a total of 300 s of MEG data for each state per participant. See Figure 1 for a visual representation of the procedure.

### 2.2 Data Preparation

#### 2.2.1 Preprocessing

The MEG data were preprocessed and cleaned as detailed in Trautwein et al. (2024) by using the FieldTrip toolbox (Oostenveld et al. 2011). This involved the removal of artifacts caused by heartbeats, which were identified via electrocardiogram (ECG)‐derived templates averaged across MEG channels. Artifactual cardiac events were then subtracted from the MEG data using these averaged templates (as described in Tal and Abeles 2013). Additionally, power line frequency was corrected via recording on an extra channel, and building vibrations were corrected via recordings from accelerometers on the MEG gantry. Moreover, two sensors showed excessive noise across recording sessions and were therefore removed from all recordings. Movement, ocular and heartbeat‐related artifacts were removed by visual inspection and Independent Component Analysis, which involved downsampling the data to 300 Hz, decomposition via the runica algorithm with all components, and unmixing and reconstruction of the original data without artifactual components.

#### 2.2.2 Source Band Power

Source band power was localized by using an adaptive spatial filtering beamformer (Gross et al. 2001). This method was chosen because it directly localizes oscillatory activity and therefore does not require an additional transformation to the frequency spectrum, and also to allow comparability to the previous univariate pipeline (Trautwein et al. 2024). The forward model used a semi‐realistic head model of volume conductance (Nolte 2003). This model was constructed for each participant based on a single‐subject MRI template that was fitted to the participant's digitized head shape. This head model was overlaid with a three‐dimensional source grid (1 cm resolution) and a lead field.

A lead field matrix was computed for each grid position based on the head position in the MEG system and the volume conduction model. Data were segmented into 1‐s epochs, which are long enough epoch lengths to contain a full oscillation of the lower frequency cutoff at 1 Hz while still allowing for the largest possible number of data points. The epoched data were transformed from the time to the frequency domain by using Fast Fourier Transform (FFT) with a Hanning taper at 1 Hz resolution, from which the cross‐spectral density matrix was computed. A common spatial filter was constructed for each grid point based on the cross‐spectral density matrices and lead field for the entire frequency spectrum (1–90 Hz), and single trial source power was computed for each grid point and each frequency to yield a source‐level power spectral density (1 Hz resolution).

Recent work has shown that the power spectrum contains not only periodic (oscillatory) components but also an aperiodic component, characterized by the 1/*f* property. The aperiodic component carries meaningful information but is often conflated with oscillatory power in standard analyses (Donoghue, Dominguez, and Voytek 2020; Gerster et al. 2022). We therefore computed both the periodic and aperiodic components of the source‐localized power spectral density, as well as the aperiodic 1/*f* slope via the implementation of the FOOOF method (Donoghue, Haller, et al. 2020) in Fieldtrip, and subtracted the aperiodic contribution from the periodic component to obtain the “corrected” source spectral power. After correcting for the aperiodic component, oscillatory power was averaged over all bins within a frequency band. The frequency bands were defined as in Trautwein et al. (2023) to allow for a comparison with their univariate analysis, see Table S1, for the precise definitions. Besides the corrected oscillatory power, we also used the aperiodic component as an additional feature by extracting the aperiodic slope of the 1/*f* component, represented by the exponent of the 1/*f* background activity.

#### 2.2.3 LZc of Source Time Course

LZc measures the complexity of a time course by counting its unique patterns. Considering that the adaptive spatial filtering beamformer used in the spectral power analysis does not yield a source time course over which LZc can be computed, sources were instead reconstructed with a linearly constrained minimum‐variance (LCMV) beamformer (Van Veen and Buckley 1988). The head model and lead field were created as described in the previous section, and the covariance matrix was derived from the sensor data of the entire recording. Time series at each source location were computed for each grid point based on the lead field and a multiplication of the covariance matrix with the sensor time series. To calculate LZc, the source time course was band‐pass filtered at 1–100 Hz and concatenated into 4 s epochs, which we found to be the minimum epoch length for which LZc could be reliably estimated. The data were detrended and binarized using the mean of all samples within an epoch as a threshold. For each binary vector, LZc was computed using the LZ76 algorithm (Lempel and Ziv 1976; Kaspar and Schuster 1987), which works by counting the number of distinct substrings in the binary vector. LZc is thus minimal if the vector only repeats the same value, and increases with a more diverse vector. The resulting LZc values in each epoch in each grid point were normalized by dividing the computed value by *T*/log_2_(*T*), where *T* is the vector length. This normalization ensures that a fully predictable signal is assigned a complexity value of 0, and a fully random (i.e., unpredictable) signal is assigned a value of 1.

#### 2.2.4 Parcellation

The source‐localized data was based on 1457 voxels inside the brain. Compared to the number of observations (approximately 600 for within‐participant classification), this constituted an excessively large number of features and therefore would have increased the likelihood of overfitting (Hall and Holmes 2003). To avoid such a high‐dimensional feature vector, we parcellated the data based on a modified version of the automated anatomical labeling (AAL) atlas (Tzourio‐Mazoyer et al. 2002). Compared to the original AAL atlas, small regions were merged (as in PMOD Technologies LLC 2022) and all regions belonging to the Cerebellum and Vermis were excluded. Merged regions are listed in the Supporting Information Materials. This resulted in a total of 62 regions. Each voxel inside the brain was assigned to its corresponding brain region, and an overall value for each region was computed by taking the mean over all voxel values in that region.

### 2.3 Univariate Analysis

Prior to the application of machine learning methods, we conducted a region‐wise comparison between the states of interest. This pre‐step should check the general feasibility of finding a univariate difference between these states and serve as a benchmark against which the multivariate analysis could be compared. For each feature, the effect size of these differences was assessed in two ways:

First, the accuracy of a univariate prediction based on individual brain regions was computed to allow for a direct comparison with multivariate methods. For each brain region, the mean was taken across all observations in both classes (SB− was compared separately to rest and SB+). As a working hypothesis, we assumed that observations from the SB− class would show oscillatory power below and LZc above this mean, while observations from the rest or SB+ class would fall on the other side of the mean. The accuracy of this prediction was assessed by comparing it to the actual values. This was done for within‐participant data, for which the resulting accuracy values were averaged across participants and for across‐participant data.

Second, effect size between classes was assessed by Cohen's *d* because this is a standardized, widely used, and easily interpretable measure. This was done by accumulating the data across all participants and computing Cohen's *d* in each brain region for the contrasts SB− versus rest and SB− versus SB+.

### 2.4 Classification

Each epoch (1 s for band power and 4 s for LZc) was considered as one observation and consisted of a 62‐dimensional feature vector, where each feature was one of the regions of the modified AAL atlas. The classification task was binary classification, that is, we trained two separate classifiers to distinguish between two classes (SB− vs. rest or SB− vs. SB+). Binary classification was chosen over a single model with three classes because we were interested in how well SB− could be predicted from each alternative state in isolation.

Standard scaling was applied to the data to transform them such that they had a mean of 0 and standard deviation of 1 to improve classification performance (Ahsan et al. 2021). A total of 80% of the data were used as a training set and to tune hyper‐parameters, while the remaining 20% of the data were used as a hold‐out set for evaluation, which is a common split that has been shown to be optimal in a wide range of contexts with respect to bias and variance in error estimation. For across‐participant classification, the data were split such that data from the same participant were never divided between sets, and participants were assigned randomly to training and evaluation sets. For within‐participant classification, the data were split in half, and from each half the first 80% were used as training (TS) and the last 20% as evaluation set (ES; i.e., first 40% TS, 40%–50% ES, 50%–90% TS, 90%–100% ES). This chronological split was applied to preserve continuity of the data, thereby avoiding potential data leakage due to allocating neighboring epochs into training and evaluation set. The split was applied separately to the first and second half of the data to reduce the influence of potential drifts over time. Additionally, one segment at the intersection between training and evaluation set was removed to avoid data leakage between neighboring epochs.

Minor imbalances in class size between categories existed due to epochs which were removed as artifacts (< 2% difference in class size). To re‐balance classes, the minority class was over‐sampled with the SMOTE algorithm (Chawla et al. 2002). This was done separately for the training and evaluation set to rule out that information from the training set might be used to generate data points which ended up in the evaluation set.

Training and hyper‐parameter tuning were conducted by using grid‐search with fivefold stratified cross‐validation on the training set. Model performance was then evaluated on the evaluation set. Performance was assessed by the classification accuracy, which in the case of balanced classes has the advantage of allowing for a straightforward interpretation of results.

The primary classification method used was logistic regression (LR) with scikit‐learn's liblinear solver and L1 regularization (Pedregosa et al. 2011). This was chosen due to its simplicity and interpretability. The hyper‐parameter “C” (regularization) was selected through hyper‐parameter tuning by using grid search with fivefold stratified cross‐validation and refitting the model on the hyper‐parameters with the highest accuracy.

To compare the performance of different classification methods, the following classification methods were tested additionally:

- Support Vector Classifier with a Radial Basis Function kernel (SVC‐RBF), with hyper‐parameters “C” (regularization) and “gamma” (kernel coefficient).
- Random Forests (RF) with the hyper‐parameters “number of trees in forest,” “number of levels in decision tree” and “minimum number of data points in node before node is split.”
- Gaussian Naive Bayes (GNB), for which no hyper‐parameters were tuned.

The reason for selecting these classifiers was that they all are easy to interpret and have been used in similar classification tasks (e.g., Halme and Parkkonen 2016; Jin et al. 2019; Kaushik et al. 2022; Carota et al. 2022; Huttunen et al. 2013). We decided to use classical machine learning rather than deep learning as there was not sufficient data for deep learning. All classifiers were applied through their implementation in the Scikit‐learn package (Pedregosa et al. 2011).

### 2.5 Statistical Evaluation of Classification Results

The results of each binary classification task were compared against chance level (0.5 accuracy) by using a two‐sided binomial test. To summarize the binomial tests for within‐participant classification across the individual classification tasks of each of the 41 participants, the test results were combined by using Fisher's method for combining *p* values. This method combines independent *p* values by summing their negative logarithms, producing a *χ* ^2^ statistic that tests overall significance (Fisher 1992). This yielded a single *p* value for all the within‐participant classification results. In our entire analysis we conducted a total of 44 binomial tests. False discovery rate correction (Benjamini and Hochberg 1995) was applied to correct for multiple comparisons. This correction was applied separately to the univariate and multivariate tests because they were considered as two distinct sets of hypotheses. A corrected *p* value below 0.05 was considered significant.

### 2.6 Importance of Components

To better understand what the classifiers had learned, we assessed the importance of each individual feature, that is, each brain region, for the logistic regression classification. Feature importance was quantified by the regression coefficients, which are one of the most easily interpretable and commonly used feature importance measures for logistic regression models with L1 regularization (Saarela and Jauhiainen 2021). Although coefficient‐based feature‐importance estimates can be sensitive to multicollinearity among predictors, L1 regularization penalizes redundant predictors and thereby stabilizes regression coefficients despite inter‐regional correlations, which are shown in the Supplementary materials, section 7. As standard scaling has been applied to the input data, these coefficients can be interpreted as the importance of their respective features because features with larger absolute coefficients have a greater impact on the prediction.

### 2.7 Correlation With Lifetime Meditation Hours and Phenomenology

To better understand which factors the classification performance depended on, we tested whether the result of the logistic regression was correlated with the number of lifetime meditation hours and participants' depth of self‐boundary dissolution, assessed by an index derived from micro‐phenomenological interviews (see Trautwein et al. 2024; Nave et al. 2021 for details regarding this index). Two measures of logistic regression results were tested: first, the within‐participant logistic regression classification accuracy, and second, the average logit linear predictors of the within‐participant logistic regression. The accuracy or logit values were averaged over all observations of a participant to get a single value for each participant, based on which the correlations were calculated. Outliers were removed using the interquartile range (IQR) method. In addition to assessing correlation with the Spearman correlation coefficient, we used Jeffreys's default Bayes factor (Ly et al. 2016) to evaluate both the evidence for and against a correlation.

## 3 Results

### 3.1 Multivariate Equals or Outperforms Univariate Classification

**FIGURE 2:**
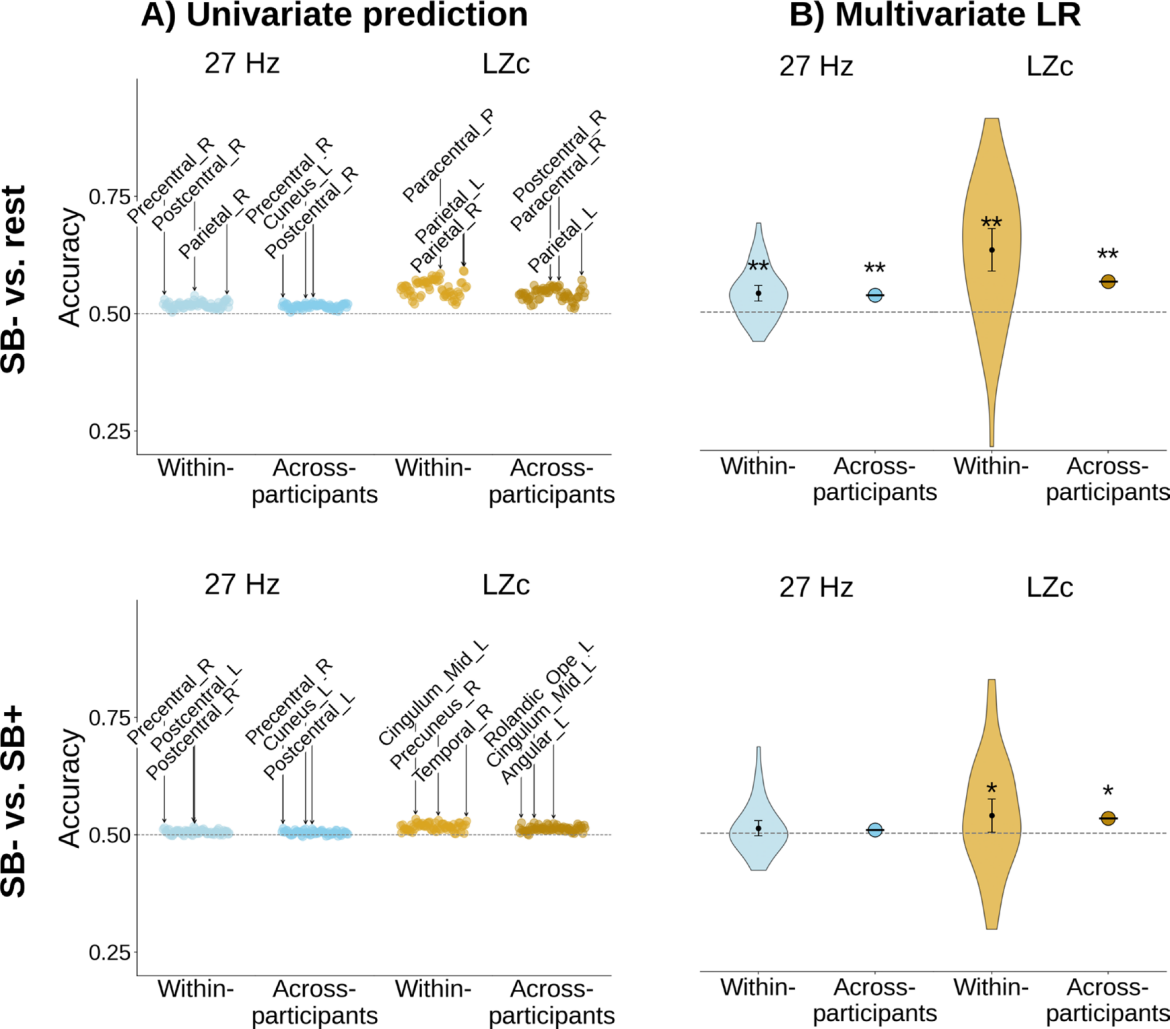
Comparison of predictive accuracy between individual brain regions and logistic regression (LR) for 27 Hz oscillatory power (blue) and LZc (gold). (A) Univariate classification. Each dot represents the prediction accuracy for an individual brain regions, with the three regions of highest accuracy marked. (B) Logistic regression classification. Within‐participant accuracy signifies mean classification accuracy across within‐participant classification attempts. Error bars indicate 95% confidence intervals. *Significantly above chance, FDR‐corrected *p* < 0.05. **FDR‐corrected *p* < 0.001. Dashed line indicates chance level (50%).

We used a multivariate logistic regression model to predict the brain states during self‐boundary dissolution (SB−), rest, and self‐boundary maintenance (SB+). To evaluate whether this multivariate model outperformed a univariate prediction, an additional region‐wise comparison was conducted between these brain states. These analyses were based on two brain markers: First, oscillatory source power around the 27 Hz peak frequency in which Dor‐Ziderman et al. (2016) and Trautwein et al. (2024) found the strongest distinction between the mental states. Second, LZc, a measure which quantifies temporal complexity, which was found to be a marker for SB− by Atad et al. (2025b). The univariate and multivariate predictive accuracies are shown in Figure 2, as well as numerically in Table 1. In addition, the effect size of region‐wise differences was assessed by Cohen's *d*, which is shown in Figure 5A and the Supporting Information Materials (Figure 1).

**TABLE 1:** Numerical results of the univariate effect size measures and the multivariate logistic regression (LR).

			Univariate			Multivariate LR	
			Cohen's *d*	Accuracy	Binom. test	Accuracy	Binom. test
27 Hz	Within‐participant	SB− vs. rest		0.539	*p* < 10^−10^	0.541 ± 0.015	*p* < 10^−6^
		SB− vs. SB+		0.519	*p* < 10^−5^	0.507 ± 0.018	*p* = 0.060
	Across‐participants	SB− vs. rest	0.1–0.2 (very small)	0.526	*p* < 10^−10^	0.536	*p* < 10^−6^
		SB− vs. SB+	< 0.1 (negligible)	0.514	*p* < 10^−3^	0.507	*p* = 0.154
LZc	Within‐participant	SB− vs. rest		0.593	*p* < 10^−10^	0.635 ± 0.046	*p* < 10^−10^
		SB− vs. SB+		0.534	*p* < 10^−3^	0.538 ± 0.036	*p* < 0.05
	Across‐participants	SB− vs. rest	0.2–0.4 (small to medium)	0.572	*p* < 10^−10^	0.566	*p* < 10^−5^
		SB− vs. SB+	0.1–0.2 (very small)	0.527	*p* < 10^−3^	0.532	*p* < 0.05

*Note:* The binomial test compared the classification accuracy against chance level (50%). It should be noted that similar accuracies might have different *p* values because 4‐s epochs were used for LZc and 1‐s epochs for 27 Hz, which yielded more observations for the latter (300 observations for 27 Hz and 75 observations for LZc per class for each participant). Moreover, FDR correction was applied separately for the univariate and multivariate comparisons.

The univariate results can be summarized as follows: First, the predictive accuracy of the best performing individual brain regions was significant above chance level for all classification tasks (27 Hz and LZc, contrasts SB− vs. rest and vs. SB+, within‐ and across‐participant classification). The best results were achieved for SB− versus rest in the within‐participant classification, with accuracies of up to 0.54 for 27 Hz and 0.59 for LZc. Note that the decision boundary for the univariate classification was defined as the grand average across conditions. A search over a range of decision boundaries demonstrated that in most cases the grand average was very close to the optimal decision boundary, with only minor improvements for alternative boundaries (max. 0.007). Second, effect sizes were considerably larger for the contrast SB− versus rest than for SB− versus SB+, and third, effect sizes were considerably larger for LZc (0.2–0.4, i.e., small to medium in SB− vs. rest) than for 27 Hz (0.1–0.2, i.e., very small in SB− vs. rest). With respect to the effects of individual regions, the parietal regions showed the strongest decrease in 27 Hz as well as most other frequency bands. For LZc, the effect was also localized in the parietal regions, but was spread more widely. These regions and direction of change between SB− and the control conditions are consistent with the findings by Trautwein et al. 2024 and Atad et al. (2025a).

While the univariate analysis considered only individual brain regions, multivariate logistic regression allowed us to determine how a combination across brain regions could predict the state of self‐boundaries. This analysis showed the following results: First, multivariate logistic regression showed a substantial improvement over univariate prediction in the within‐participant classification task (with the highest accuracy of 0.64 for LZc in the SB− vs. rest contrast), but performed nearly identically in the across‐participants task. Second, results were significantly above chance for all tasks (27 Hz and LZc, SB− vs. rest and SB− vs. SB+ and within‐ and across participants), except for 27 Hz SB− versus SB+. Third, performance was substantially better for LZc than for 27 Hz, for SB− versus rest than for SB− versus SB+, and also better for within‐participant than for across‐participants classification. Moreover, we found that self‐boundary dissolution is mediated by a combination of regions rather than individual regions, as will be shown below.

We could thereby replicate the findings by Dor‐Ziderman et al. (2016), Trautwein et al. (2023), and Atad et al. (2025a) that oscillatory power around 27 Hz and LZc are markers for self‐boundary dissolution, and show that the multivariate approach can surpass these, especially for within‐subject effects in LZc.

### 3.2 Predictions Make Use of Information Which Is Spread Over Different Frequency Bands

**FIGURE 3:**
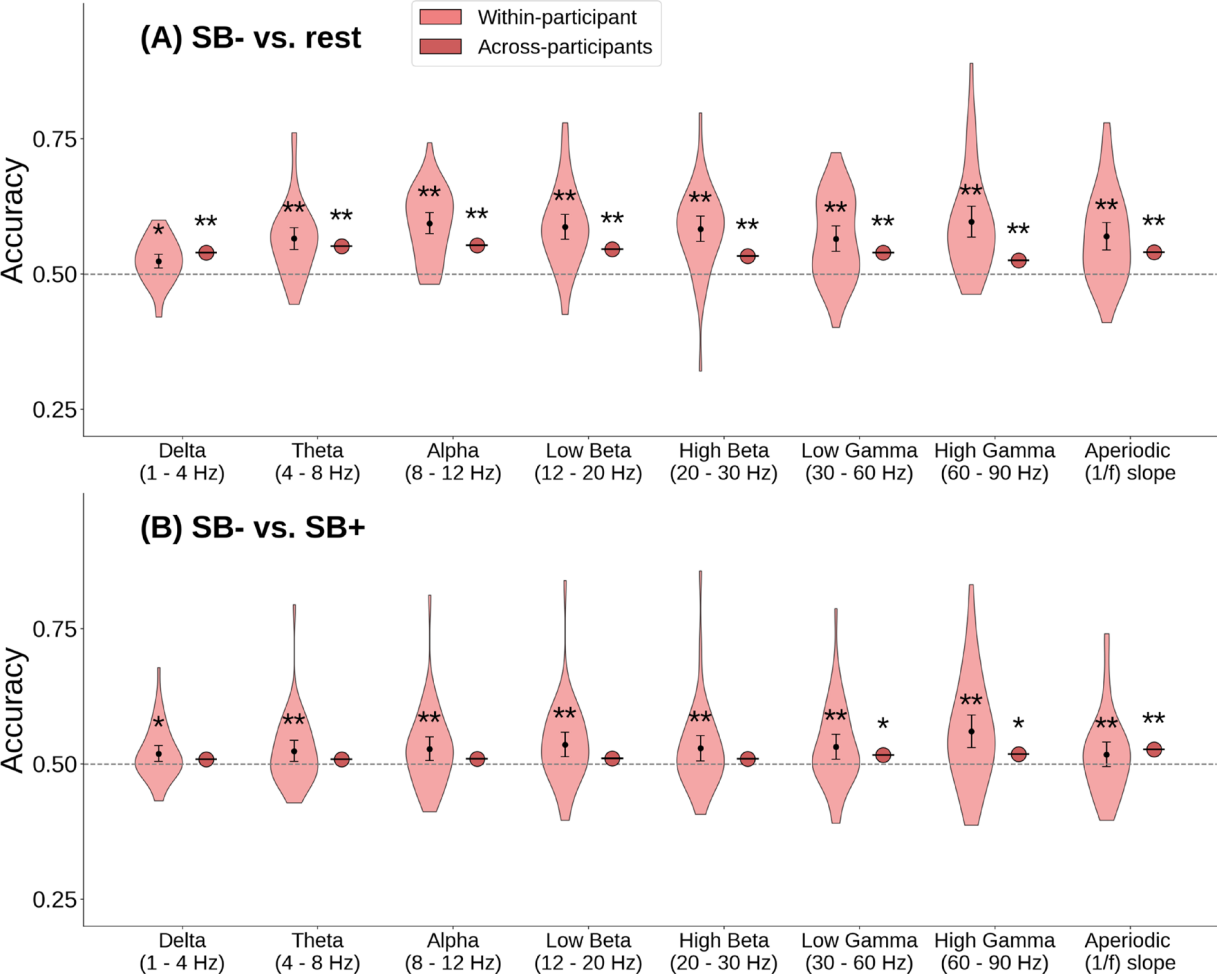
Classification of self‐boundaries on the bases of band power in different frequency bands (corrected by aperiodic component) and aperiodic slope by means of logistic regression. Within‐participant accuracy signifies mean classification accuracy across within‐participant classification attempts. Error bars indicate 95% confidence intervals. Dashed line indicates chance level (50%). *Significantly above chance, FDR‐corrected *p* < 0.05 **FDR‐corrected *p* < 0.001. (A) SB− versus rest and (B) SB− versus SB+.

The previous analyses focused on features of interest defined by previous studies (Dor‐Ziderman et al. 2016; Trautwein et al. 2024; Atad et al. 2025a). Additionally, we aimed to evaluate the predictive power across a broader range of oscillatory frequencies. We therefore tested if SB− could be distinguished from the control states based on oscillatory power in the canonical frequency bands after correcting for the aperiodic component, as well as for the aperiodic (1/*f*) slope. Here, the univariate analysis showed region‐wise differences in all features in SB− versus rest, and for most features in SB− versus SB+ (see Supporting Information Materials, section 2). The multivariate results based on logistic regression for each frequency band are shown in Figure 3.

For SB− versus rest, the within‐participant classification results were significantly above chance (*p* < 0.05 for delta, all other *p* < 10^−5^), with an accuracy range between 0.528 (1–4 Hz delta band) and 0.598 (60–90 Hz high gamma band). The across‐participant classification results for the SB− versus rest contrast were significantly above chance (all *p* < 10^−5^) for all bands, with very similar results in all bands between 0.526 and 0.547.

The contrast SB− versus SB+ showed significant results in the within‐participant classification for all bands (*p* < 0.05 for delta, all other *p* < 10^−4^), with the highest accuracy in the 60–90 Hz high gamma band with 0.562. For the across‐participant classification tasks, only the gamma bands and aperiodic slope showed significant above‐chance accuracies between 0.517 and 0.527.

Compared to the univariate prediction, the accuracies in the logistic regression were higher than the best performing individual brain regions for all within‐participant comparisons except for the 1–4 Hz delta band. Across‐participant accuracies, however, were very similar in the logistic regression as in the best performing individual brain regions of the univariate comparison.

Results for most frequency bands were very similar to those for power around 27 Hz, which suggests that the predictive power is distributed widely across frequency bands. This might also partly explain the higher performance in LZc than in any individual frequency range, because LZc was computed over a broad frequency range (1–100 Hz) and therefore likely accumulates predictive power across different bands. Moreover, the aperiodic (1/*f*) slope had a very similar predictive power to the periodic features, and the uncorrected band power (i.e., without subtraction of the aperiodic component, see Figure S2) showed very similar accuracies to the corrected band power (except for the gamma band where the uncorrected accuracy was substantially higher, possibly due to subtle muscle artifacts). This suggests that for our specific task there was no advantage of decomposing periodic and aperiodic components.

### 3.3 Results Replicate for Different Classification Methods

**FIGURE 4:**
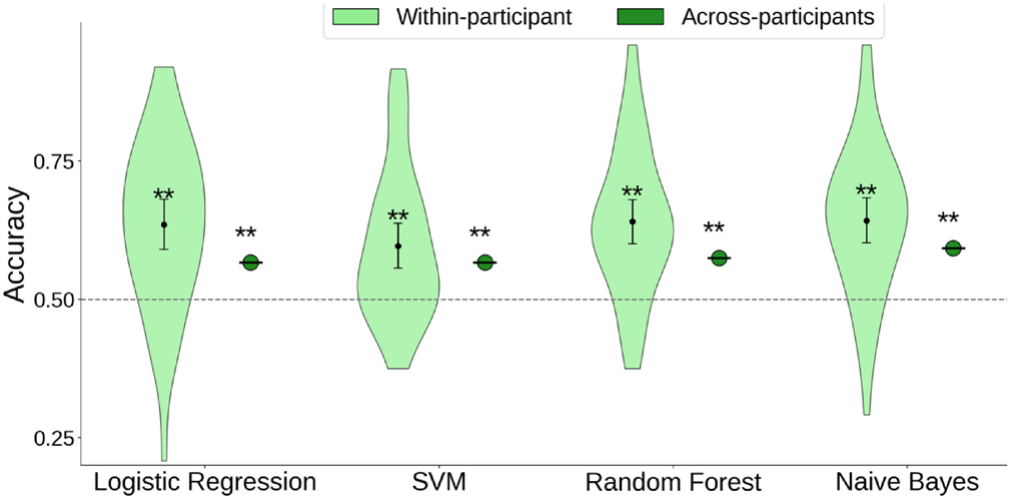
Classification of rest versus SB− on the basis of LZc using different classification methods. Within‐participant accuracy signifies the mean of within‐participant results. Error bars indicate 95% confidence intervals. Dashed line indicates chance (50%). *Significantly above chance, FDR‐corrected *p* < 0.05 **FDR‐corrected *p* < 0.001.

Lastly, we compared the performance of different classification methods to evaluate whether the results would replicate across different methods. This was done based on the LZc feature in the contrast SB− versus rest, since that contrast showed the clearest results. In addition to logistic regression, we used SVM, Random Forests, and Naive Bayes classifiers. The results are shown in Figure 4.

For the within‐participant classification, SVM was less accurate (0.597 accuracy), while the three other methods performed very similarly, ranging from 0.635 for Logistic Regression to 0.643 for Naive Bayes (all significantly above chance according to a binomial test comparing performance to 0.5, *p* < 10^−10^). The across‐participant classification results were similar as well, with a range from 0.566 for SVM and Logistic Regression to 0.593 for Naive Bayes (all significantly above chance, *p* < 10^−5^).

Overall, this indicates that the results replicate well across different classification methods and that only the SVM was slightly less suited for the task while the other methods showed very similar results.

### 3.4 Importance of Components

**FIGURE 5:**
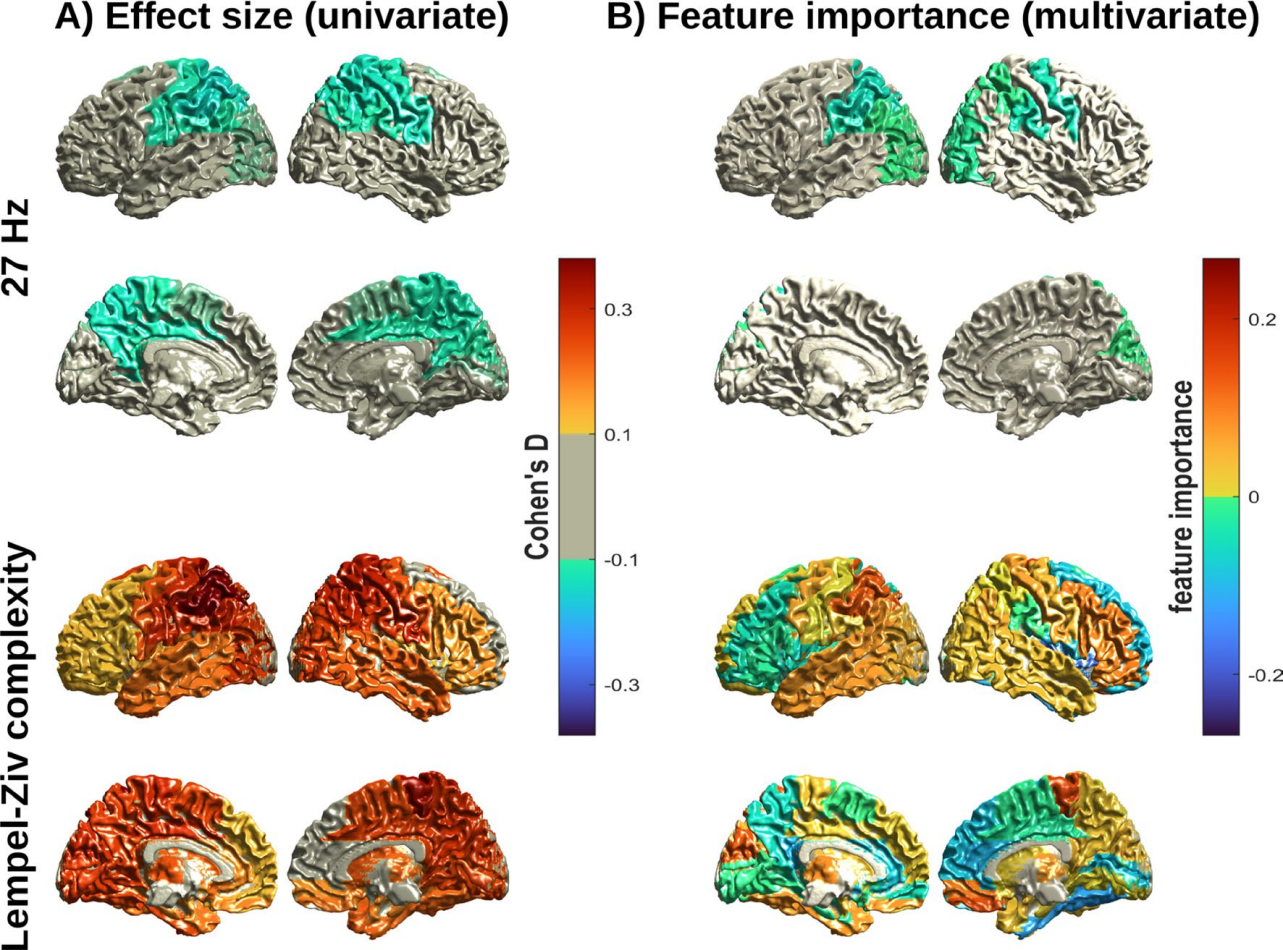
Region‐wise assessment of the contrast SB− versus rest for power around 27 Hz (top) and LZc (bottom). (A) Cohen's *d* effect size across all participants. (B) Feature importance as measured by the logistic regression coefficients. For SB− versus SB+ see Figure S1.

To better understand which characteristics of the data were important for predicting the state of self‐boundaries, we looked at the importance of the components (brain regions) for the classification outcome. This was quantified by using the logistic regression coefficient in each region, which can be interpreted as the importance of this region for the classification task (Saarela and Jauhiainen 2021). Results are shown graphically in Figure 5B for power around 27 Hz and LZc in the SB− versus rest contrast, as well as numerically in Table 2. See Supporting Information Materials, section 3, for SB− versus SB+ and other frequency bands.

**TABLE 2:** Regression coefficients of logistic regression: (A) power around 27 Hz; (B) LZc; both for SB− versus rest (most relevant regions as sorted by absolute coefficients).

Region names	Regression coefficient (*B*)
(A) 27 Hz	
SupraMarginal_L	−0.037
Supp_Motor_Area_R	−0.028
Occipital_L	−0.028
Precentral_R	−0.014
Rectus_L	0.007
Parietal_L	−0.007
Angular_L	−0.006
Occipital_R	−0.005
Cingulum_Post_L	−0.005
Amygdala_L	−0.001
(B) LZc	
Insula_R	−0.184
Paracentral_Lobule_R	0.166
Rectus_R	0.157
Parietal_L	0.144
Cuneus_L	0.142
Fusiform_R	−0.105
Cingulum_Post_L	−0.101
Supp_Motor_Area_R	0.100
Parietal_R	0.100
Cingulum_Ant_R	−0.097

The most important features of the multivariate logistic regression for power around 27 Hz were regions in the parietal and right precentral cortex. This broadly agrees with the findings by Dor‐Ziderman et al. (2016) and Trautwein et al. (2024) of the relevance of parietal regions. With respect to LZc, the most important regions in the multivariate logistic regression were the right insula and paracentral lobule. While the univariate *t* test indicated a widespread increase in LZc in SB− versus rest, the logistic regression coefficients for several regions were negative. This indicates that the LZc in these regions, conditioned on all other regions, showed a reduction in SB− versus rest (the same can be seen for the contrast SB− versus SB+, see Figure S1).

The disparity between the univariate and multivariate relevance of regions is likely caused by multicollinearity between regions. Correlated effects show up for each affected region in the univariate analysis, but are suppressed in the regression model because it uses the information which is contained in multiple variables only once. The feature importance values in the logistic regression model therefore represent the unique influence of each region after removing the confounding influence of correlated regions. For 27 Hz, this likely led to the suppression of some of the correlated regions, which resulted in a smaller set of regions in the multivariate analysis than in the univariate comparison. For LZc, the mismatch between univariate analysis and multivariate logistic regression suggests that SB dissolution might be mediated by a complex pattern of increases and relative decreases of different regions which cannot be detected by looking at regions individually.

Different regions are involved in oscillatory power and LZc, which might be unexpected. This is likely due to different measures capturing different aspects of brain activity. For instance, a region might show strong differences between conditions in oscillatory power without necessarily altering the signal complexity, and vice versa. However, this could also be due to methodological differences, which include the use of different beamformers (adaptive spatial filtering beamformer for oscillatory power and LCMV for LZc) and different ways of filtering the signal (oscillatory power was computed on narrow frequency bands, while LZc was computed for a wider frequency range).

### 3.5 Correlation With Lifetime Meditation Hours and Phenomenology

We expected that those meditators who had more lifetime meditation experience would be able to evoke self‐boundary dissolution better, as has been shown in previous analyses (Nave et al. 2021; Trautwein et al. 2024), and hence that classification accuracy would be higher. The correlation was tested for the within‐participant classification accuracy of the logistic regression model, as well as the average logit difference between classes, with both lifetime meditation hours and dissolution depth as quantitatively derived from the phenomenological interviews (see Nave et al. 2021; Trautwein et al. 2024 for details). This was done for 27 Hz and LZc and for the contrasts SB− versus rest and SB− versus SB+.

None of the tests showed significant results (all abs(*r*) < 0.25, *p* > 0.15, BF between 0.2 and 0.56; see correlation graphs in the Supporting Information Materials, section 6). This suggests there is likely no correlation, since Bayes factors substantially less than 1 can be interpreted as weak to moderate evidence for the absence of a correlation (Kass and Raftery 1995).

## 4 Discussion

In this study, we attempted single‐trial prediction of the meditative state of self‐boundary dissolution from MEG data. This was done both in contrast to a resting state and a control meditation state, that is, self‐boundary maintenance. We showed that a multivariate logistic regression model could differentiate self‐boundary dissolution from a resting state with well above‐chance accuracy based on source oscillatory power and LZc. The highest predictive accuracy resulted from LZc with 0.64 in the within‐participant and 0.57 in the across‐participant classification task. This is in the range of what we expected from past work on the classification of meditation states from MEG data. Zhigalov et al. (2019) showed an accuracy of ~0.6 for within‐participant and 0.55 for across‐participant classification of mindfulness versus mind‐wandering based on spectral power and connectivity. While our band power results are very similar to what Zhigalov et al. (2019) showed, we could also demonstrate that LZc allowed for a substantially more accurate distinction between SB‐ and rest. LZc might therefore be a promising feature to use for future classification studies of mental states.

Besides comparing self‐boundary dissolution to a resting state, we also compared it to a control meditation state of self‐boundary maintenance. Predictive accuracy for this contrast was substantially lower than in the comparison to rest, but still significantly above chance level for some frequency bands and for LZc. The highest accuracies again resulted from LZc, with 0.54 for within‐participant and 0.53 for across‐participants classification. This reduction in predictive performance compared to the contrast with rest is most likely due to a higher similarity between the meditation states than between a meditation and a resting state. Although SB+ was chosen as a control meditation state which maximizes the contrast with SB‐, both SB+ and SB− share the general characteristics of a meditative state such as sustained attention, awareness to the present moment, and reduced automatic or habitual thought patterns (Tang et al. 2015), and are therefore assumed to be more similar to one another than to the resting state. Since more similar states are harder to distinguish than states with stronger differences, this presumably led to a reduced predictive performance in distinguishing SB− from SB+ compared to the resting state. However, it should be acknowledged that there is a design asymmetry in the experimental protocol, as several shorter alternations between SB− and SB+ were practiced in between slightly longer resting periods. Considering that transitions between states might happen gradually rather than instantaneously, this asymmetry might have contributed to SB− being more similar to SB+ than to rest and can therefore be considered a confound not accounted for in our analysis.

The finding that most canonical frequency bands allowed for a significantly above‐chance prediction of self‐boundary dissolution suggests that the information for predicting this state is spread out over a large frequency range. This might also partly explain the higher predictive performance for classification based on LZc compared to any frequency band, as LZc is computed on a broad frequency range and therefore likely accumulates information contained in different bands. However, the broader frequency range unlikely explains entirely the higher performance of LZc compared to individual bands, as LZc captures a very different aspect of brain activity and has been shown to be a more accurate marker than band power for various conscious states (reviewed in Sarasso et al. 2021; Atad et al. 2025b). Moreover, by decomposing the periodic and aperiodic signal components, we showed that the aperiodic component has a very similar predictive accuracy as the periodic (band oscillatory power) components. This indicates that the higher accuracy in LZc was not driven by its higher sensitivity to aperiodic than to periodic signal components (Aboy et al. 2006). Additionally, the observation that the periodic, aperiodic and combined components all have very similar predictive power suggests that for the specific task of predicting self‐boundary dissolution states, there is no noteworthy advantage in decomposing the periodic and aperiodic components, even though the importance of this decomposition has been demonstrated in other contexts (Donoghue, Dominguez, and Voytek 2020).

Overall, the predictive performance was much stronger in the within‐participant classification task than in the across‐participant task. Moreover, compared to a univariate prediction based on individual brain regions, our multivariate approach constituted a substantial improvement only in the within‐participant classification task, while in the across‐participant task it showed very similar results to the univariate prediction. The greater difficulty of finding common patterns across participants is not surprising, considering that datasets differed between participants in respect to factors such as brain anatomy, sensor position, and meditative depth and experience. On the other hand, the across‐participant classification was based on many more observations than the within‐participant classification (~41 times more data points). This vastly larger training data set might have improved performance in the across‐participant classification. However, our results showed that an abundance of training data did not outweigh the additional difficulty in finding patterns between participants. This difficulty was in fact so great that compared to our multivariate methods, a univariate approach based on an individual brain region would have shown nearly identical results in the across‐subjects classification task. Our finding that these results replicate across different classification methods indicates that they are not specific to the logistic regression model, but rather robustly represented in the data. This is relevant for practical applications, as it suggests that a multivariate approach might only improve performance for a subject‐specific classifier, that is, when the training data are collected from the same person who receives the intervention. Moreover, the multivariate analysis showed higher classification accuracy than the univariate analysis only in the within‐participant, but not in the across‐participant task. This indicates that the above‐chance accuracy in the across‐participant task is mostly driven by effects from individual regions rather than multivariate activation patterns. Activation patterns of individual brain regions are therefore likely shared across participants, while multivariate activation patterns are individual‐specific. Commonalities at the level of brain regions may correspond to experiential features that are common across participants, while the variability in multivariate patterns likely reflects the subject‐specific dimensions of the experience. This mirrors the observation by Nave et al. (2021), who found that certain phenomenological features of self‐boundary dissolution are consistent across individuals, whereas others vary between participants.

To better understand which brain regions the classifier relied on, we used the absolute logistic regression coefficients to infer which regions were most relevant for the classification task. For the 27 Hz power we found that the classification was strongly driven by the left parietal and right frontal motor cortices, which aligns with the findings by Trautwein et al. (2024) and Dor‐Ziderman et al. (2016). These regions are part of a brain network responsible for top‐down goal‐directed attention (Corbetta and Shulman 2002), as well as a sensorimotor network connected to the bodily self (Ferri et al. 2012). Compared to the univariate analysis, the multivariate logistic regression relied on a smaller set of regions, not considering the posterior medial and medial parietal cortices where Trautwein et al. (2024) and Dor‐Ziderman et al. (2016) found a strong effect. The likely reason for this is that brain regions in the univariate analysis were strongly correlated and therefore contained very similar information. This allowed the logistic regression model to extract this information from only a subset of these regions.

With respect to LZc, the most relevant regions were the right insula and paracentral lobule. The insula is part of the salience network and has been associated with a variety of functions, including interoceptive processing, affective processing, and self‐awareness (Craig 2011; Uddin et al. 2017), which are closely related to the phenomenology of boundary dissolution. The paracentral lobule has mostly been associated with motor control and is part of the sensorimotor network, which has been suggested as central to representing the bodily self (Ferri et al. 2012; Patra et al. 2021). Beyond the relevance of individual brain regions, it is noteworthy that regression coefficients were positive for some regions and negative for others, that is, the likelihood of boundary dissolution increased with a higher LZc in some brain regions and decreased in other regions. This is broadly in line with the univariate results from Atad et al. (2025a), which found that self‐boundary dissolution was associated with a widespread increase in broadband LZc, alongside specific reductions. While the current analysis also found widespread increases in LZc, the specific reductions found by Atad et al. were mainly in the orbitofrontal cortex, while in our analysis they were localized in the insula, fusiform gyrus, and cingulum. The current analysis therefore adds resolution to the univariate analysis by Atad et al. by allowing us to better discern the specific regions in which either a decrease or increase in LZc contributes to predicting self‐boundary dissolution states.

The observation that different brain regions are involved in changes in oscillatory power and LZc could be due to various factors. On a functional level, signal complexity and oscillatory power might also reflect independent aspects of brain activity and are not necessarily correlated. For example, changes in oscillatory power can occur in a signal without necessarily changing LZc, and vice versa. The results for oscillatory power and LZc should therefore be seen as complementary descriptions of the brain state during self‐boundary dissolution which might capture different aspects of it. Moreover, it should be noted that different methods were used to localize sources (adaptive spatial filtering beamformer for oscillatory power, LCMV beamformer for LZc), which might yield different inverse solutions for the source localization. Additionally, LZc was computed on a broad frequency range and it is therefore expected to show different results from the oscillatory power in narrower frequency bands.

A further difference between the current multivariate analysis and the univariate analyses by Trautwein et al. (2024) and Atad et al. (2025a) is that we did not find any significant correlations between the logistic regression outcomes and meditative experience or phenomenological depth of dissolution. The univariate analyses, on the other hand, showed significant correlations between orbitofrontal LZc reduction and depth of dissolution (Atad et al. 2025a), as well as between 27 Hz oscillatory power reductions in the posterior medial cortex and both depth of dissolution and meditation experience (Trautwein et al. 2024). A possible reason for this is that aggregating signals across regions may have reduced sensitivity to localized effects that drove the univariate correlations. Alternatively, the regional weighting in the multivariate models may have captured a more distributed and global neural signature of self‐boundary dissolution which is less directly aligned with meditative experience or nuanced phenomenology of boundary‐dissolution.

Limitations of our study include the following: First, datasets had some inhomogeneities between participants. These included differences between participants such as head shape and head position relative to the sensors, as well as large variety in meditation experience (between 115 and 24.837 h, mean = 3832 ± 4845 h). The former likely impacted the accuracy of source localization across participants, while the latter raises the question of how consistently participants could access the meditative states and how comparable they are across participants. While classification performance was not positively correlated with lifetime practice hours or dissolution depth, the high variability in meditation experience between participants potentially had a negative impact on the across‐participant classification performance. To improve comparability between participants, future studies could co‐register MEG data with individual brain anatomies instead of using a standard head model. Moreover, variability in meditation experience could be reduced by recruiting groups with more homogenous experience levels or by implementing standardized measures of meditative depth. A further limitation of the current study is the use of simple classification methods. While these methods were chosen deliberately to allow for easy interpretation and because we did not have sufficient data for deep learning, it remains an open question how well more advanced classification methods would have performed. In the context of M/EEG data, deep learning methods have been successfully applied to various mental states (reviewed in Craik et al. 2019) and we would expect it to improve performance on predicting self‐boundary states as well. While the current study has shown the feasibility of single‐trial prediction of self‐boundary states based on simple methods, real‐world applications would likely benefit from the potential performance improvement of deep learning methods. More advanced methods might also be applied in respect to improving the across‐participant transferability, as the challenge of generalizing classification models across individuals is well recognized in the brain–computer interface (BCI) literature (Lotte et al. 2018; Apicella et al. 2024). Inter‐subject variability in neural representations and recording conditions often limits the transferability of models trained on one group to another. Approaches such as feature‐space alignment and transfer learning approaches have been suggested to address this issue by reducing inter‐subject variability (He and Wu 2020; Zanini et al. 2018) and might be promising methods to improving the across‐participant generalization of meditation‐state classifiers in future research. Finally, it remains to be seen how our findings generalize to other styles of meditation practice, as well as to the phenomenology of an altered sense of self in contexts other than meditation. Participants in our study had been trained by the same meditation teacher and followed the same instructions on how to dissolve their self‐boundaries. However, the phenomenology of an altered sense of self has been described in many different contemplative traditions (Wahbeh et al. 2018) and in contexts outside contemplative practices such as psychedelic experiences (Milliére et al. 2018) and in psychopathology (e.g., Sass and Parnas 2013; Sierra and David 2011; Whitmer 2001). Examining the neurophenomenology of self‐boundaries in a wider context would substantially strengthen the translational value of our work.

In summary, we demonstrated that self‐boundary dissolution can be distinguished from rest and a control meditation state with significantly above‐chance accuracy for both within‐participant and across‐participant classification. Our multivariate approach constituted a substantial improvement in predictive performance compared to predictions based on individual brain regions only in the within‐participant task, but not across participants. The best predictive performance was achieved by using LZc, which indicates that this might be a promising feature to be used in future studies on single‐trial prediction of mental states. Moreover, the brain regions that the classifiers relied on suggest that self‐boundary dissolution is mediated by complex patterns of brain activity across different regions. Future studies might be able to improve predictive performance by using individualized anatomical head models and a participant cohort of more homogeneous experience, as well as relying on more advanced machine learning methods. The feasibility of predicting the state of self‐boundaries from brain recordings holds the potential for a range of applications such as neurofeedback and assistance in meditation training.

## Funding

The research was supported by the Tiny Blue Dot (TBD) foundation grant 43777846 and the Bial Foundation grant 293/20. H.R. was funded by a PhD scholarship from the Heinrich Böll Foundation.

## Conflicts of Interest

The authors declare no conflicts of interest.

## Supporting information


**Data S1:** hbm70440‐sup‐0001‐Supplementary_materials.docx.

## Data Availability

The data that support the findings of this study are available on request from the corresponding author. The data are not publicly available due to privacy or ethical restrictions.

## References

[hbm70440-bib-0001] Aboy, M. , R. Hornero , D. Abasolo , and D. Alvarez . 2006. “Interpretation of the Lempel‐Ziv Complexity Measure in the Context of Biomedical Signal Analysis.” IEEE Transactions on Biomedical Engineering 53, no. 11: 2282–2288. 10.1109/TBME.2006.883696. 17073334

[hbm70440-bib-0002] Ahsan, M. M. , M. A. P. Mahmud , P. K. Saha , K. D. Gupta , and Z. Siddique . 2021. “Effect of Data Scaling Methods on Machine Learning Algorithms and Model Performance.” Technologies 9, no. 3: 3. 10.3390/technologies9030052.

[hbm70440-bib-0003] Apicella, A. , P. Arpaia , G. D'Errico , et al. 2024. “Toward Cross‐Subject and Cross‐Session Generalization in EEG‐Based Emotion Recognition: Systematic Review, Taxonomy, and Methods.” Neurocomputing 604: 128354. 10.1016/j.neucom.2024.128354.

[hbm70440-bib-0005] Atad, D. A. , P. A. M. Mediano , F. E. Rosas , and A. Berkovich‐Ohana . 2025a. “Meditation and Complexity: A Review and Synthesis of Evidence.” Neuroscience of Consciousness 2025, no. 1: niaf013. 10.1093/nc/niaf013. 40438122 PMC12118461

[hbm70440-bib-0078] Atad, D. A. , P. A. Mediano , F.‐M. Trautwein , et al. 2025b. “Synergistic Correlates of Self‐Dissolution in Meditation: Global Increases and Selective Reductions in Neural Complexity (No. yhavc_v1).” PsyArXiv. 10.31234/osf.io/yhavc_v1.

[hbm70440-bib-0006] Ataria, Y. 2015. “Where Do We End and Where Does the World Begin? The Case of Insight Meditation.” Philosophical Psychology 28, no. 8: 1128–1146. 10.1080/09515089.2014.969801.

[hbm70440-bib-0007] Ataria, Y. , Y. Dor‐Ziderman , and A. Berkovich‐Ohana . 2015. “How Does It Feel to Lack a Sense of Boundaries? A Case Study of a Long‐Term Mindfulness Meditator.” Consciousness and Cognition 37: 133–147. 10.1016/j.concog.2015.09.002. 26379087

[hbm70440-bib-0008] Benjamini, Y. , and Y. Hochberg . 1995. “Controlling the False Discovery Rate: A Practical and Powerful Approach to Multiple Testing.” Journal of the Royal Statistical Society. Series B, Statistical Methodology 57, no. 1: 289–300. 10.1111/j.2517-6161.1995.tb02031.x.

[hbm70440-bib-0081] Berkovich‐Ohana, A. , Y. Dor‐Ziderman , F.‐M. Trautwein , et al. 2020. “The Hitchhiker's Guide to Neurophenomenology – The Case of Studying Self Boundaries With Meditators.” Frontiers in Psychology 11. 10.3389/fpsyg.2020.01680. PMC7385412 32793056

[hbm70440-bib-0009] Blanke, O. , and T. Metzinger . 2009. “Full‐Body Illusions and Minimal Phenomenal Selfhood.” Trends in Cognitive Sciences 13, no. 1: 7–13. 10.1016/j.tics.2008.10.003. 19058991

[hbm70440-bib-0010] Brandmeyer, T. , and A. Delorme . 2013. “Meditation and Neurofeedback.” Frontiers in Psychology 4: 688. 10.3389/fpsyg.2013.00688. 24109463 PMC3791377

[hbm70440-bib-0011] Carota, F. , J.‐M. Schoffelen , R. Oostenveld , and P. Indefrey . 2022. “The Time Course of Language Production as Revealed by Pattern Classification of MEG Sensor Data.” Journal of Neuroscience 42, no. 29: 5745–5754. 10.1523/JNEUROSCI.1923-21.2022. 35680410 PMC9302460

[hbm70440-bib-0012] Casali, A. G. , O. Gosseries , M. Rosanova , et al. 2013. “A Theoretically Based Index of Consciousness Independent of Sensory Processing and Behavior.” Science Translational Medicine 5, no. 198: 198ra105. 10.1126/scitranslmed.3006294. 23946194

[hbm70440-bib-0013] Chawla, N. V. , K. W. Bowyer , L. O. Hall , and W. P. Kegelmeyer . 2002. “SMOTE: Synthetic Minority Over‐Sampling Technique.” Journal of Artificial Intelligence Research 16: 321–357. 10.1613/jair.953.

[hbm70440-bib-0014] Chen, J. C. C. , and D. A. Ziegler . 2025. “Closed‐Loop Systems and Real‐Time Neurofeedback in Mindfulness Meditation Research.” Biological Psychiatry: Cognitive Neuroscience and Neuroimaging 10, no. 4: 377–383. 10.1016/j.bpsc.2024.10.012. 39481470 PMC13050520

[hbm70440-bib-0015] Corbetta, M. , and G. L. Shulman . 2002. “Control of Goal‐Directed and Stimulus‐Driven Attention in the Brain.” Nature Reviews Neuroscience 3, no. 3: 201–215. 10.1038/nrn755. 11994752

[hbm70440-bib-0016] Craig, A. D. 2011. “Significance of the Insula for the Evolution of Human Awareness of Feelings From the Body.” Annals of the New York Academy of Sciences 1225, no. 1: 72–82. 10.1111/j.1749-6632.2011.05990.x. 21534994

[hbm70440-bib-0017] Craik, A. , Y. He , and J. L. Contreras‐Vidal . 2019. “Deep Learning for Electroencephalogram (EEG) Classification Tasks: A Review.” Journal of Neural Engineering 16, no. 3: 031001. 10.1088/1741-2552/ab0ab5. 30808014

[hbm70440-bib-0018] Damasio, A. 1999. The Feeling of What Happens: Body and Emotion in the Making of Consciousness. Harcourt Brace and Co.

[hbm70440-bib-0019] Deane, G. , M. Miller , and S. Wilkinson . 2020. “Losing Ourselves: Active Inference, Depersonalization, and Meditation.” Frontiers in Psychology 11: 539726. 10.3389/fpsyg.2020.539726. 33250804 PMC7673417

[hbm70440-bib-0020] Donoghue, T. , J. Dominguez , and B. Voytek . 2020. “Electrophysiological Frequency Band Ratio Measures Conflate Periodic and Aperiodic Neural Activity.” eNeuro 7, no. 6. 10.1523/ENEURO.0192-20.2020. PMC7768281 32978216

[hbm70440-bib-0021] Donoghue, T. , M. Haller , E. J. Peterson , et al. 2020. “Parameterizing Neural Power Spectra Into Periodic and Aperiodic Components.” Nature Neuroscience 23, no. 12: 1655–1665. 10.1038/s41593-020-00744-x. 33230329 PMC8106550

[hbm70440-bib-0022] Dor‐Ziderman, Y. , Y. Ataria , S. Fulder , A. Goldstein , and A. Berkovich‐Ohana . 2016. “Self‐Specific Processing in the Meditating Brain: A MEG Neurophenomenology Study.” Neuroscience of Consciousness 2016, no. 1: niw019. 10.1093/nc/niw019. 30397512 PMC6210398

[hbm70440-bib-0023] Ferri, F. , F. Frassinetti , M. Ardizzi , M. Costantini , and V. Gallese . 2012. “A Sensorimotor Network for the Bodily Self.” Journal of Cognitive Neuroscience 24, no. 7: 1584–1595. 10.1162/jocn_a_00230. 22452562

[hbm70440-bib-0024] Fisher, R. A. 1992. “Statistical Methods for Research Workers.” In Breakthroughs in Statistics: Methodology and Distribution, edited by S. Kotz and N. L. Johnson , 66–70. Springer. 10.1007/978-1-4612-4380-9_6.

[hbm70440-bib-0026] Gallagher, S. 2000. “Philosophical Conceptions of the Self: Implications for Cognitive Science.” Trends in Cognitive Sciences 4, no. 1: 14–21. 10.1016/S1364-6613(99)01417-5. 10637618

[hbm70440-bib-0027] Gerrans, P. 2019. “Depersonalization Disorder, Affective Processing and Predictive Coding.” Review of Philosophy and Psychology 10, no. 2: 401–418. 10.1007/s13164-018-0415-2.

[hbm70440-bib-0028] Gerster, M. , G. Waterstraat , V. Litvak , et al. 2022. “Separating Neural Oscillations From Aperiodic 1/f Activity: Challenges and Recommendations.” Neuroinformatics 20, no. 4: 991–1012. 10.1007/s12021-022-09581-8. 35389160 PMC9588478

[hbm70440-bib-0056] Gross, J. , J. Kujala , M. Hämäläinen , L. Timmermann , A. Schnitzler , and R. Salmelin . 2001. “Dynamic Imaging of Coherent Sources: Studying Neural Interactions in the Human Brain.” Proceedings of the National Academy of Sciences 98, no. 2: 694–699. 10.1073/pnas.98.2.694. PMC14650 11209067

[hbm70440-bib-0029] Hall, M. A. , and G. Holmes . 2003. “Benchmarking Attribute Selection Techniques for Discrete Class Data Mining.” IEEE Transactions on Knowledge and Data Engineering 15, no. 6: 1437–1447. 10.1109/TKDE.2003.1245283.

[hbm70440-bib-0030] Halme, H.‐L. , and L. Parkkonen . 2016. “Comparing Features for Classification of MEG Responses to Motor Imagery.” PLoS One 11, no. 12: e0168766. 10.1371/journal.pone.0168766. 27992574 PMC5161474

[hbm70440-bib-0031] Hanley, A. W. , M. Dambrun , and E. L. Garland . 2020. “Effects of Mindfulness Meditation on Self‐Transcendent States: Perceived Body Boundaries and Spatial Frames of Reference.” Mindfulness 11, no. 5: 1194–1203. 10.1007/s12671-020-01330-9. 33747250 PMC7968136

[hbm70440-bib-0032] He, H. , and D. Wu . 2020. “Transfer Learning for Brain–Computer Interfaces: A Euclidean Space Data Alignment Approach.” IEEE Transactions on Biomedical Engineering 67, no. 2: 399–410. 10.1109/TBME.2019.2913914. 31034407

[hbm70440-bib-0033] Huttunen, H. , T. Manninen , J.‐P. Kauppi , and J. Tohka . 2013. “Mind Reading With Regularized Multinomial Logistic Regression.” Machine Vision and Applications 24, no. 6: 1311–1325. 10.1007/s00138-012-0464-y.

[hbm70440-bib-0082] Jin, C. Y. , J. P. Borst , and M. K. van Vugt . 2019. “Predicting Task‐General Mind‐Wandering With EEG.” Cognitive, Affective, & Behavioral Neuroscience 19, no. 4: 1059–1073. 10.3758/s13415-019-00707-1. PMC6711882 30850931

[hbm70440-bib-0034] Kabat‐Zinn, J. 1990. Full Catastrophe Living: Using the Wisdom of Your Body and Mind to Face Stress, Pain, and Illness. Random House Publishing Group.

[hbm70440-bib-0035] Kaspar, F. , and H. G. Schuster . 1987. “Easily Calculable Measure for the Complexity of Spatiotemporal Patterns.” Physical Review A 36, no. 2: 842–848. 10.1103/PhysRevA.36.842. 9898930

[hbm70440-bib-0036] Kass, R. E. , and A. E. Raftery . 1995. “Bayes Factors.” Journal of the American Statistical Association 90, no. 430: 773–795.

[hbm70440-bib-0037] Kaushik, P. , A. Moye , M. van Vugt , and P. P. Roy . 2022. “Decoding the Cognitive States of Attention and Distraction in a Real‐Life Setting Using EEG.” Scientific Reports 12, no. 1: 20649. 10.1038/s41598-022-24417-w. 36450871 PMC9712397

[hbm70440-bib-0038] Kirberg, M. , and M. Chadha . 2024. “Depersonalization, Meditation, and the Experience of (No‐) Self.” Journal of Consciousness Studies 31, no. 5–6: 151–177. 10.53765/20512201.31.5.151.

[hbm70440-bib-0039] Kitson, A. , A. Chirico , A. Gaggioli , and B. E. Riecke . 2020. “A Review on Research and Evaluation Methods for Investigating Self‐Transcendence.” Frontiers in Psychology 11: 547687. 10.3389/fpsyg.2020.547687. 33312147 PMC7701337

[hbm70440-bib-0040] Kora, P. , K. Meenakshi , K. Swaraja , A. Rajani , and M. S. Raju . 2021. “EEG Based Interpretation of Human Brain Activity During Yoga and Meditation Using Machine Learning: A Systematic Review.” Complementary Therapies in Clinical Practice 43: 101329. 10.1016/j.ctcp.2021.101329. 33618287

[hbm70440-bib-0041] Kyselo, M. 2014. “The Body Social: An Enactive Approach to the Self.” Frontiers in Psychology 5: 986. 10.3389/fpsyg.2014.00986. PMC4162380 25309471

[hbm70440-bib-0042] Legrand, D. 2006. “The Bodily Self: The Sensori‐Motor Roots of Pre‐Reflective Self‐Consciousness.” Phenomenology and the Cognitive Sciences 5, no. 1: 89–118. 10.1007/s11097-005-9015-6.

[hbm70440-bib-0043] Lempel, A. , and J. Ziv . 1976. “On the Complexity of Finite Sequences.” IEEE Transactions on Information Theory 22, no. 1: 75–81. 10.1109/TIT.1976.1055501.

[hbm70440-bib-0044] Lin, Y. , D. A. Atad , and A. P. Zanesco . 2025. “Using Electroencephalography to Advance Mindfulness Science: A Survey of Emerging Methods and Approaches.” Biological Psychiatry: Cognitive Neuroscience and Neuroimaging 10, no. 4: 342–349. 10.1016/j.bpsc.2024.09.012. 39369988 PMC11971390

[hbm70440-bib-0045] Lotte, F. , L. Bougrain , A. Cichocki , et al. 2018. “A Review of Classification Algorithms for EEG‐Based Brain–Computer Interfaces: A 10 Year Update.” Journal of Neural Engineering 15, no. 3: 031005. 10.1088/1741-2552/aab2f2. 29488902

[hbm70440-bib-0046] Ly, A. , J. Verhagen , and E.‐J. Wagenmakers . 2016. “Harold Jeffreys's Default Bayes Factor Hypothesis Tests: Explanation, Extension, and Application in Psychology.” Journal of Mathematical Psychology 72: 19–32. 10.1016/j.jmp.2015.06.004.

[hbm70440-bib-0047] Mediano, P. A. M. , F. E. Rosas , C. Timmermann , et al. 2024. “Effects of External Stimulation on Psychedelic State Neurodynamics.” ACS Chemical Neuroscience 15, no. 3: 462–471. 10.1021/acschemneuro.3c00289. 38214686 PMC10853937

[hbm70440-bib-0048] Millière, R. , R. L. Carhart‐Harris , L. Roseman , F.‐M. Trautwein , and A. Berkovich‐Ohana . 2018. “Psychedelics, Meditation, and Self‐Consciousness.” Frontiers in Psychology 9: 1475. 10.3389/fpsyg.2018.01475. 30245648 PMC6137697

[hbm70440-bib-0049] Müller, K.‐R. , M. Tangermann , G. Dornhege , M. Krauledat , G. Curio , and B. Blankertz . 2008. “Machine Learning for Real‐Time Single‐Trial EEG‐Analysis: From Brain–Computer Interfacing to Mental State Monitoring.” Journal of Neuroscience Methods 167, no. 1: 82–90. 10.1016/j.jneumeth.2007.09.022. 18031824

[hbm70440-bib-0050] Nave, O. , F.‐M. Trautwein , Y. Ataria , et al. 2021. “Self‐Boundary Dissolution in Meditation: A Phenomenological Investigation.” Brain Sciences 11, no. 6: 6. 10.3390/brainsci11060819. PMC8235013 34205621

[hbm70440-bib-0080] Newen, A. 2018. “The Embodied Self, the Pattern Theory of Self, and the Predictive Mind.” Frontiers in Psychology 9. 10.3389/fpsyg.2018.02270. PMC6265368 30532721

[hbm70440-bib-0180] Nolte, G. 2003. “The Magnetic Lead Field Theorem in the Quasi‐Static Approximation and Its Use for Magnetoencephalography Forward Calculation in Realistic Volume Conductors.” Physics in Medicine & Biology 48, no. 22: 3637. 10.1088/0031-9155/48/22/002. 14680264

[hbm70440-bib-0051] Oostenveld, R. , P. Fries , E. Maris , and J.‐M. Schoffelen . 2011. “FieldTrip: Open Source Software for Advanced Analysis of MEG, EEG, and Invasive Electrophysiological Data.” Computational Intelligence and Neuroscience 2011: 1:1–1:9. 10.1155/2011/156869. 21253357 PMC3021840

[hbm70440-bib-0053] Patra, A. , H. Kaur , P. Chaudhary , A. Asghar , and A. Singal . 2021. “Morphology and Morphometry of Human Paracentral Lobule: An Anatomical Study With Its Application in Neurosurgery.” Asian Journal of Neurosurgery 16, no. 2: 349–354. 10.4103/ajns.AJNS_505_20. 34268163 PMC8244697

[hbm70440-bib-0055] Pedregosa, F. , G. Varoquaux , A. Gramfort , et al. 2011. “Scikit‐Learn: Machine Learning in Python.” Journal of Machine Learning Research 12, no. 85: 2825–2830.

[hbm70440-bib-0054] PMOD Technologies LLC . 2022. “PMOD: Biomedical Image Quantification Software (Version 4.4).” https://www.pmod.com.

[hbm70440-bib-0057] Reggente, N. , C. Kothe , T. Brandmeyer , et al. 2025. “Decoding Depth of Meditation: Electroencephalography Insights From Expert Vipassana Practitioners.” Biological Psychiatry Global Open Science 5, no. 1: 100402. 10.1016/j.bpsgos.2024.100402. 39660274 PMC11629179

[hbm70440-bib-0058] Saarela, M. , and S. Jauhiainen . 2021. “Comparison of Feature Importance Measures as Explanations for Classification Models.” SN Applied Sciences 3, no. 2: 272. 10.1007/s42452-021-04148-9.

[hbm70440-bib-0059] Sarasso, S. , A. G. Casali , S. Casarotto , M. Rosanova , C. Sinigaglia , and M. Massimini . 2021. “Consciousness and Complexity: A Consilience of Evidence.” Neuroscience of Consciousness 2021, no. 2: niab023. 10.1093/nc/niab023. 38496724 PMC10941977

[hbm70440-bib-0060] Sass, L. A. 2013. “Self‐Disturbance and Schizophrenia: Structure, Specificity, Pathogenesis. (Current Issues, New Directions).” Recherches en Psychanalyse 16, no. 2: 119–132. 10.3917/rep.016.0119. 23773296

[hbm70440-bib-0061] Sass, L. A. , and J. Parnas . 2003. “Schizophrenia, Consciousness, and the Self.” Schizophrenia Bulletin 29, no. 3: 427–444. 10.1093/oxfordjournals.schbul.a007017. 14609238

[hbm70440-bib-0062] Sierra, M. , and A. S. David . 2011. “Depersonalization: A Selective Impairment of Self‐Awareness.” Consciousness and Cognition 20, no. 1: 99–108. 10.1016/j.concog.2010.10.018. 21087873

[hbm70440-bib-0063] Sitaram, R. , T. Ros , L. Stoeckel , et al. 2017. “Closed‐Loop Brain Training: The Science of Neurofeedback.” Nature Reviews Neuroscience 18, no. 2: 2. 10.1038/nrn.2016.164. 28003656

[hbm70440-bib-0065] Tal, I. , and M. Abeles . 2013. “Cleaning MEG Artifacts Using External Cues.” Journal of Neuroscience Methods 217, no. 1: 31–38. 10.1016/j.jneumeth.2013.04.002. 23583420

[hbm70440-bib-0066] Tang, Y.‐Y. , B. K. Hölzel , and M. I. Posner . 2015. “The Neuroscience of Mindfulness Meditation.” Nature Reviews Neuroscience 16, no. 4: 213–225. 10.1038/nrn3916. 25783612

[hbm70440-bib-0067] Tong, F. , and M. S. Pratte . 2012. “Decoding Patterns of Human Brain Activity.” Annual Review of Psychology 63, no. 1: 483–509. 10.1146/annurev-psych-120710-100412. PMC7869795 21943172

[hbm70440-bib-0068] Trautwein, F.‐M. , Y. Schweitzer , Y. Dor‐Ziderman , et al. 2024. “Suspending the Embodied Self in Meditation Attenuates Beta Oscillations in Posterior Medial Cortex.” Journal of Neuroscience 44. 10.1523/JNEUROSCI.1182-23.2024. PMC11211716 38760162

[hbm70440-bib-0069] Tzourio‐Mazoyer, N. , B. Landeau , D. Papathanassiou , et al. 2002. “Automated Anatomical Labeling of Activations in SPM Using a Macroscopic Anatomical Parcellation of the MNI MRI Single‐Subject Brain.” NeuroImage 15, no. 1: 273–289. 10.1006/nimg.2001.0978. 11771995

[hbm70440-bib-0070] Uddin, L. Q. , J. S. Nomi , B. Hébert‐Seropian , J. Ghaziri , and O. Boucher . 2017. “Structure and Function of the Human Insula.” Journal of Clinical Neurophysiology 34, no. 4: 300–306. 10.1097/WNP.0000000000000377. 28644199 PMC6032992

[hbm70440-bib-0071] Van Veen, B. D. , and K. M. Buckley . 1988. “Beamforming: A Versatile Approach to Spatial Filtering.” IEEE ASSP Magazine 5, no. 2: 4–24. 10.1109/53.665.

[hbm70440-bib-0079] Varela, F. J. , E. Rosch , and E. Thompson . 1991. The Embodied Mind: Cognitive Science and Human Experience. MIT Press. 10.7551/mitpress/6730.001.0001.

[hbm70440-bib-0072] Wahbeh, H. , A. Sagher , W. Back , P. Pundhir , and F. Travis . 2018. “A Systematic Review of Transcendent States Across Meditation and Contemplative Traditions.” Explorer 14, no. 1: 19–35. 10.1016/j.explore.2017.07.007. 29269049

[hbm70440-bib-0074] Whitmer, G. 2001. “On the Nature of Dissociation.” Psychoanalytic Quarterly 70, no. 4: 807–837. 10.1002/j.2167-4086.2001.tb00622.x. 11678065

[hbm70440-bib-0075] Yang, W. F. Z. , T. Sparby , M. Wright , E. Kim , and M. D. Sacchet . 2024. “Volitional Mental Absorption in Meditation: Toward a Scientific Understanding of Advanced Concentrative Absorption Meditation and the Case of Jhana.” Heliyon 10, no. 10: e31223. 10.1016/j.heliyon.2024.e31223. 38803854 PMC11129010

[hbm70440-bib-0076] Zanini, P. , M. Congedo , C. Jutten , S. Said , and Y. Berthoumieu . 2018. “Transfer Learning: A Riemannian Geometry Framework With Applications to Brain–Computer Interfaces.” IEEE Transactions on Biomedical Engineering 65, no. 5: 1107–1116. 10.1109/TBME.2017.2742541. 28841546

[hbm70440-bib-0077] Zhigalov, A. , E. Heinilä , T. Parviainen , L. Parkkonen , and A. Hyvärinen . 2019. “Decoding Attentional States for Neurofeedback: Mindfulness vs. Wandering Thoughts.” NeuroImage 185: 565–574. 10.1016/j.neuroimage.2018.10.014. 30317018

