# Supplementary material for: Decoding the Self: Single‐Trial Prediction of Self‐Boundary Meditation States From Magnetoencephalography Recordings
Source: Hum Brain Mapp. 2025 Dec 26;47(1):e70440. doi: 10.1002/hbm.70440 (PMC12742028; doi:10.1002/hbm.70440)

# **Supplementary materials**

# Methods

Table 1: Definition of frequency bands, as in Trautwein et al. (2024).

| Band name | Delta | Theta | Alpha | Low beta | High beta | Low Gamma | High Gamma |
| --- | --- | --- | --- | --- | --- | --- | --- |
| Frequency  range | 1–4 Hz | 4–8 Hz | 8–12 Hz | 12–20 Hz | 20–30 Hz | 30–60 Hz | 60–90 Hz |

Table 2: Merged regions of the original AAL atlas. All other regions were as in the original AAL atlas (Tzourio-Mazoyer et al., 2002), and regions belonging to the cerebellum or vermis were excluded.

| Merged region | Sub-regions (names as in original AAL atlas) |
| --- | --- |
| Middle frontal gyrus | Frontal_Mid, Frontal_Mid_Orb, Frontal_Med_Orb |
| Superior frontal gyrus | Frontal_Sup, Frontal_Sup_Orb, Frontal_Sup_Medial |
| Inferior frontal gyrus | Frontal_Inf_Oper, Frontal_Inf_Tri, Frontal_Inf_Orb |
| Hippocampus and parahippocampus | Hippocampus, parahippocampus |
| Lateral occipital lobe | Occipital_Sup, Occipital_Mid, Occipital_Inf |
| Parietal lobe | Parietal_Sup, Parietal_Inf |
| Temporal lobe | Temporal_Sup, Temporal_Pole_Sup, Temporal_Mid, Temporal_Pole_Mid, Temporal_Inf |

# Univariate and multivariate classification results


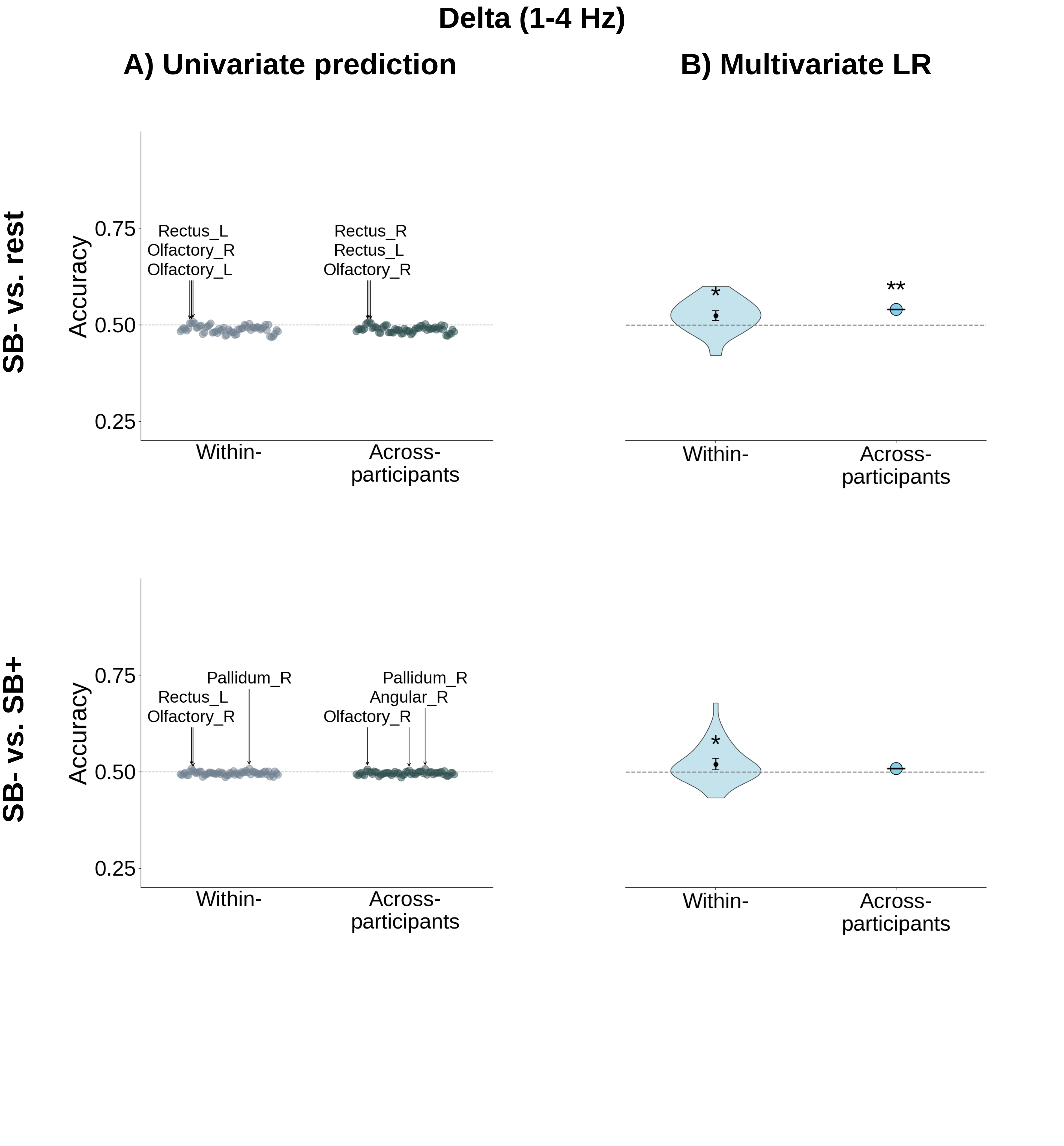


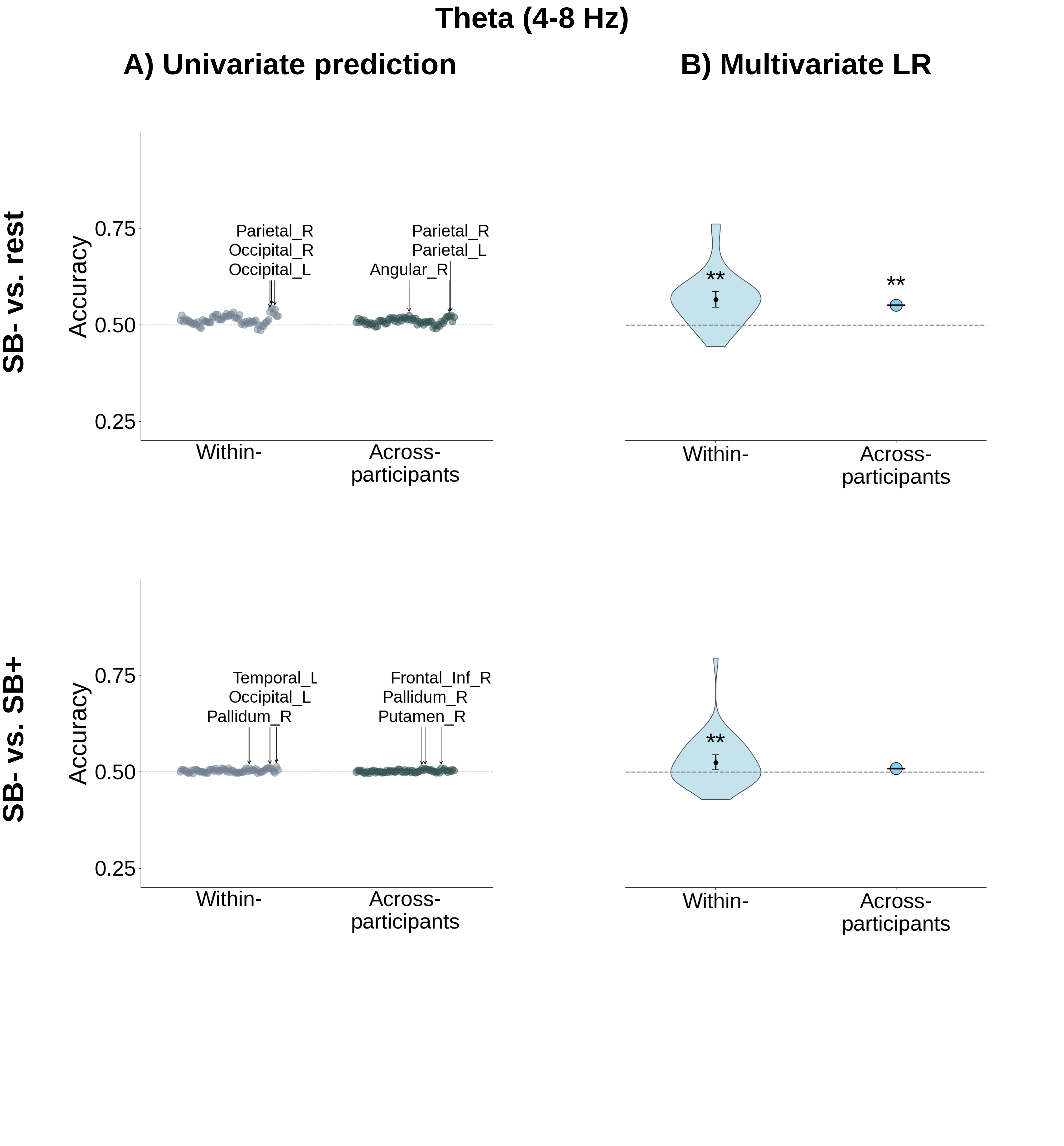


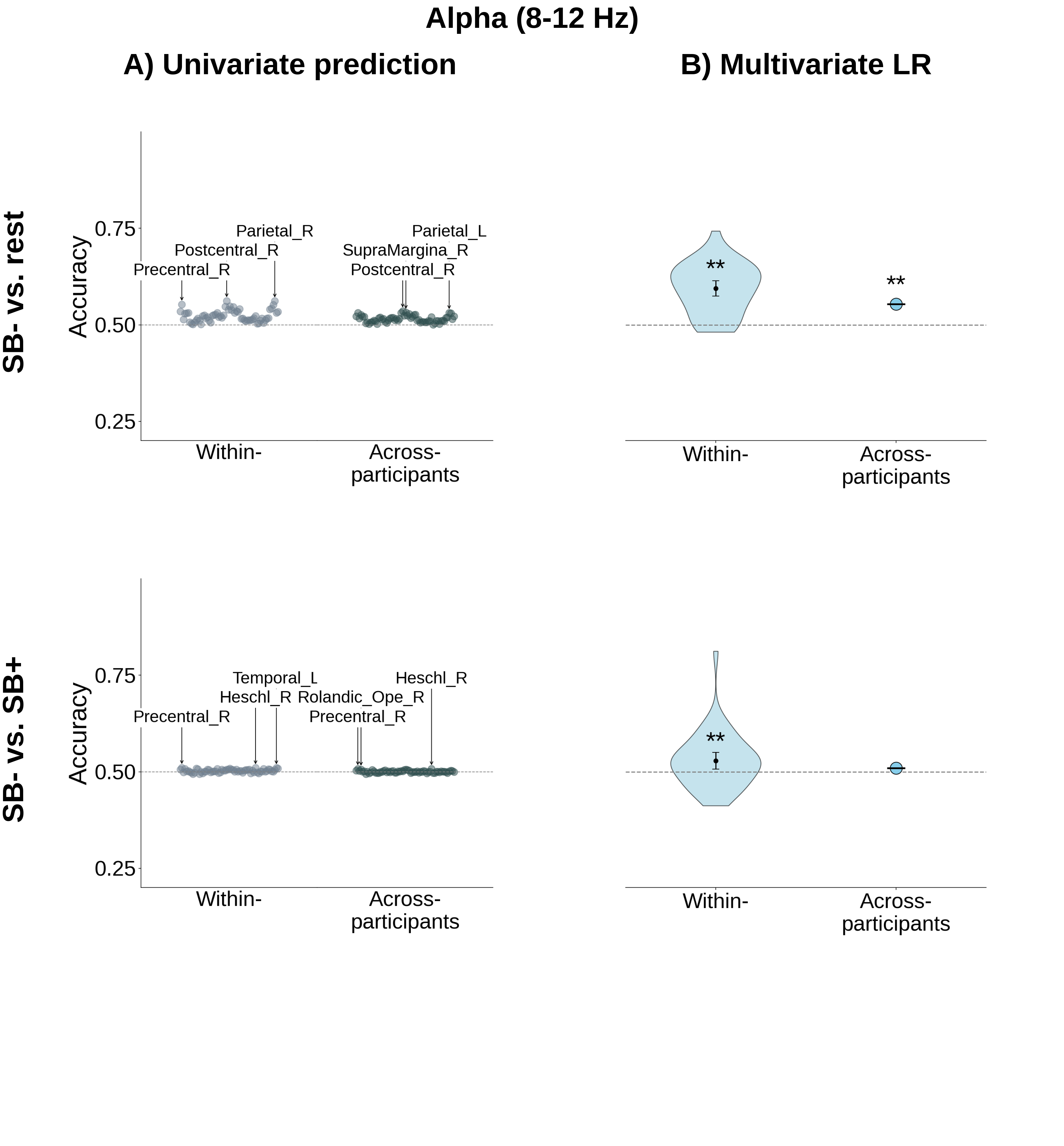


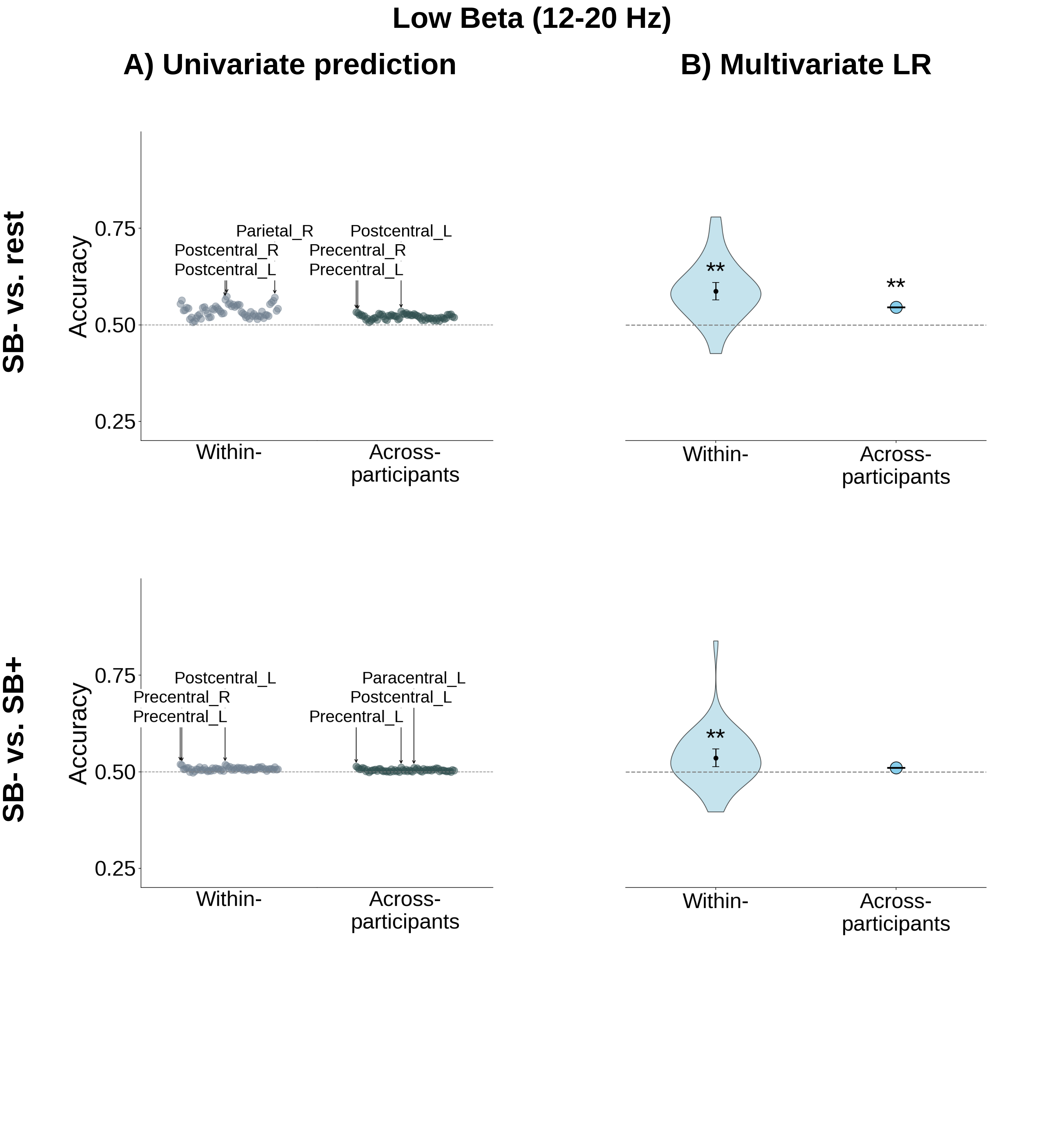


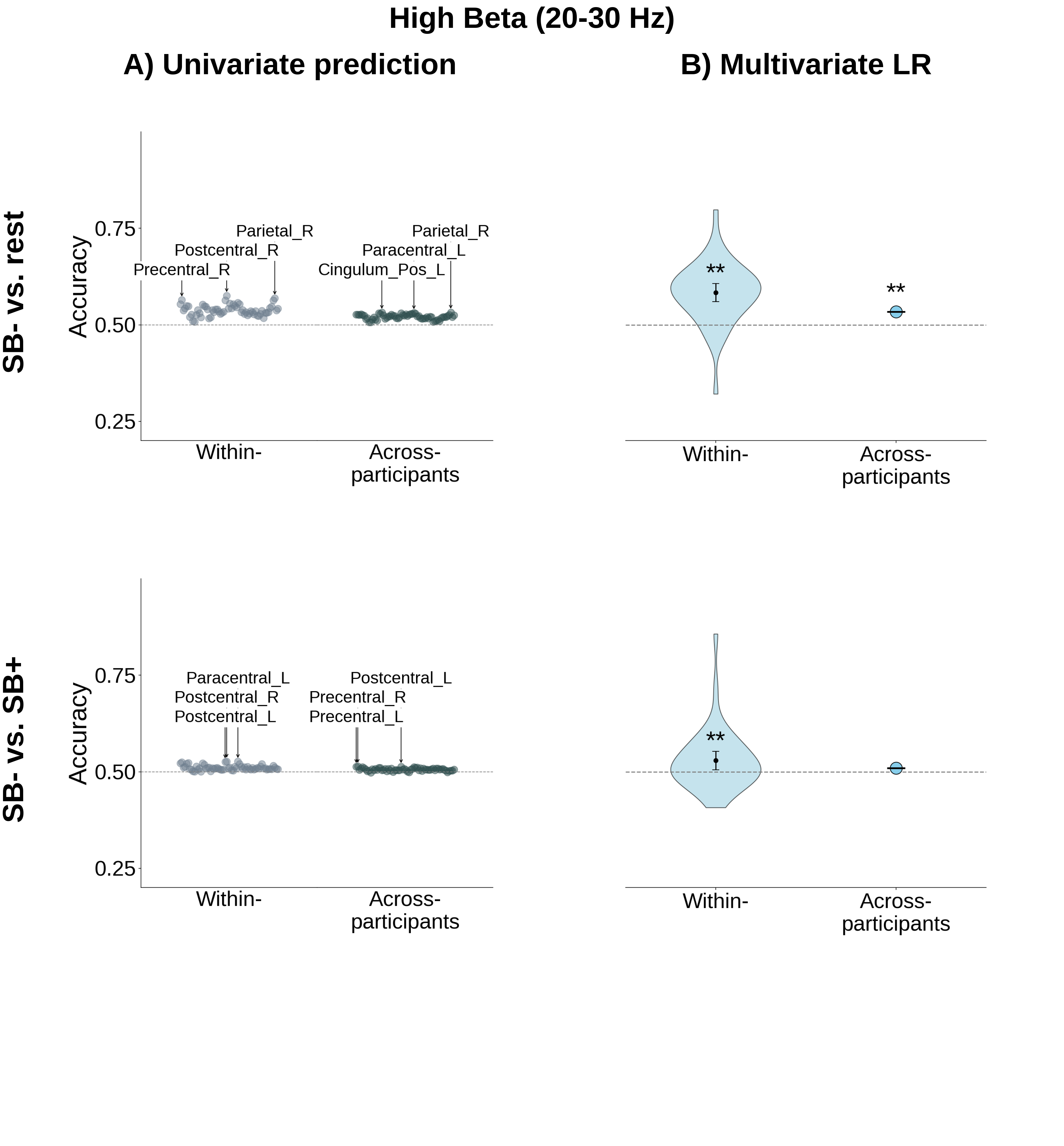


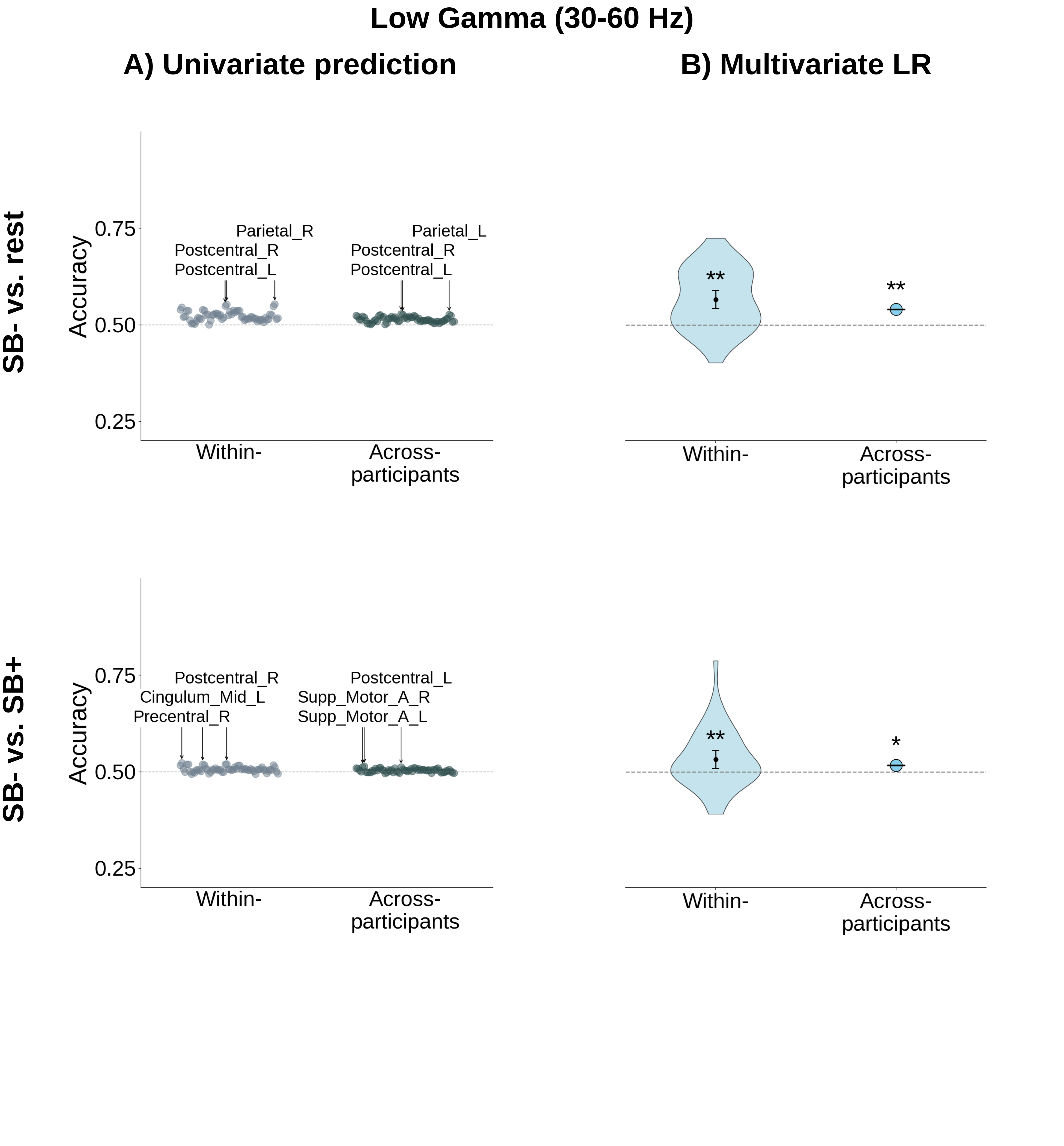


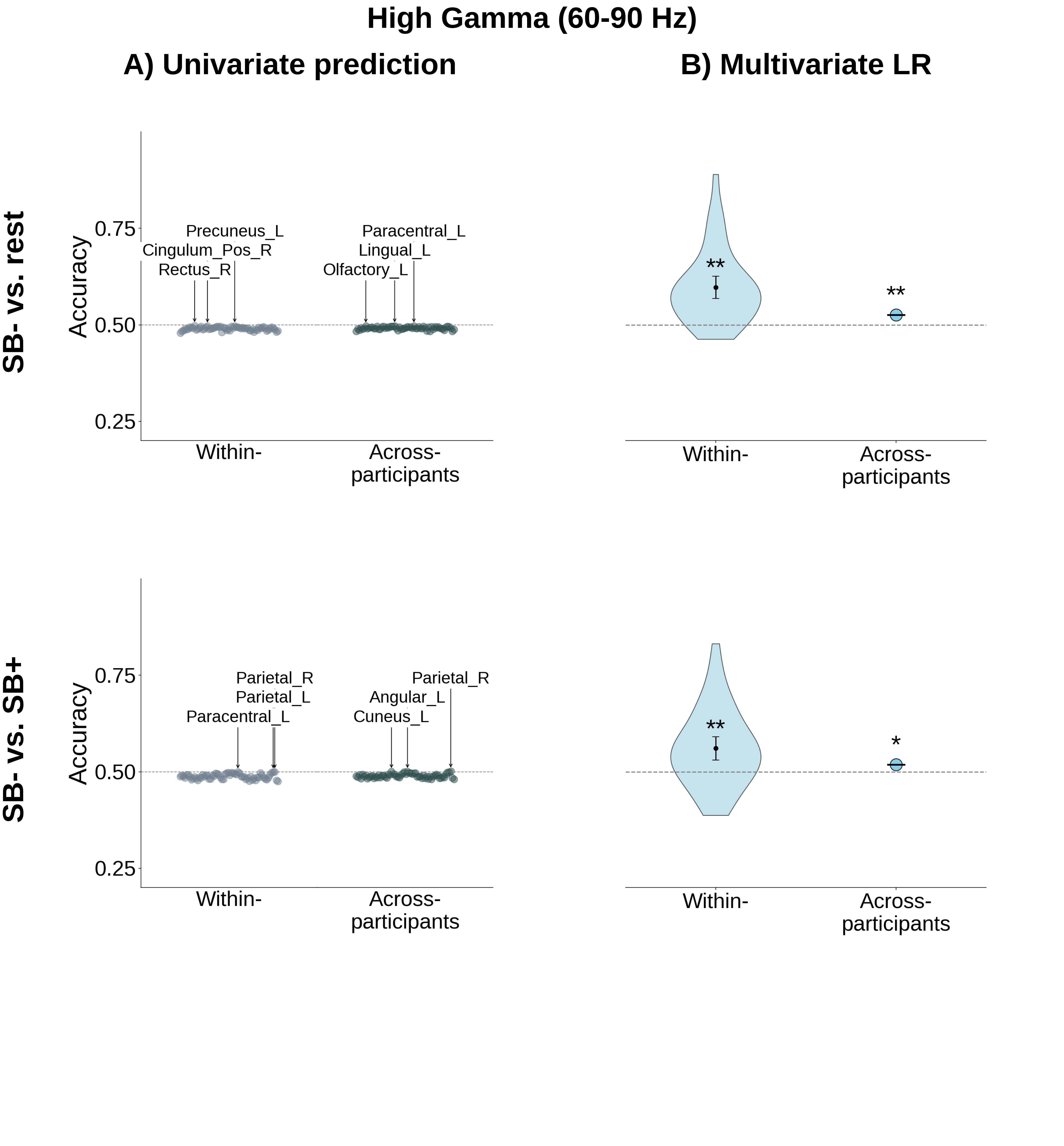


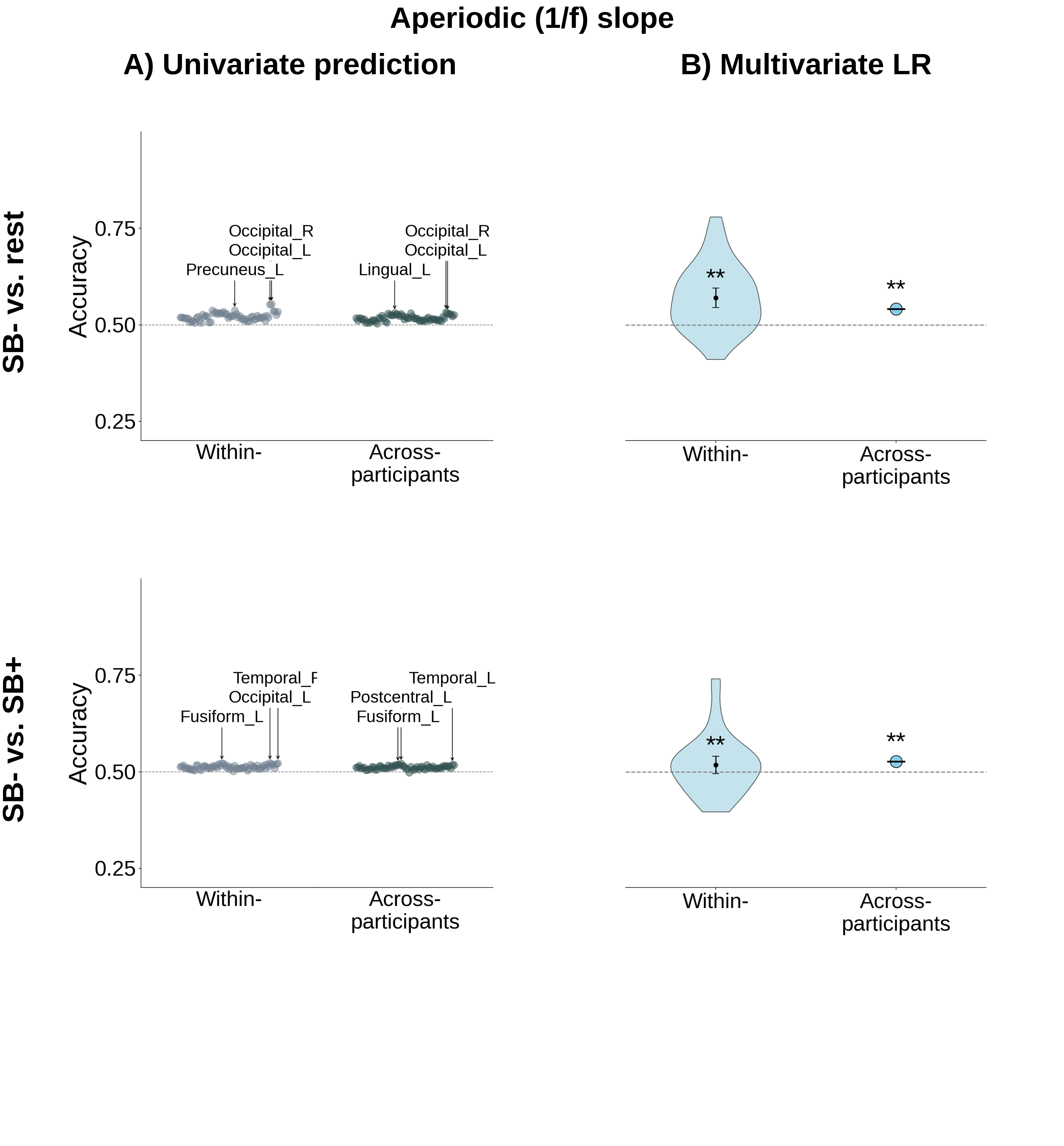


# Region-wise assessment of the contrast between classes

**A)** Cohen’s d effect size across all participants. **B)** Feature importance as measured by the logistic regression coefficients.


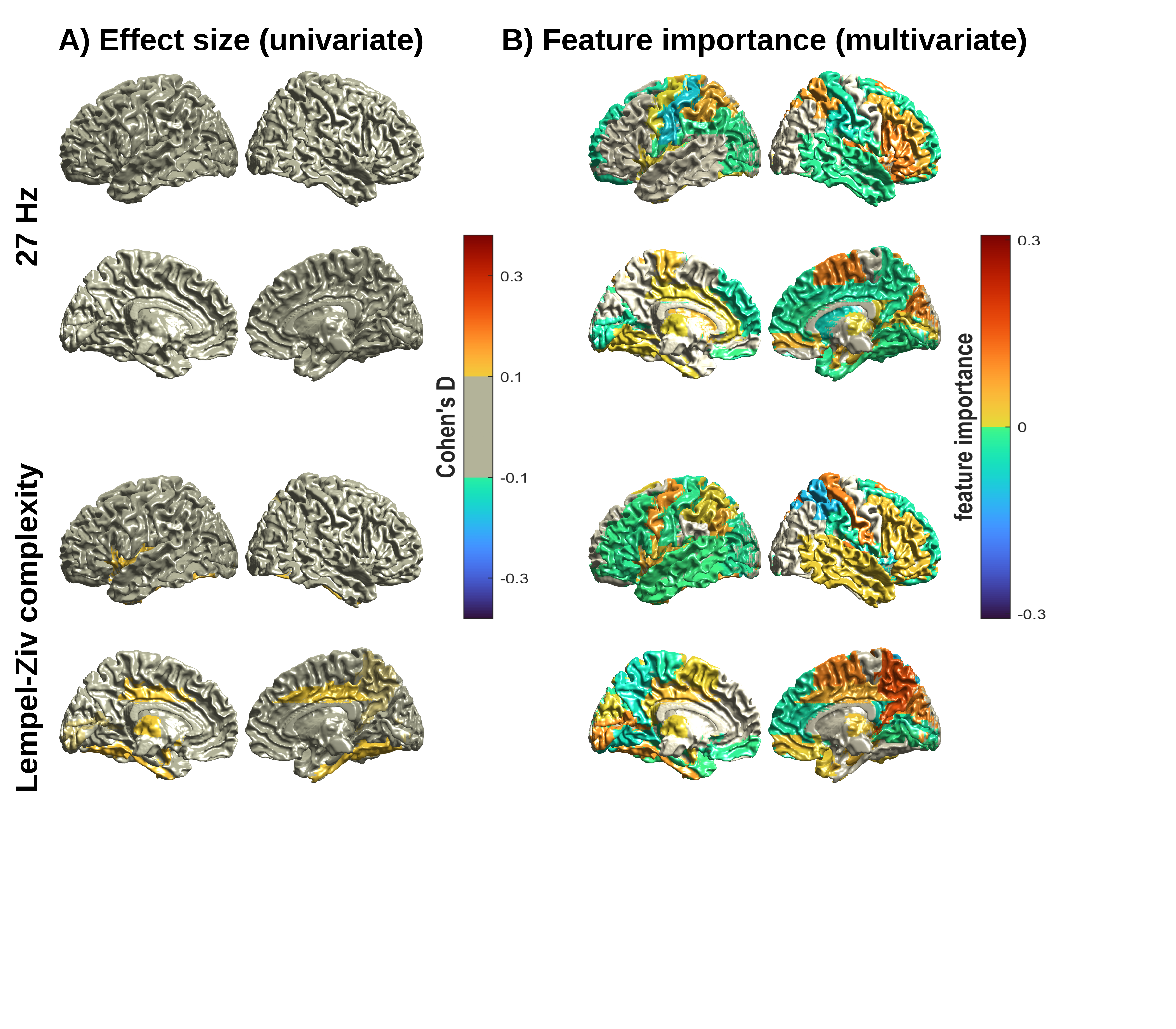
Figure 1: Contrast SB- vs. SB+


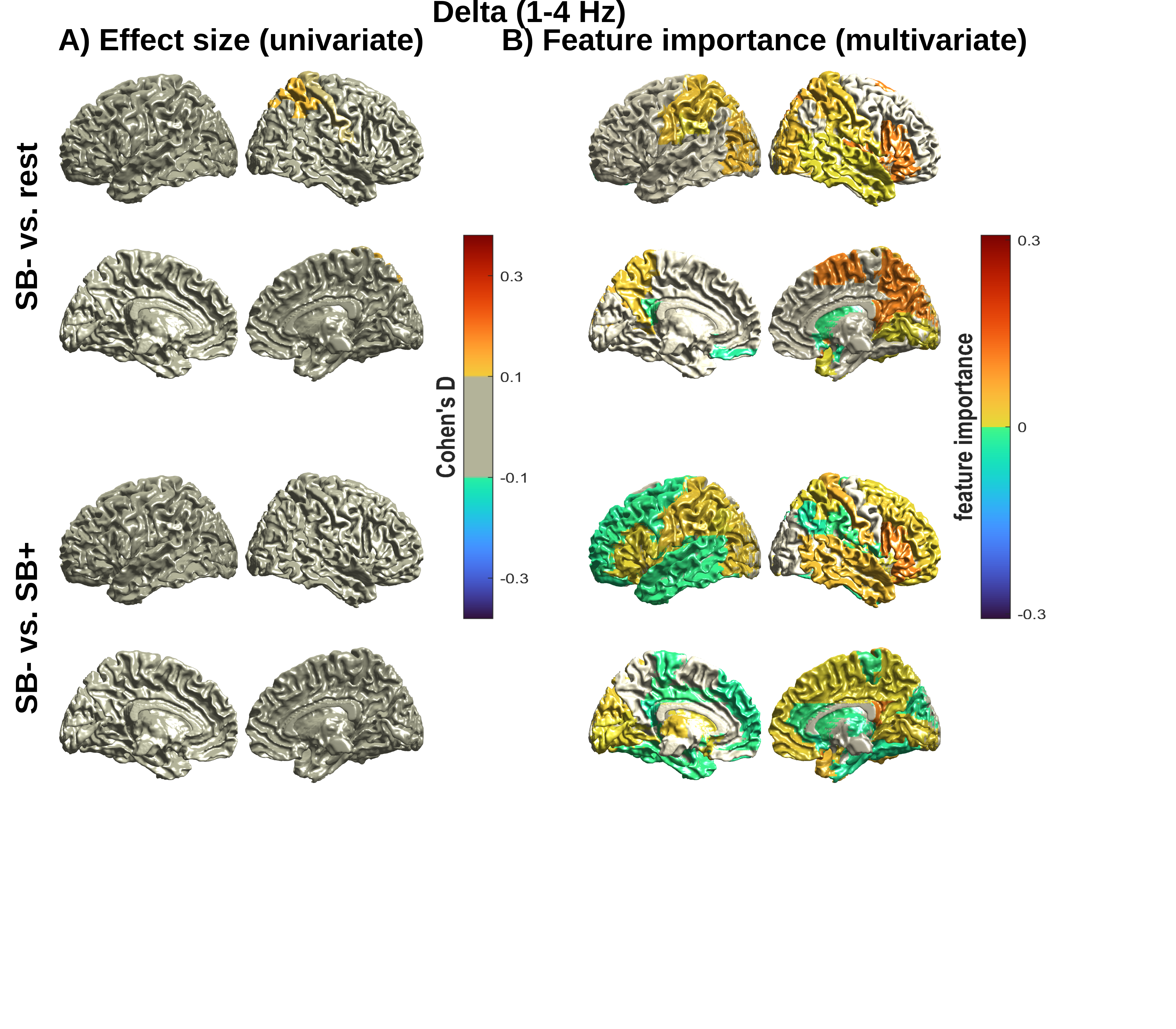


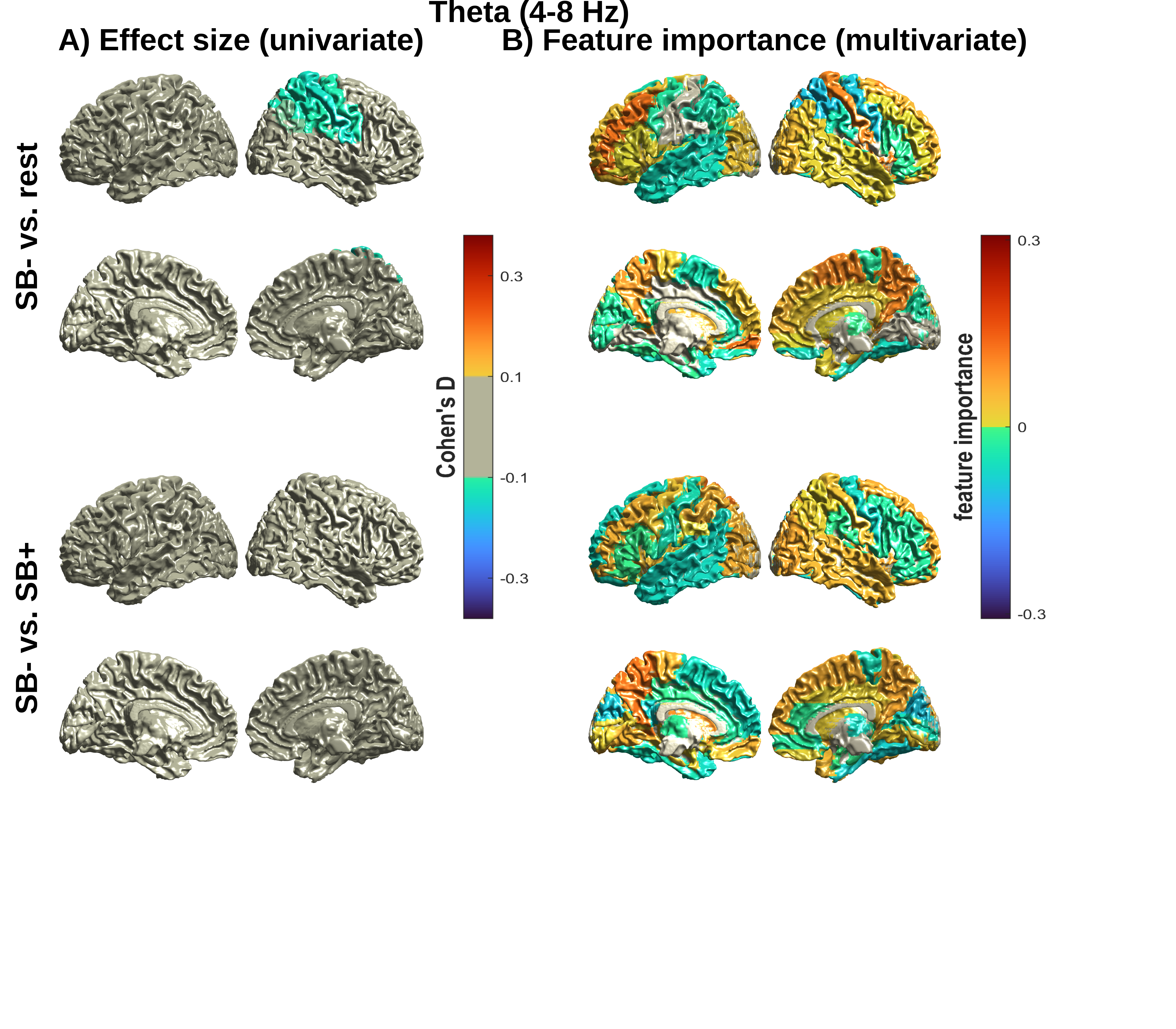


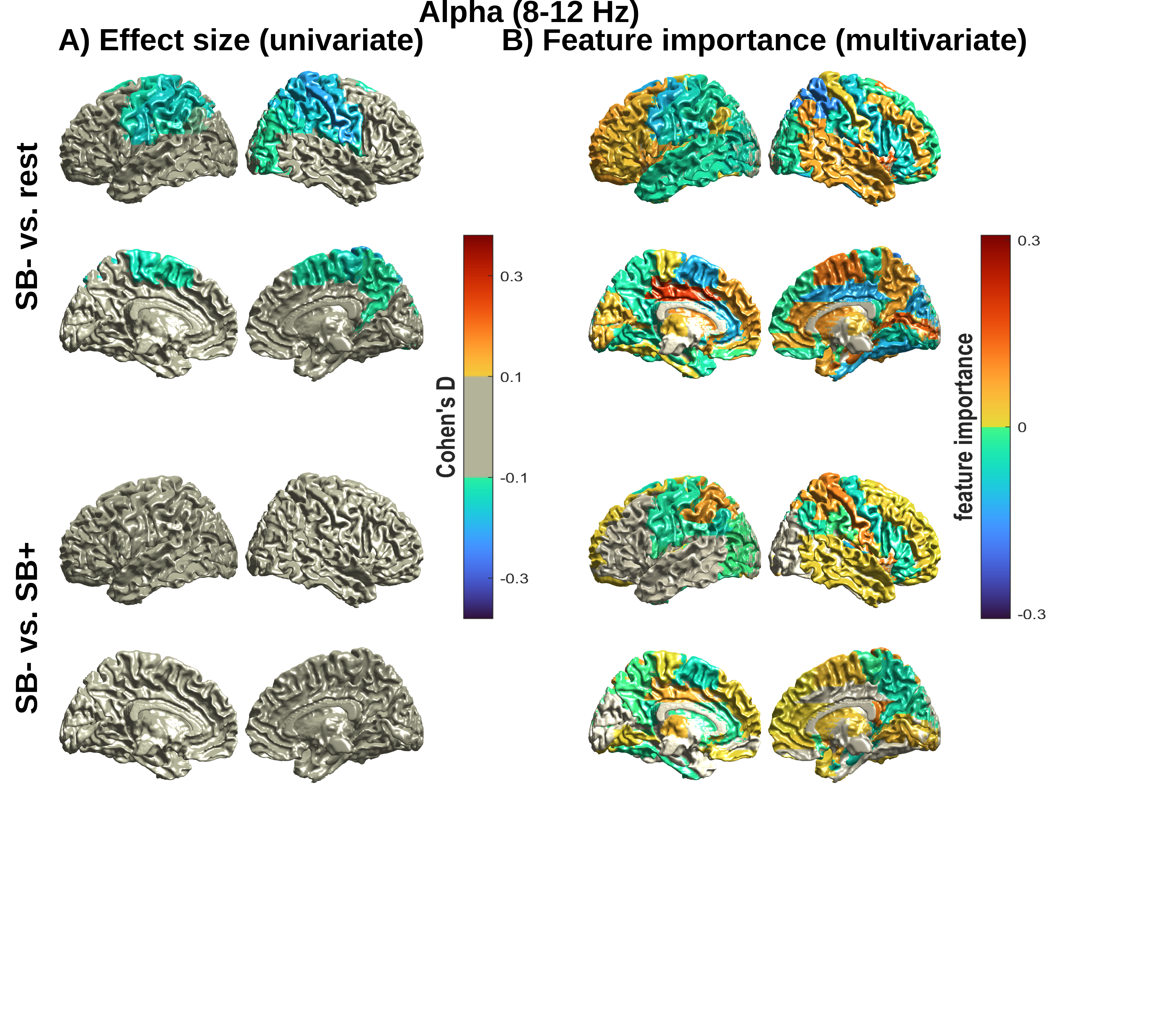


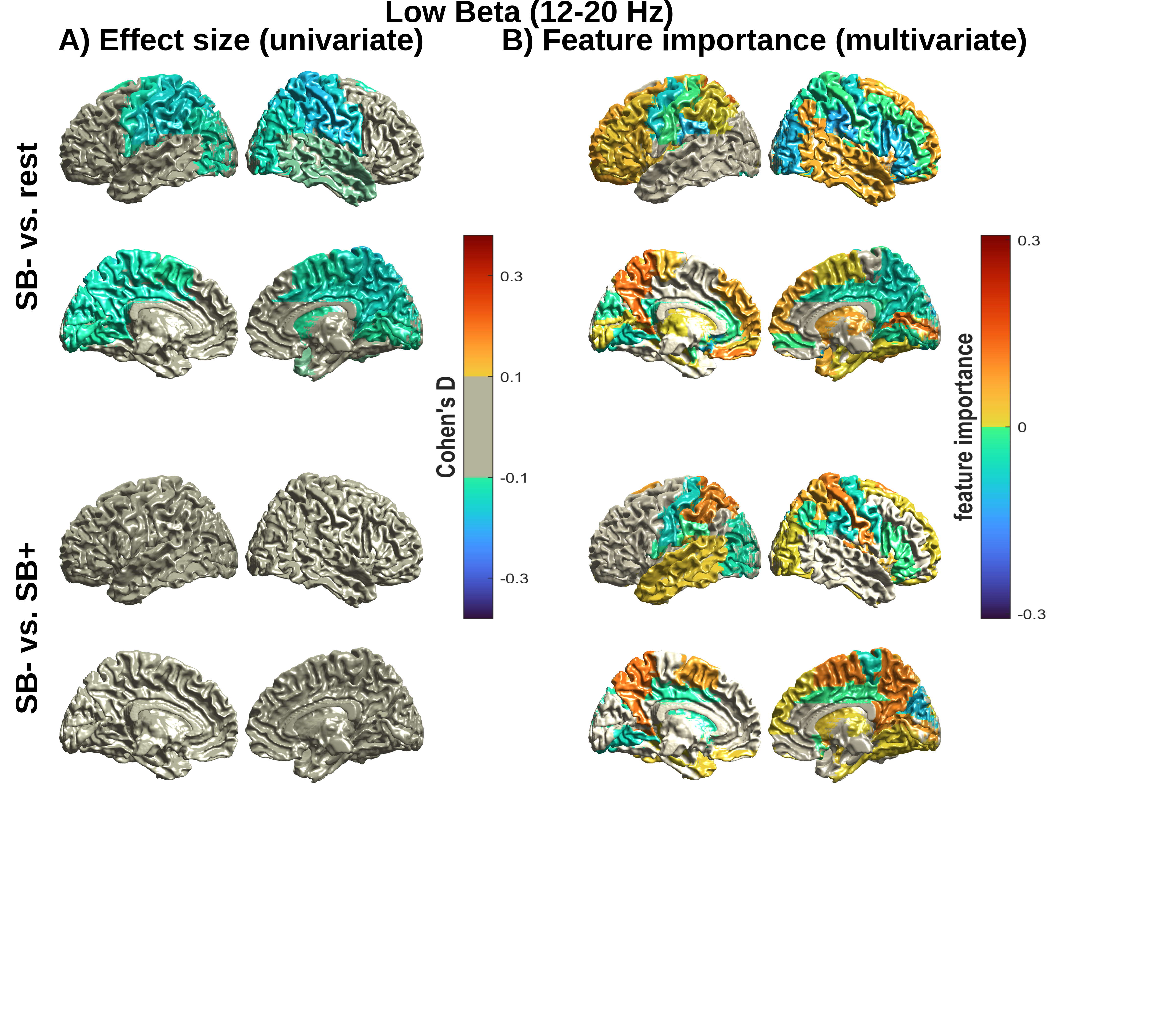


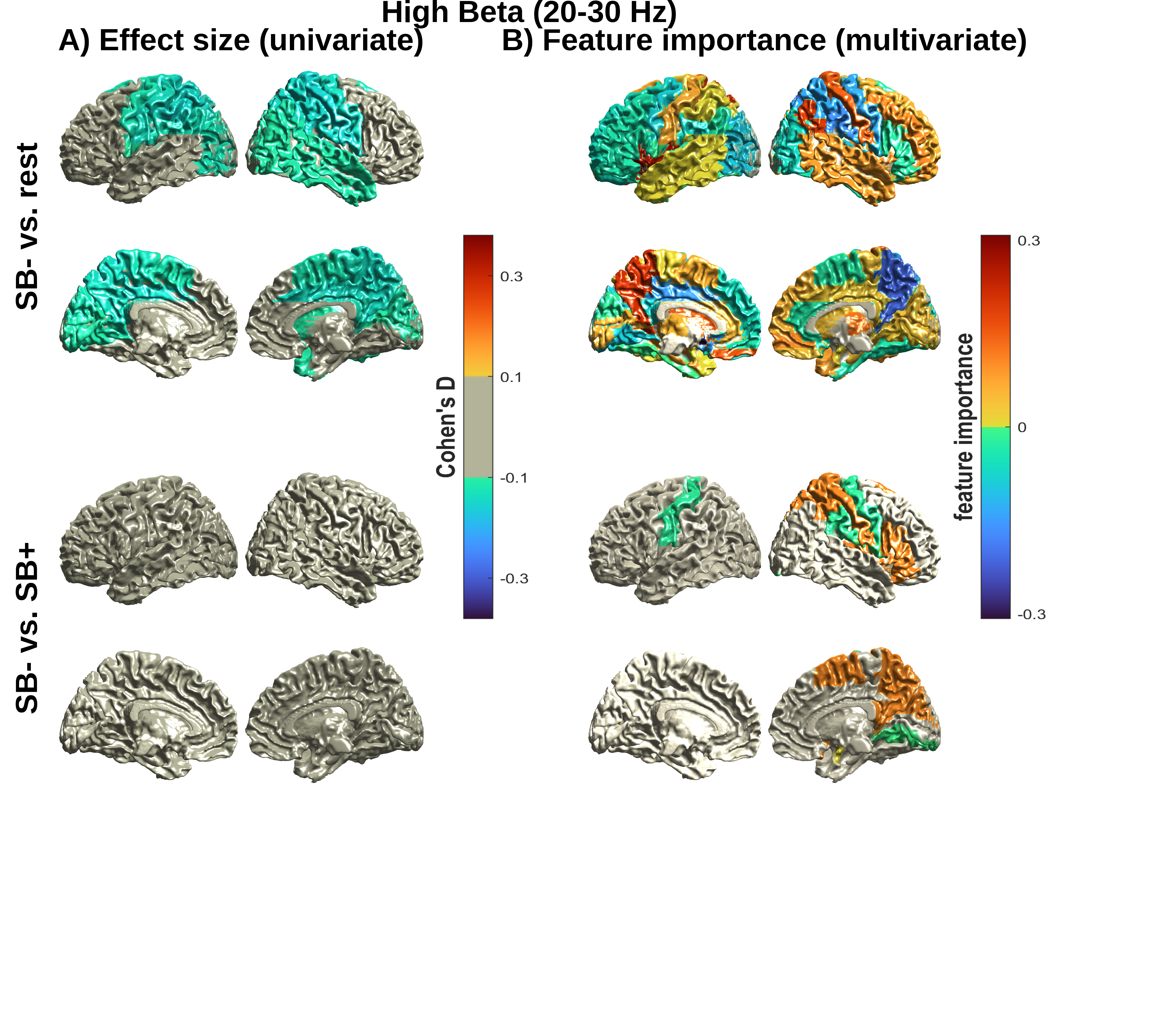


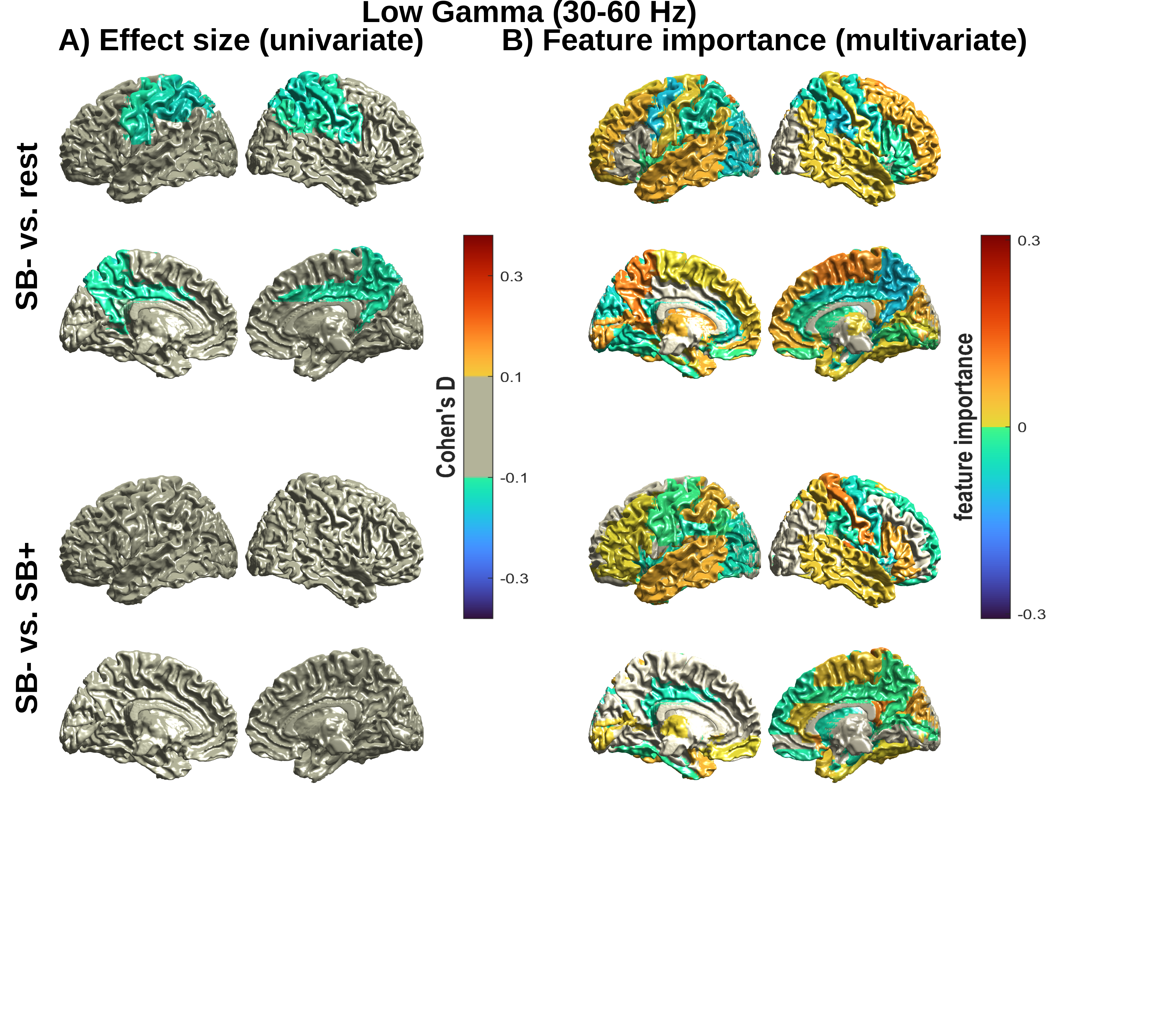


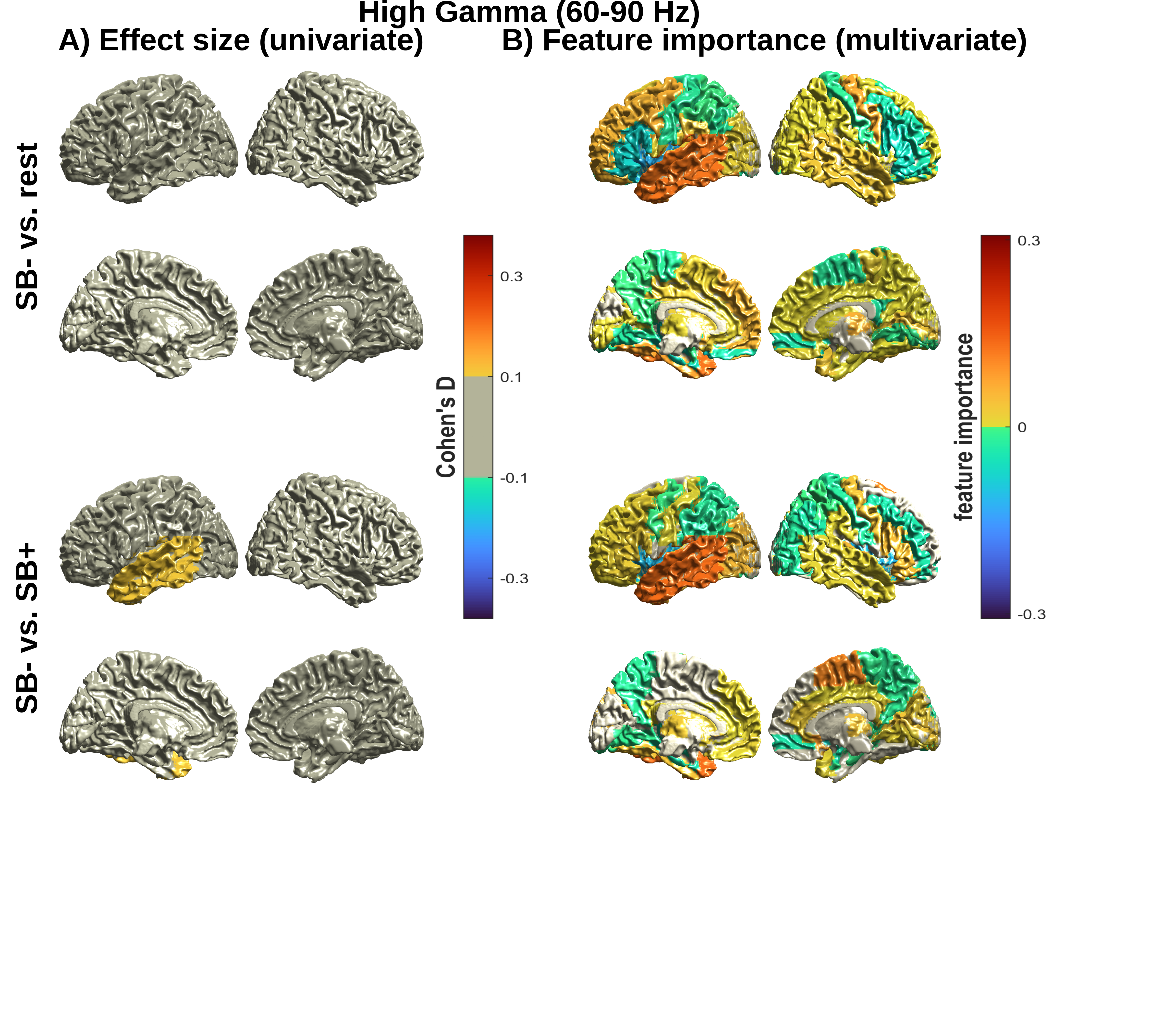


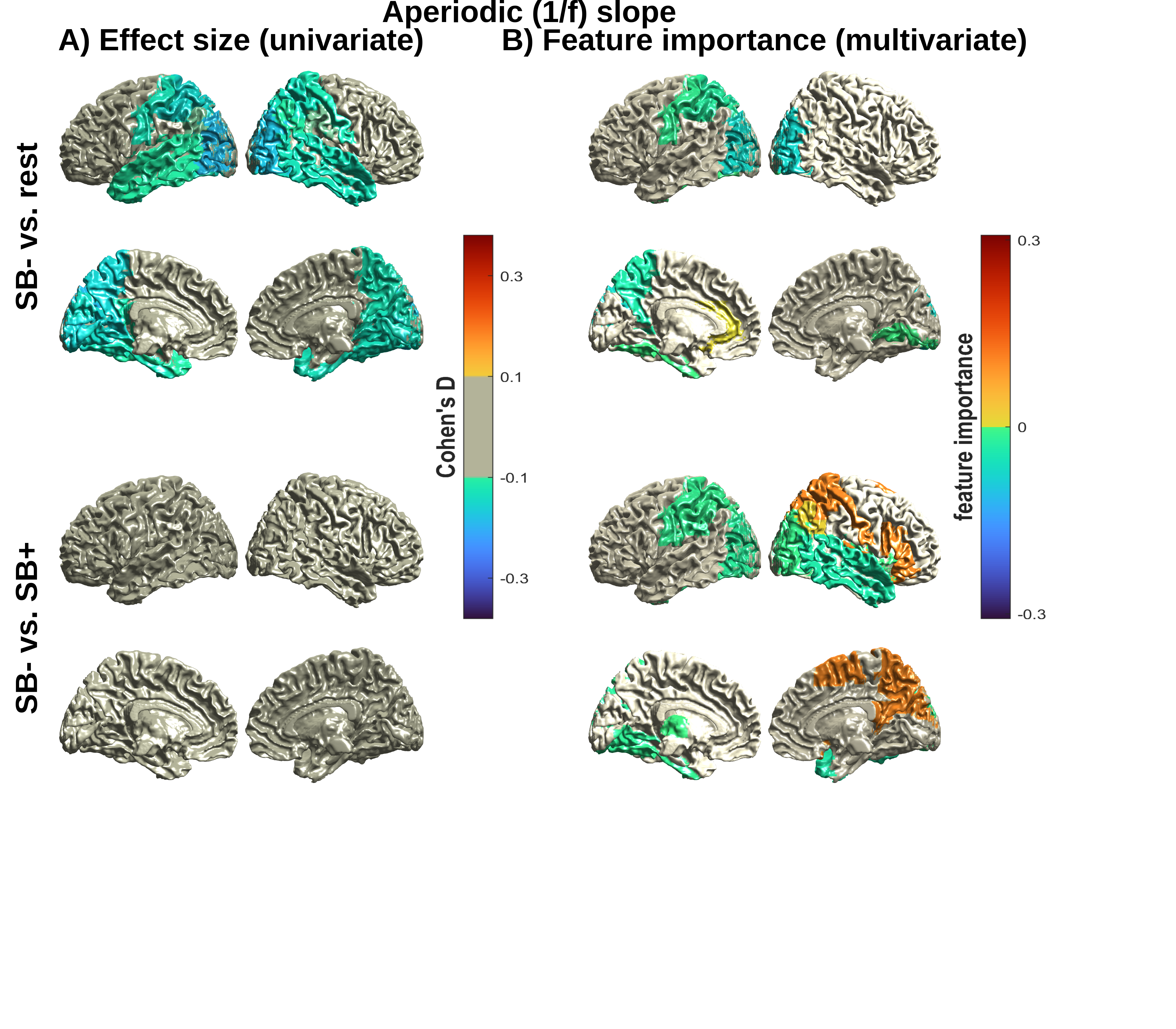


# Hyper- and model parameters

|  | LZc, rest | LZc, maintenance | 27 Hz, rest | 27 Hz, maintenance | Delta, rest | Delta, maintenance |
| --- | --- | --- | --- | --- | --- | --- |
| C | 0.1 | 0.1 | 0.01 | 0.1 | 0.01 | 0.1 |
| β0 (intercept) | 0 | 0 | 0 | 0.001 | 0 | 0 |
| β1 | 0.04 | 0.076 | 0 | 0.002 | 0 | -0.014 |
| β2 | 0.03 | 0 | -0.014 | 0 | 0 | 0 |
| β3 | -0.03 | 0.026 | 0 | -0.011 | 0 | 0 |
| β4 | -0.07 | -0.015 | 0 | -0.003 | 0.002 | -0.007 |
| β5 | 0 | 0.014 | 0 | 0 | 0 | 0 |
| β6 | 0 | 0 | -0.028 | 0 | 0 | 0.003 |
| β7 | 0 | -0.03 | 0 | 0 | 0 | 0.004 |
| β8 | 0 | 0.012 | 0 | 0.03 | -0.02 | -0.033 |
| β9 | 0.06 | -0.017 | 0.007 | -0.003 | -0.022 | 0 |
| β10 | 0.16 | 0.017 | 0 | 0 | 0 | 0.014 |
| β11 | -0.03 | 0.051 | 0 | 0.015 | 0 | 0.016 |
| β12 | -0.18 | -0.074 | 0 | 0 | 0 | 0.002 |
| β13 | -0.07 | 0 | 0 | 0.01 | 0 | 0 |
| β14 | -0.1 | -0.04 | 0 | -0.018 | 0 | -0.005 |
| β15 | 0 | 0.045 | 0 | 0.01 | 0 | -0.002 |
| β16 | 0 | 0.056 | 0 | -0.031 | 0 | 0.008 |
| β17 | -0.1 | 0.012 | -0.005 | 0 | -0.004 | -0.027 |
| β18 | 0.03 | -0.058 | 0 | 0.011 | 0 | 0 |
| β19 | 0.01 | 0.025 | -0.001 | 0 | 0 | -0.016 |
| β20 | 0.03 | 0 | 0 | 0.047 | -0.021 | 0.013 |
| β21 | 0 | 0.087 | 0 | -0.033 | 0 | 0 |
| β22 | -0.04 | -0.031 | 0 | 0.021 | 0.008 | 0.013 |
| β23 | 0.14 | 0.007 | 0 | 0 | 0 | 0.02 |
| β24 | 0.02 | 0 | 0 | 0 | 0 | -0.015 |
| β25 | 0 | -0.056 | 0 | 0.012 | 0 | 0.007 |
| β26 | 0 | -0.007 | 0 | -0.014 | 0.002 | 0.013 |
| β27 | 0.07 | 0.075 | 0 | 0.01 | 0 | -0.004 |
| β28 | -0.11 | 0 | -0.014 | -0.008 | 0 | -0.022 |
| β29 | 0 | -0.014 | 0 | -0.089 | 0.03 | 0.026 |
| β30 | 0.06 | 0 | 0 | -0.031 | 0.022 | 0.048 |
| β31 | 0.01 | 0 | -0.037 | -0.012 | 0.005 | 0.024 |
| β32 | 0 | -0.034 | -0.055 | -0.057 | 0.016 | -0.002 |
| β33 | 0.08 | 0.054 | -0.006 | -0.009 | 0 | 0.002 |
| β34 | 0.05 | 0 | 0 | 0 | 0 | -0.029 |
| β35 | -0.03 | -0.055 | 0 | 0 | 0.026 | 0 |
| β36 | 0 | 0.156 | -0.012 | -0.024 | 0 | 0.006 |
| β37 | 0.02 | -0.031 | 0 | 0.033 | 0 | -0.011 |
| β38 | 0.17 | 0 | 0 | 0 | 0 | -0.001 |
| β39 | 0.01 | 0 | 0 | 0.048 | 0 | 0.006 |
| β40 | 0 | 0 | 0 | -0.064 | -0.005 | -0.004 |
| β41 | -0.1 | 0 | 0 | 0 | 0 | -0.001 |
| β42 | 0.04 | -0.025 | 0 | -0.008 | 0 | -0.022 |
| β43 | 0 | -0.01 | 0 | -0.043 | 0 | -0.013 |
| β44 | 0 | -0.016 | 0 | -0.014 | 0 | -0.005 |
| β45 | 0.07 | 0.013 | 0 | 0.007 | 0 | 0.023 |
| β46 | 0.08 | 0.032 | 0 | 0.01 | 0 | -0.017 |
| β47 | 0 | -0.011 | 0 | 0.015 | 0 | -0.007 |
| β48 | 0.02 | 0 | 0 | 0 | 0 | -0.001 |
| β49 | -0.03 | -0.007 | 0 | 0 | 0 | -0.007 |
| β50 | 0.09 | 0.031 | 0 | 0.047 | 0 | 0.022 |
| β51 | 0 | 0 | 0 | -0.034 | 0 | -0.019 |
| β52 | -0.08 | -0.032 | 0 | -0.025 | 0 | 0.009 |
| β53 | 0 | -0.017 | 0 | 0 | 0 | 0.008 |
| β54 | 0.09 | 0.028 | 0 | 0 | 0 | 0 |
| β55 | 0 | -0.013 | 0 | 0 | 0 | -0.001 |
| β56 | 0 | 0.042 | 0 | 0.029 | 0 | -0.021 |
| β57 | 0.03 | -0.022 | -0.028 | -0.015 | 0.041 | 0.023 |
| β58 | 0.01 | 0 | -0.005 | 0 | 0.033 | 0 |
| β59 | 0.14 | 0.011 | -0.007 | 0.049 | 0.027 | 0.019 |
| β60 | 0 | -0.11 | 0 | 0.07 | 0.038 | 0.006 |
| β61 | 0.07 | -0.001 | 0 | 0 | 0 | -0.009 |
| β62 | 0.02 | 0.018 | 0 | -0.016 | 0.001 | 0.037 |

|  | Theta, rest | Theta, maintenance | Alpha, rest | Alpha, maintenance | Low Beta, rest | Low Beta, maintenance |
| --- | --- | --- | --- | --- | --- | --- |
| C | 0.1 | 10 | 1 | 0.1 | 0.1 | 0.1 |
| β0 (intercept) | -0.01 | 0 | -0.01 | 0 | 0.001 | 0.003 |
| β1 | -0.03 | 0.03 | -0.106 | -0.032 | -0.079 | 0 |
| β2 | -0.09 | -0.07 | -0.089 | -0.06 | -0.076 | -0.06 |
| β3 | 0.01 | -0.03 | 0.065 | -0.011 | 0 | -0.021 |
| β4 | 0 | -0.02 | -0.093 | 0.003 | -0.083 | 0 |
| β5 | -0.05 | -0.06 | -0.116 | -0.051 | 0 | 0.066 |
| β6 | 0 | 0.06 | 0.11 | 0.034 | 0.008 | 0 |
| β7 | 0.04 | 0.01 | 0.06 | 0.016 | -0.103 | 0 |
| β8 | 0 | -0.04 | -0.02 | -0.013 | 0 | -0.008 |
| β9 | -0.04 | 0.03 | -0.003 | 0.009 | 0.113 | 0.022 |
| β10 | -0.06 | 0.01 | -0.026 | 0 | 0 | 0 |
| β11 | 0.02 | 0.04 | 0.069 | 0 | 0.033 | 0 |
| β12 | 0.1 | 0.07 | 0.147 | 0.101 | 0.071 | 0.037 |
| β13 | -0.04 | -0.02 | -0.114 | -0.019 | -0.01 | 0 |
| β14 | 0.03 | -0.02 | 0.074 | 0.013 | 0 | 0 |
| β15 | 0 | -0.01 | 0.208 | 0.063 | 0 | -0.028 |
| β16 | 0.02 | 0.01 | -0.123 | 0 | -0.047 | -0.007 |
| β17 | -0.02 | -0.08 | -0.049 | -0.035 | -0.036 | 0 |
| β18 | 0.02 | 0.03 | -0.036 | 0 | -0.02 | 0 |
| β19 | -0.02 | 0.01 | -0.001 | 0 | -0.005 | 0 |
| β20 | 0 | 0 | -0.004 | 0.048 | 0.003 | 0.018 |
| β21 | -0.03 | 0.01 | 0.047 | 0 | 0.007 | 0 |
| β22 | 0 | -0.05 | 0.141 | 0.022 | 0.115 | 0.07 |
| β23 | -0.02 | -0.09 | 0.032 | 0 | -0.024 | 0 |
| β24 | -0.04 | -0.1 | -0.118 | -0.032 | -0.048 | -0.103 |
| β25 | 0 | 0.05 | -0.038 | 0.012 | -0.055 | -0.06 |
| β26 | 0 | 0.02 | -0.053 | 0.042 | -0.03 | 0.013 |
| β27 | -0.01 | -0.05 | 0.009 | -0.024 | 0 | 0 |
| β28 | -0.06 | -0.08 | -0.113 | 0 | 0.003 | 0.011 |
| β29 | 0 | -0.05 | -0.065 | -0.031 | -0.001 | -0.068 |
| β30 | 0 | 0.03 | 0.017 | 0 | -0.005 | 0 |
| β31 | 0 | 0 | -0.068 | -0.022 | -0.108 | -0.008 |
| β32 | -0.05 | -0.01 | -0.052 | -0.001 | -0.123 | -0.069 |
| β33 | -0.03 | -0.07 | 0.023 | -0.055 | 0.002 | 0 |
| β34 | 0.02 | 0.02 | 0.081 | -0.038 | 0.074 | -0.028 |
| β35 | 0.08 | 0.12 | -0.032 | -0.002 | 0.124 | 0.119 |
| β36 | 0 | 0.07 | 0.066 | -0.043 | -0.06 | 0 |
| β37 | 0.02 | 0.05 | 0.011 | 0.003 | 0.039 | 0 |
| β38 | -0.03 | -0.05 | -0.044 | -0.01 | 0 | -0.057 |
| β39 | 0.05 | 0.09 | 0.095 | -0.016 | 0.016 | -0.038 |
| β40 | 0.01 | 0.02 | 0.079 | 0.028 | 0.059 | 0.02 |
| β41 | 0 | 0.01 | -0.082 | -0.005 | -0.012 | 0 |
| β42 | 0 | 0.08 | 0.028 | 0 | 0 | 0 |
| β43 | -0.01 | -0.12 | -0.003 | -0.005 | 0 | 0.009 |
| β44 | -0.06 | -0.08 | -0.095 | -0.055 | -0.032 | -0.063 |
| β45 | 0 | -0.02 | 0.031 | 0.045 | 0.005 | 0 |
| β46 | -0.01 | -0.06 | 0.042 | 0.03 | 0.075 | 0.007 |
| β47 | 0 | 0.04 | -0.015 | 0.017 | -0.011 | 0 |
| β48 | -0.03 | -0.02 | -0.101 | -0.111 | -0.026 | 0 |
| β49 | 0.12 | 0.05 | 0.041 | 0 | 0.024 | 0 |
| β50 | 0 | -0.03 | 0.049 | 0.02 | -0.006 | 0 |
| β51 | 0.03 | -0.06 | 0.067 | 0.016 | 0.059 | 0 |
| β52 | 0.05 | 0.06 | -0.018 | 0.004 | 0.058 | 0.011 |
| β53 | 0 | -0.01 | 0.033 | 0 | 0.035 | 0 |
| β54 | -0.02 | -0.03 | -0.075 | -0.038 | -0.109 | -0.007 |
| β55 | 0 | 0.02 | -0.029 | 0 | 0.01 | 0.005 |
| β56 | 0.03 | -0.01 | 0.11 | -0.058 | 0.011 | 0 |
| β57 | 0.03 | 0.06 | -0.053 | 0 | 0 | -0.041 |
| β58 | 0.04 | 0.06 | -0.033 | 0 | -0.107 | 0.004 |
| β59 | -0.04 | 0.05 | -0.033 | 0.071 | 0.01 | 0.092 |
| β60 | -0.1 | 0.01 | -0.157 | 0.064 | -0.031 | 0.051 |
| β61 | -0.06 | -0.07 | -0.033 | 0 | 0 | 0.021 |
| β62 | 0.01 | 0.04 | 0.059 | 0.016 | 0.053 | 0 |

|  | High Beta, rest | High Beta, maintenance | Low Gamma, rest | Low Gamma, maintenance |
| --- | --- | --- | --- | --- |
| C | 100 | 0.01 | 1 | 0.1 |
| β0 (intercept) | 0 | 0 | 0 | 0.003 |
| β1 | -0.08 | 0 | -0.1 | -0.005 |
| β2 | -0.14 | -0.018 | -0.06 | -0.061 |
| β3 | -0.02 | 0 | 0.04 | 0 |
| β4 | -0.11 | -0.001 | 0 | -0.029 |
| β5 | 0.06 | 0 | 0.01 | 0 |
| β6 | -0.03 | 0 | 0 | 0.025 |
| β7 | -0.16 | 0 | -0.02 | 0.007 |
| β8 | -0.04 | 0 | 0.04 | 0.083 |
| β9 | 0.16 | 0 | 0 | 0.006 |
| β10 | 0.04 | 0 | -0.01 | -0.033 |
| β11 | 0.26 | 0 | -0.01 | -0.049 |
| β12 | 0.1 | 0 | -0.03 | -0.077 |
| β13 | 0.05 | 0 | -0.07 | 0 |
| β14 | -0.03 | 0 | -0.03 | 0.02 |
| β15 | -0.15 | 0 | 0 | -0.043 |
| β16 | 0.03 | 0 | -0.07 | -0.014 |
| β17 | -0.08 | 0 | -0.05 | -0.036 |
| β18 | 0.03 | 0 | 0.05 | 0 |
| β19 | 0 | 0 | 0.05 | 0.048 |
| β20 | 0 | 0.003 | 0 | 0 |
| β21 | 0.05 | 0 | 0.07 | 0.019 |
| β22 | 0.04 | 0 | 0 | -0.012 |
| β23 | -0.02 | 0 | -0.06 | 0 |
| β24 | 0.04 | 0 | 0.06 | 0.06 |
| β25 | -0.09 | 0 | -0.05 | 0 |
| β26 | 0.03 | 0 | 0 | 0 |
| β27 | 0 | 0 | -0.03 | -0.02 |
| β28 | -0.04 | 0 | 0.01 | 0.004 |
| β29 | 0.07 | -0.02 | 0.02 | -0.005 |
| β30 | 0.15 | 0 | 0.01 | 0 |
| β31 | -0.07 | 0 | -0.05 | -0.037 |
| β32 | -0.16 | -0.016 | -0.09 | -0.037 |
| β33 | -0.03 | 0 | 0.03 | -0.002 |
| β34 | 0.19 | 0 | 0.02 | 0 |
| β35 | 0.21 | 0 | 0.11 | 0 |
| β36 | -0.21 | 0 | -0.1 | -0.007 |
| β37 | 0.01 | 0 | 0.01 | 0 |
| β38 | 0.04 | 0 | 0.08 | 0.029 |
| β39 | 0.16 | 0 | 0.08 | 0 |
| β40 | 0.04 | 0 | -0.02 | -0.045 |
| β41 | -0.31 | 0 | 0.01 | 0 |
| β42 | -0.05 | 0 | -0.04 | -0.06 |
| β43 | 0.03 | 0 | -0.07 | -0.032 |
| β44 | -0.08 | 0 | 0.12 | 0.044 |
| β45 | 0.06 | 0 | 0.05 | 0.015 |
| β46 | 0.13 | 0 | 0.02 | 0 |
| β47 | -0.08 | 0 | -0.08 | 0.015 |
| β48 | -0.1 | 0 | 0.04 | 0.115 |
| β49 | -0.04 | 0 | 0.04 | 0.005 |
| β50 | 0.1 | 0 | 0.05 | 0 |
| β51 | -0.06 | 0 | 0.02 | 0 |
| β52 | 0.06 | 0 | 0.06 | -0.031 |
| β53 | -0.04 | 0 | 0 | 0.009 |
| β54 | -0.03 | 0 | -0.03 | 0.059 |
| β55 | 0.07 | 0 | 0 | 0 |
| β56 | 0.04 | 0 | -0.06 | -0.04 |
| β57 | -0.1 | 0 | -0.08 | -0.05 |
| β58 | -0.07 | 0 | 0 | 0 |
| β59 | 0.01 | 0 | -0.05 | 0.042 |
| β60 | -0.13 | 0 | -0.05 | 0.025 |
| β61 | 0 | 0 | 0.05 | 0.051 |
| β62 | 0.08 | 0 | 0.02 | 0.022 |

|  | High Gamma, rest | High Gamma, maintenance | Aperiodic slope, rest | Aperiodic slope, maintenance |
| --- | --- | --- | --- | --- |
| C | 1 | 0.1 | 0.01 | 0.01 |
| β0 (intercept) | -0.004 | 0.008 | 0 | 0 |
| β1 | 0.058 | -0.003 | 0 | 0 |
| β2 | 0.067 | 0.036 | 0 | 0 |
| β3 | 0.007 | 0 | 0 | 0 |
| β4 | 0.051 | 0 | 0 | 0 |
| β5 | 0.012 | 0 | 0 | 0 |
| β6 | -0.023 | 0 | 0 | 0 |
| β7 | 0.017 | 0 | 0.007 | 0 |
| β8 | 0.011 | 0.081 | 0 | 0 |
| β9 | -0.037 | 0.006 | 0 | 0 |
| β10 | 0.009 | 0 | 0 | 0 |
| β11 | -0.123 | -0.112 | 0 | 0 |
| β12 | 0.072 | -0.114 | 0 | -0.001 |
| β13 | 0.007 | 0 | 0.002 | 0 |
| β14 | 0.006 | 0.024 | 0 | 0 |
| β15 | 0.02 | 0 | 0 | 0 |
| β16 | 0.01 | 0.005 | 0 | 0 |
| β17 | -0.002 | 0.005 | 0 | 0 |
| β18 | -0.031 | 0.015 | 0 | 0 |
| β19 | -0.004 | 0 | 0 | 0 |
| β20 | 0.026 | -0.04 | 0 | 0 |
| β21 | 0.004 | 0 | 0 | 0 |
| β22 | 0.031 | 0.03 | 0 | 0 |
| β23 | 0 | 0 | 0 | 0 |
| β24 | 0.015 | 0.021 | 0 | 0 |
| β25 | -0.028 | -0.012 | 0 | -0.016 |
| β26 | -0.012 | 0.01 | -0.003 | 0 |
| β27 | 0.073 | 0.035 | -0.001 | -0.012 |
| β28 | -0.015 | 0 | 0 | 0 |
| β29 | -0.023 | 0.004 | -0.013 | -0.014 |
| β30 | -0.016 | -0.027 | 0 | 0 |
| β31 | 0.028 | -0.029 | 0 | -0.013 |
| β32 | 0.017 | 0.005 | 0 | 0 |
| β33 | -0.022 | -0.016 | 0 | 0 |
| β34 | 0 | -0.034 | 0 | 0.013 |
| β35 | -0.002 | -0.02 | -0.033 | 0 |
| β36 | -0.024 | -0.016 | 0 | 0 |
| β37 | -0.019 | 0 | 0 | 0 |
| β38 | 0.018 | -0.011 | 0 | 0 |
| β39 | 0.02 | 0.018 | 0 | 0 |
| β40 | -0.001 | 0 | 0 | 0 |
| β41 | 0.051 | 0.013 | 0 | 0 |
| β42 | -0.051 | -0.071 | 0 | 0 |
| β43 | -0.055 | 0 | 0 | 0 |
| β44 | 0.012 | 0.079 | 0 | 0 |
| β45 | 0.009 | 0.026 | 0 | -0.002 |
| β46 | 0.058 | 0.039 | 0 | 0 |
| β47 | 0.032 | 0.01 | 0 | 0 |
| β48 | -0.055 | 0.044 | 0 | 0 |
| β49 | 0.05 | 0.014 | 0 | 0 |
| β50 | -0.041 | -0.036 | 0 | 0 |
| β51 | 0.05 | 0.008 | 0 | 0 |
| β52 | -0.014 | 0 | 0 | 0 |
| β53 | -0.075 | 0.019 | 0 | 0 |
| β54 | -0.058 | 0.043 | 0 | 0 |
| β55 | -0.06 | -0.041 | 0 | 0 |
| β56 | -0.093 | -0.008 | 0 | 0 |
| β57 | 0.014 | 0.045 | -0.071 | -0.024 |
| β58 | 0.002 | -0.027 | -0.074 | -0.007 |
| β59 | -0.002 | -0.023 | -0.026 | -0.01 |
| β60 | 0.008 | -0.001 | 0 | 0 |
| β61 | 0.122 | 0.13 | 0 | 0 |
| β62 | 0.029 | 0.008 | 0 | -0.036 |

# Uncorrected band power results


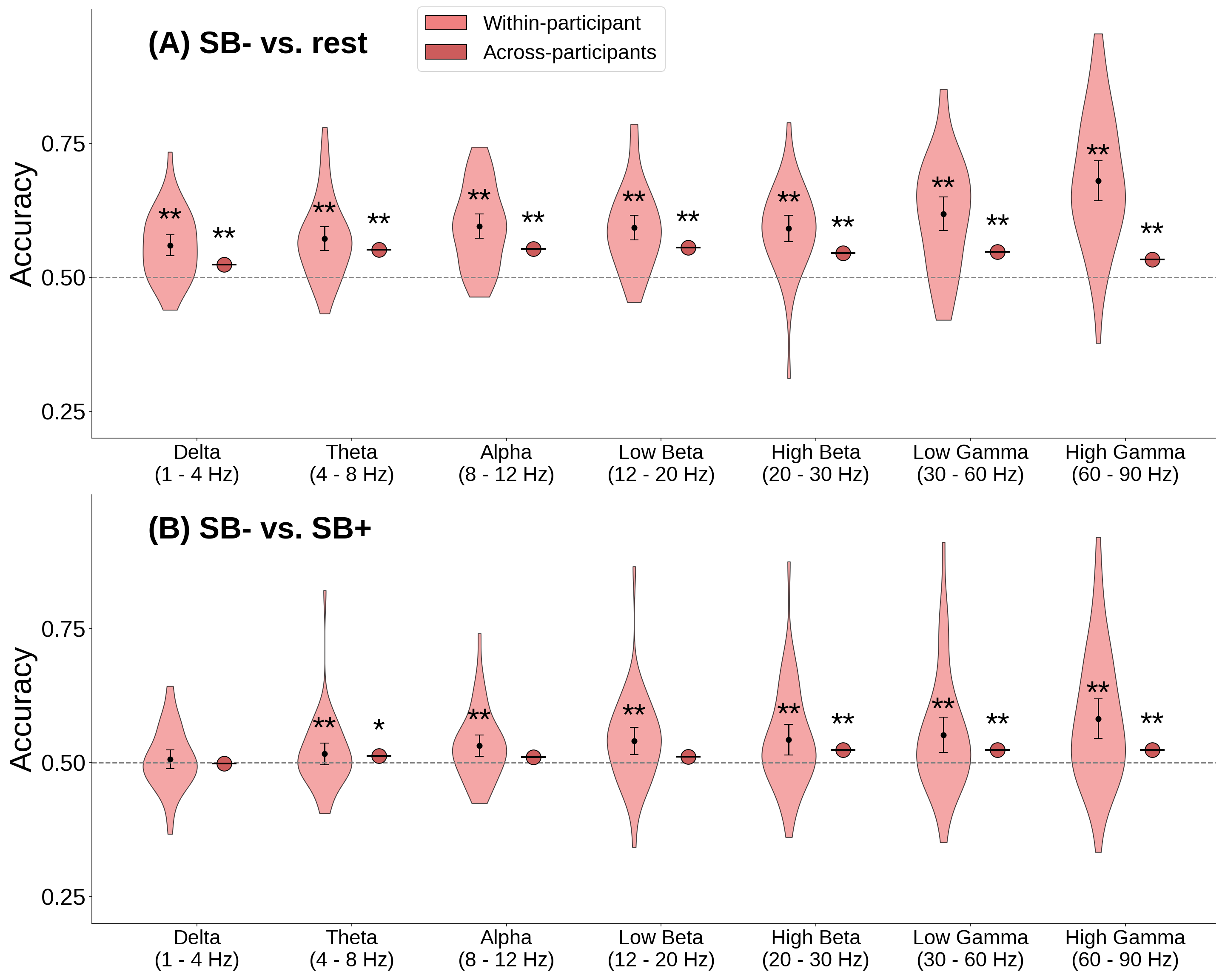
Figure 2: Classification of self-boundaries on the bases of band power in different frequency bands (not corrected by, i.e., including, the aperiodic component) by means of logistic regression. Within-participant accuracy signifies mean classification accuracy across within-participant classification attempts. Error bars indicate 95% confidence intervals. Dashed line indicates chance level (50%).

# Correlation with lifetime meditation hours and phenomenology

# 27 Hz


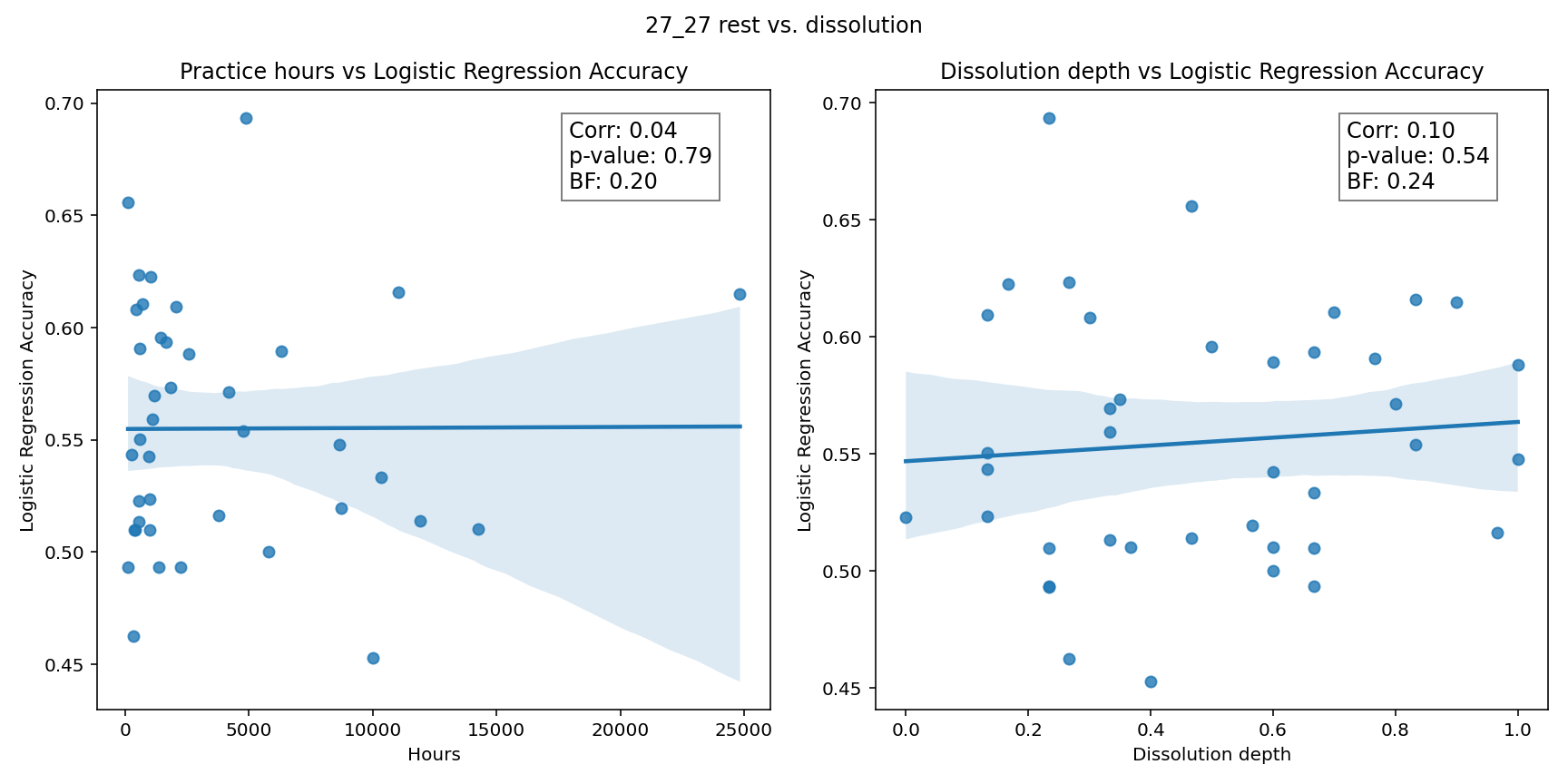


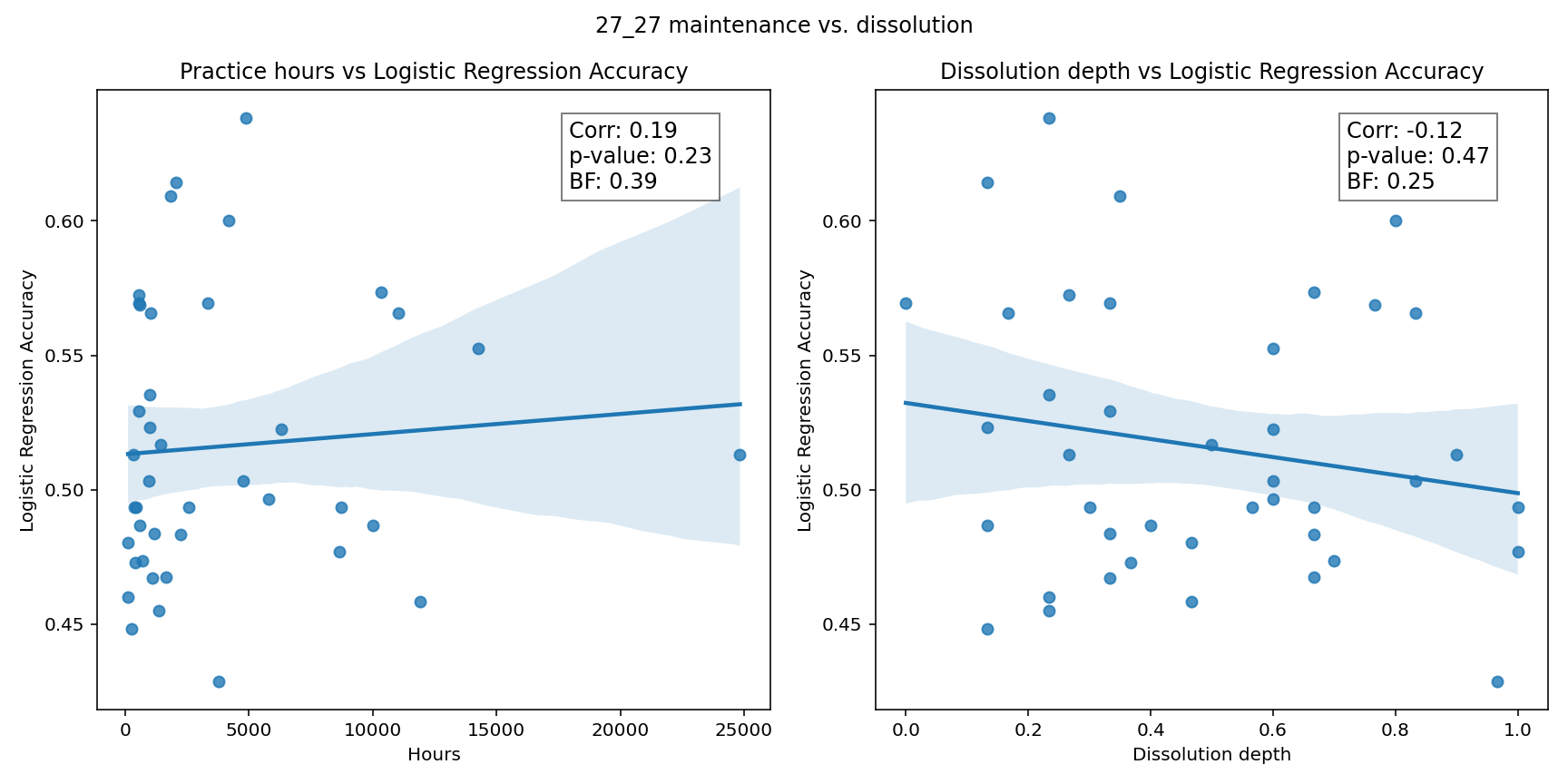
Figure 3: Spearman correlations between Logistic Regression accuracy and practice hours (left) and phenomenological dissolution depth (right). Top: SB- vs. rest; bottom: SB- vs. SB+. BF abbreviates Bayes factor.


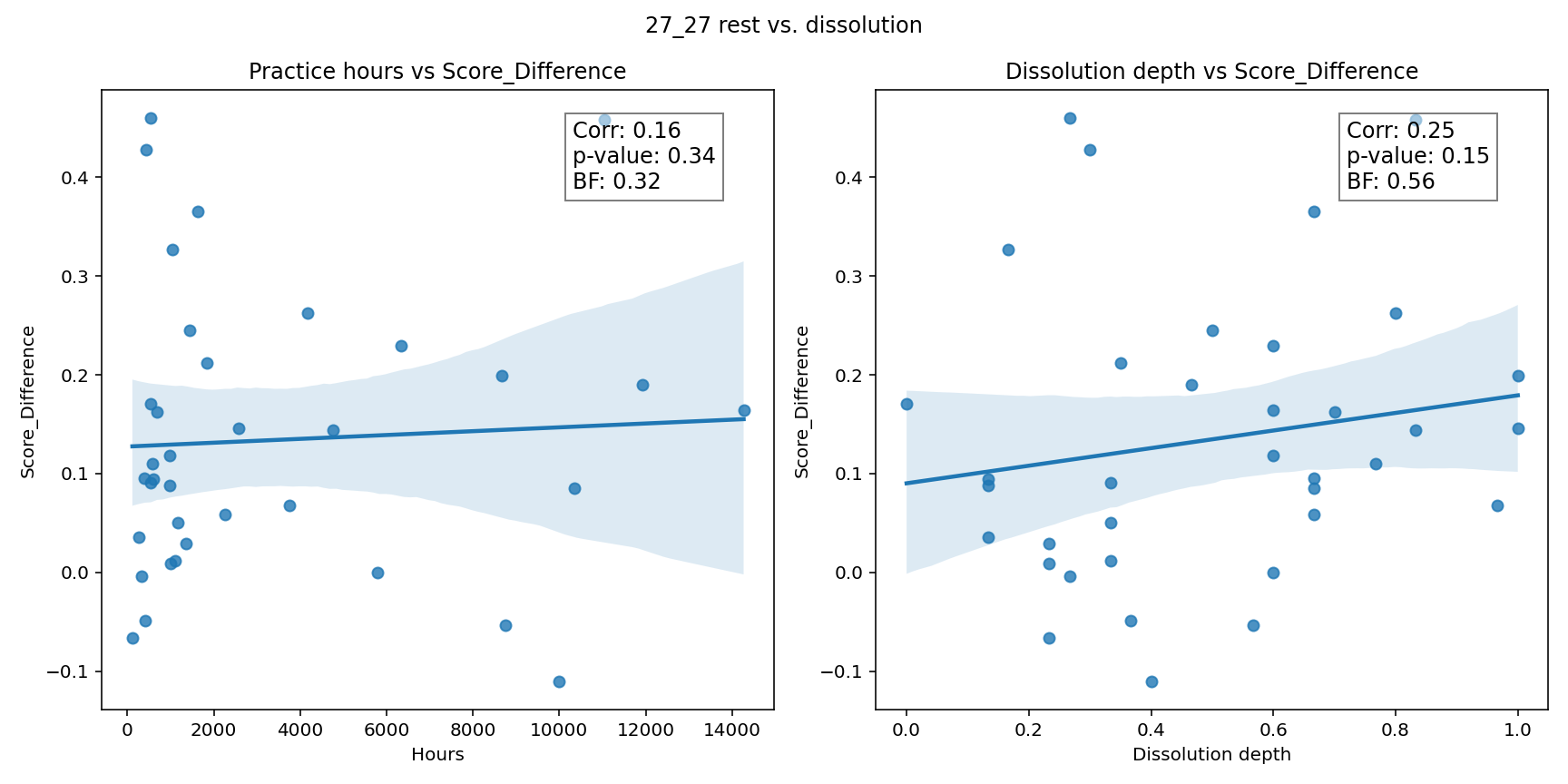


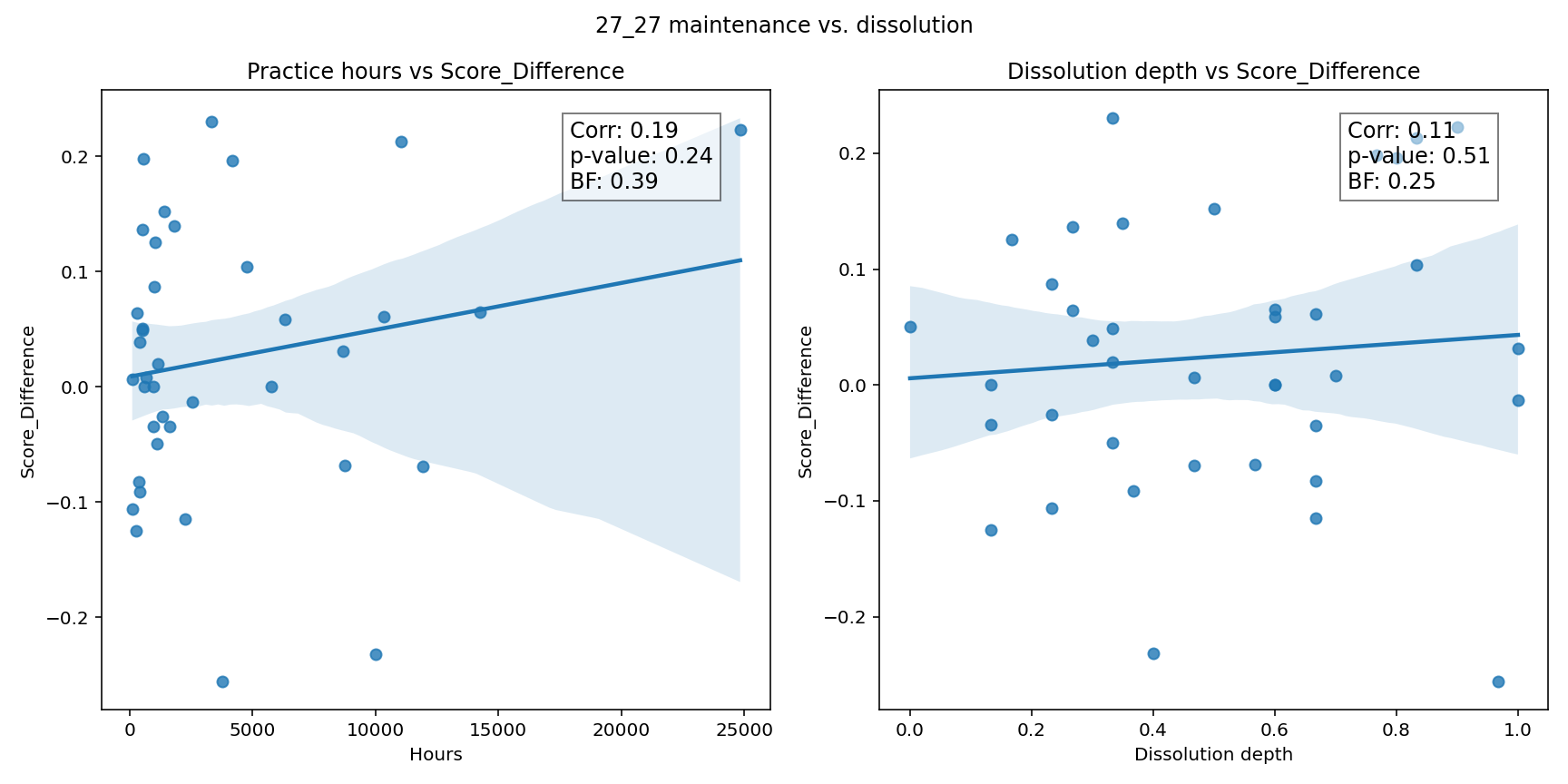
Figure 4: Spearman correlations between average logit difference between classes and practice hours (left) and phenomenological dissolution depth (right). Top: SB- vs. rest; bottom: SB- vs. SB+. BF abbreviates Bayes factor.

# Lempel-Ziv complexity


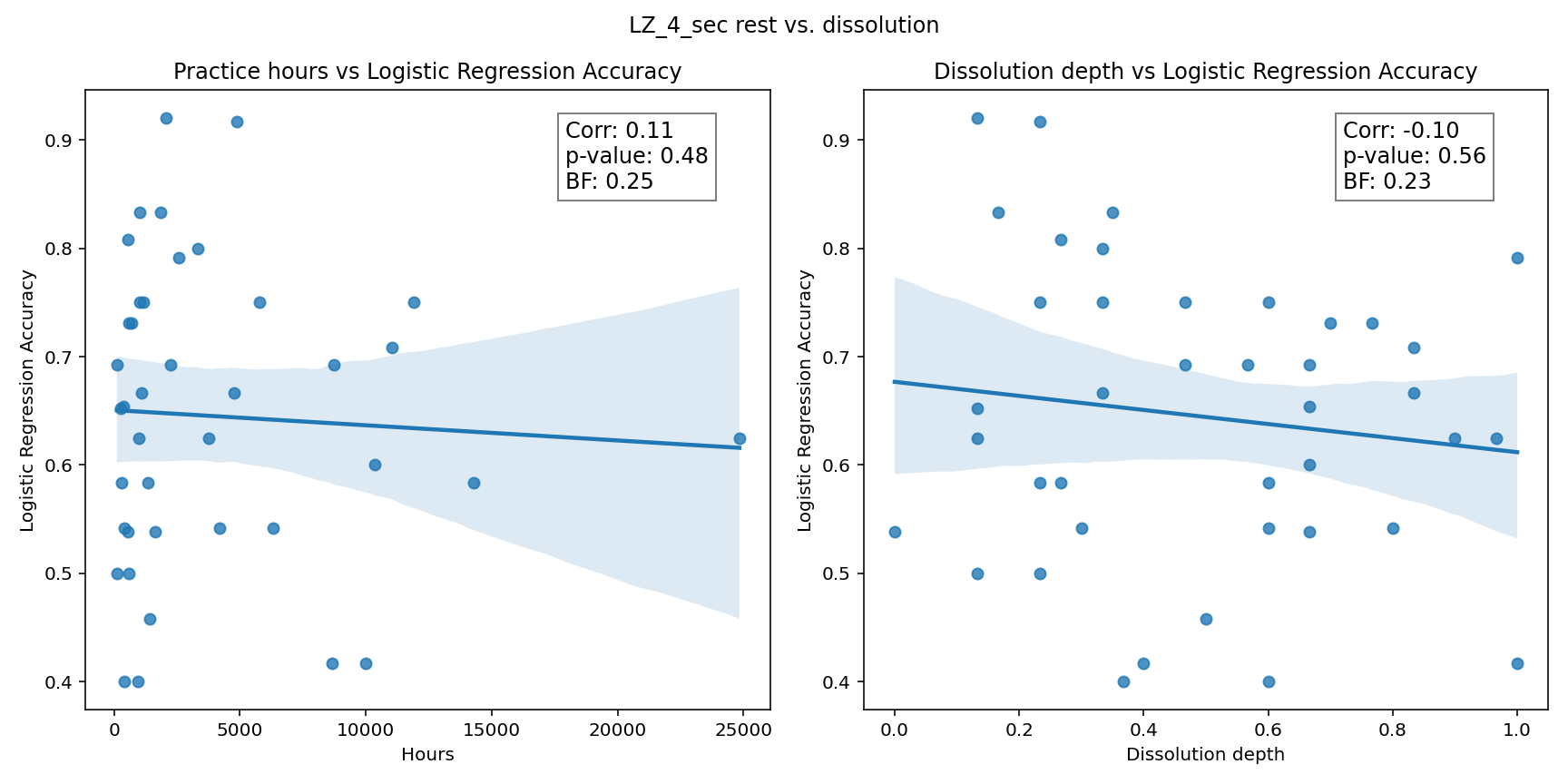


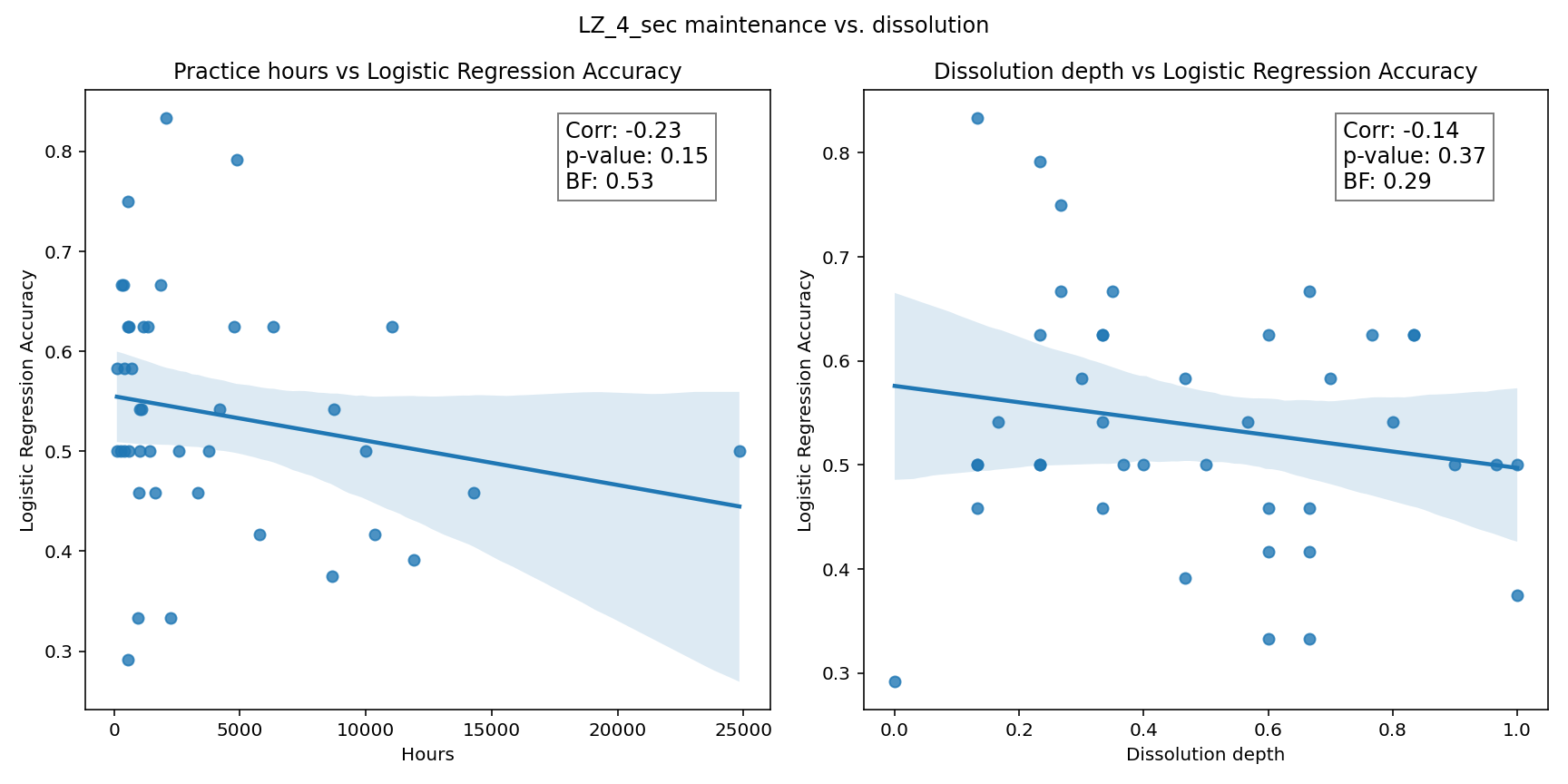
Figure 5: Spearman correlations between Logistic Regression accuracy and practice hours (left) and phenomenological dissolution depth (right). Top: SB- vs. rest; bottom: SB- vs. SB+. BF abbreviates Bayes factor.


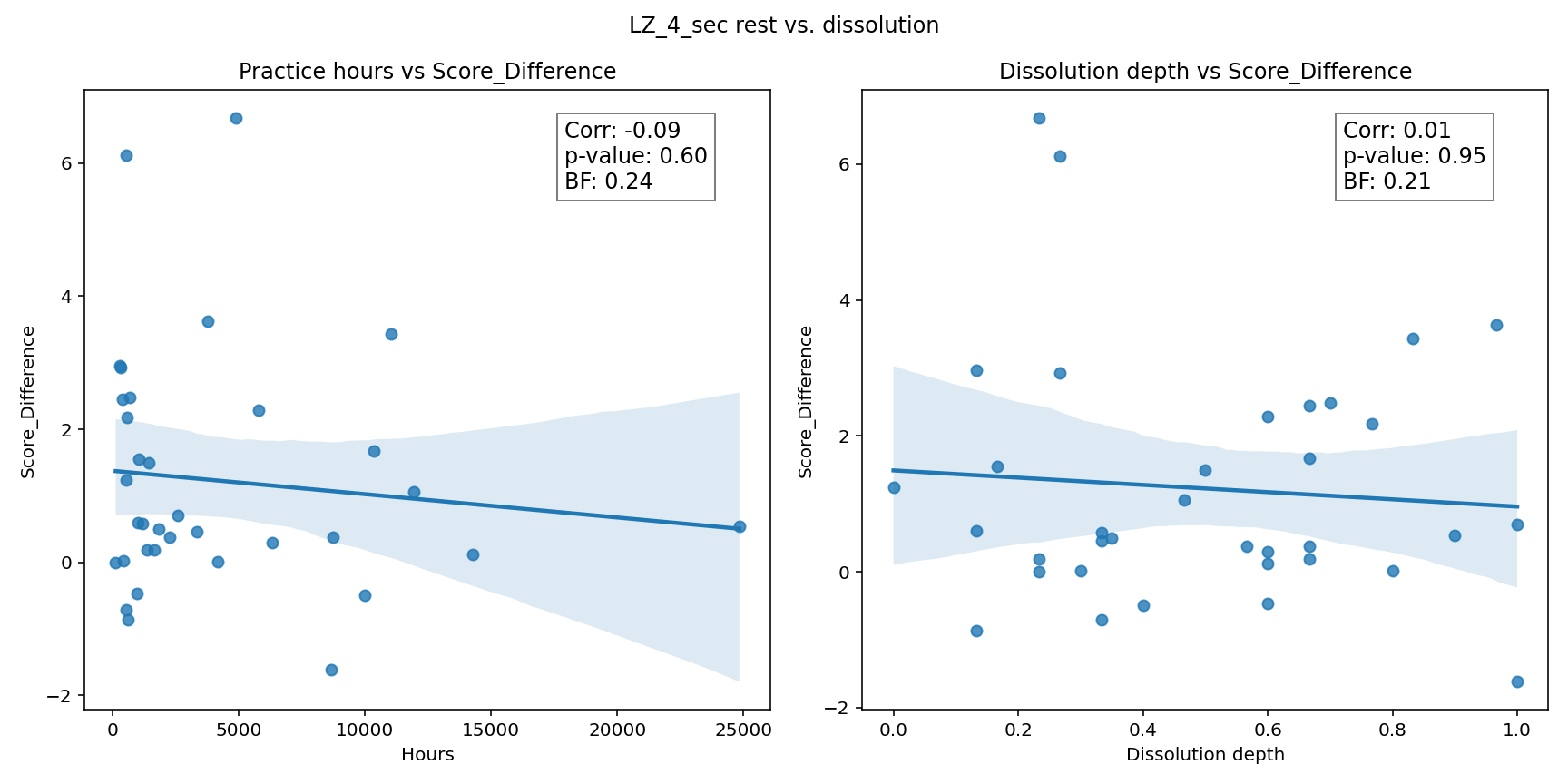


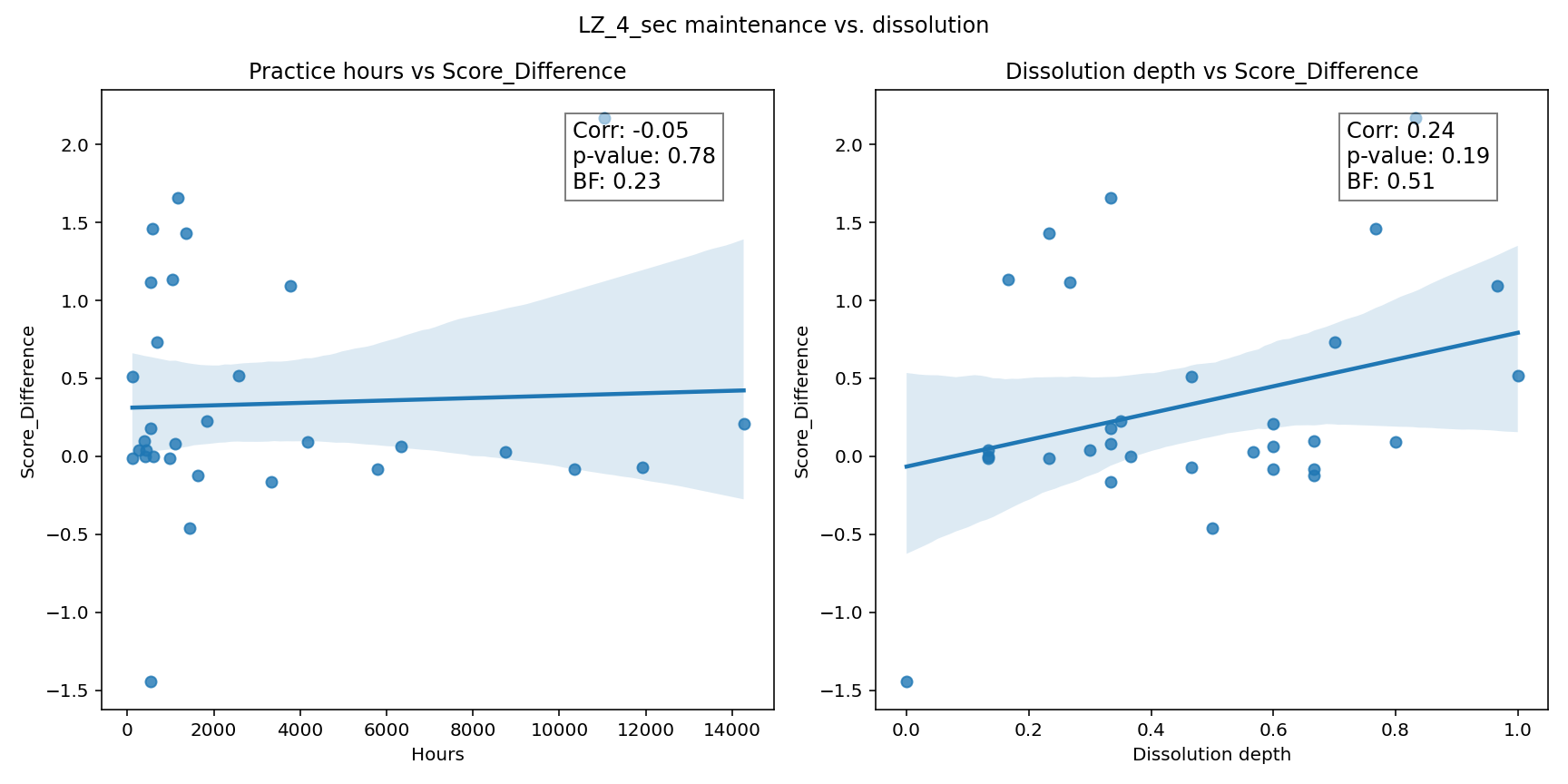
Figure 6: Spearman correlations between average logit difference between classes and practice hours (left) and phenomenological dissolution depth (right). Top: SB- vs. rest; bottom: SB- vs. SB+. BF abbreviates Bayes factor.

# Correlations between brain regions

To quantify inter-regional dependencies, pairwise Pearson correlations were computed between regional epoched data for each subject, yielding individual correlation matrices. Correlation coefficients were Fisher z-transformed, averaged across subjects, and inverse-transformed to obtain group-level mean correlation matrices.


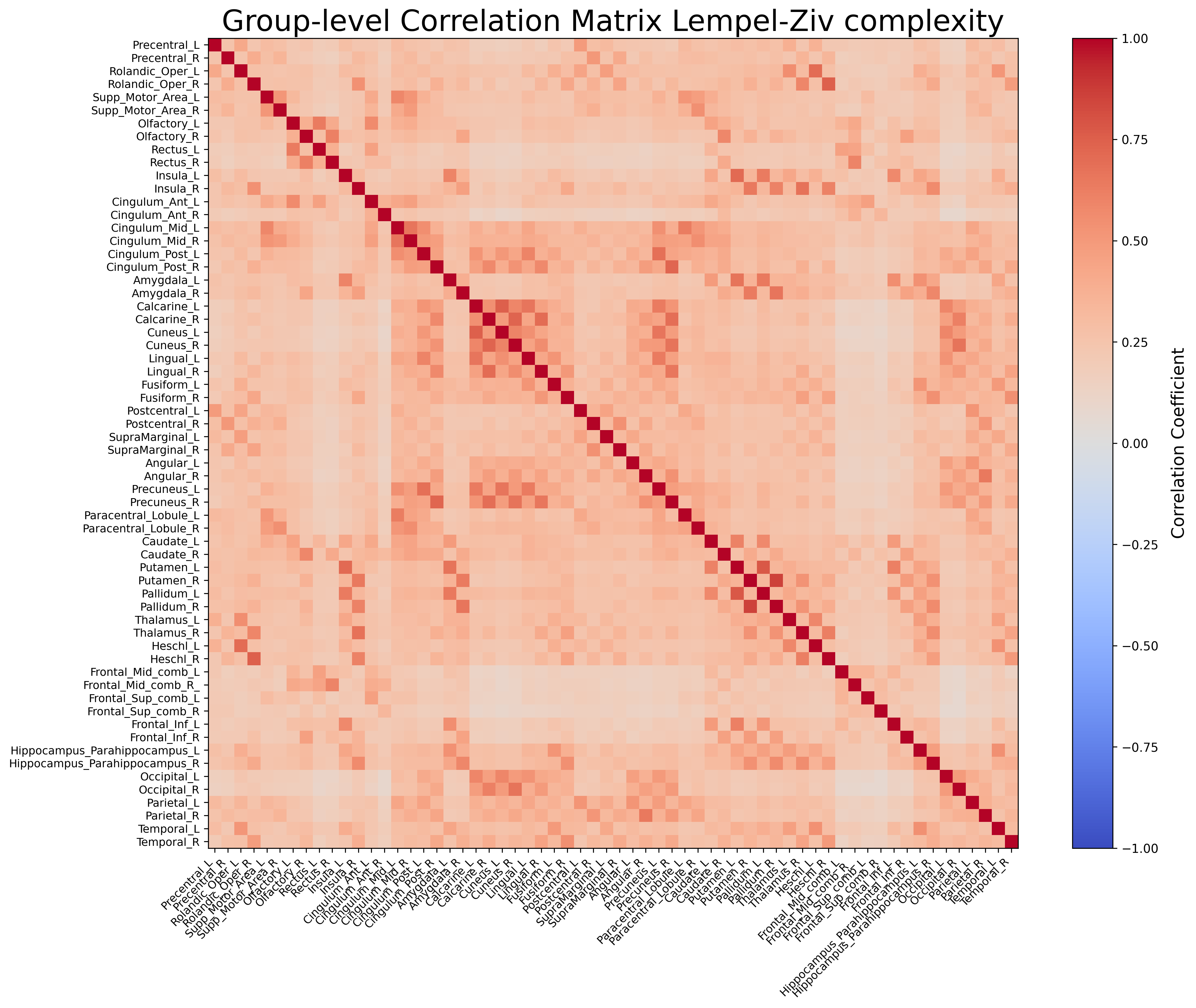


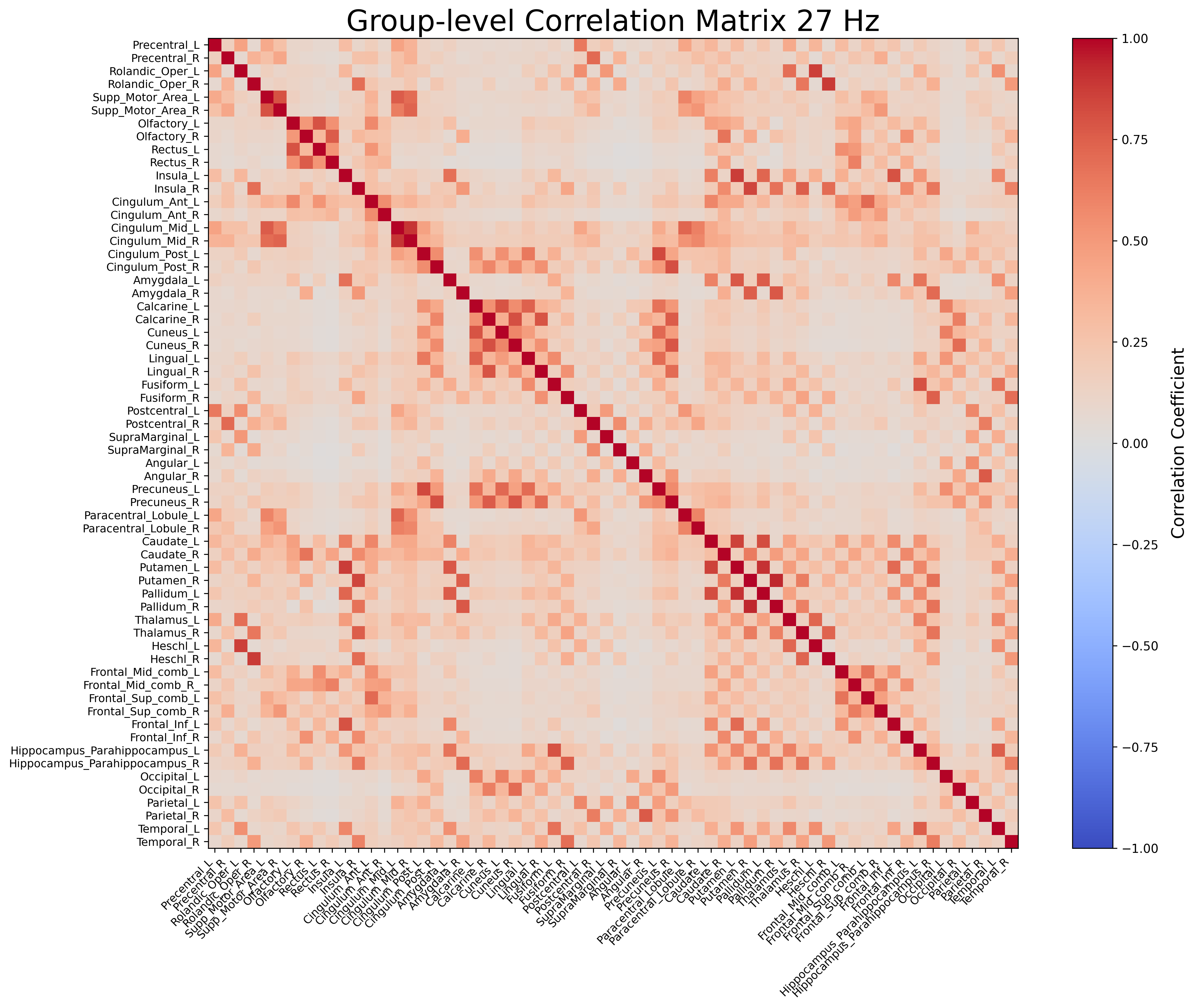


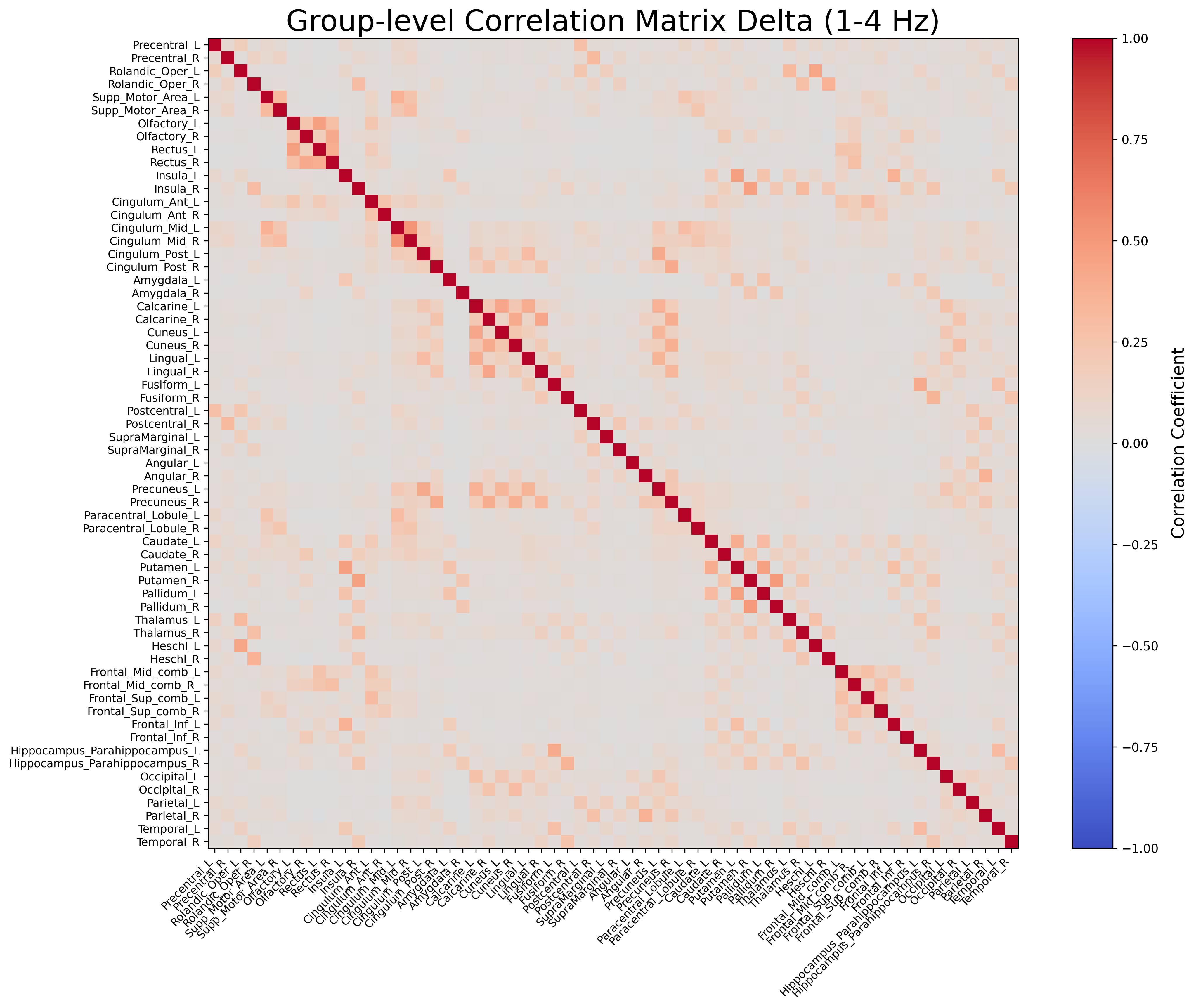


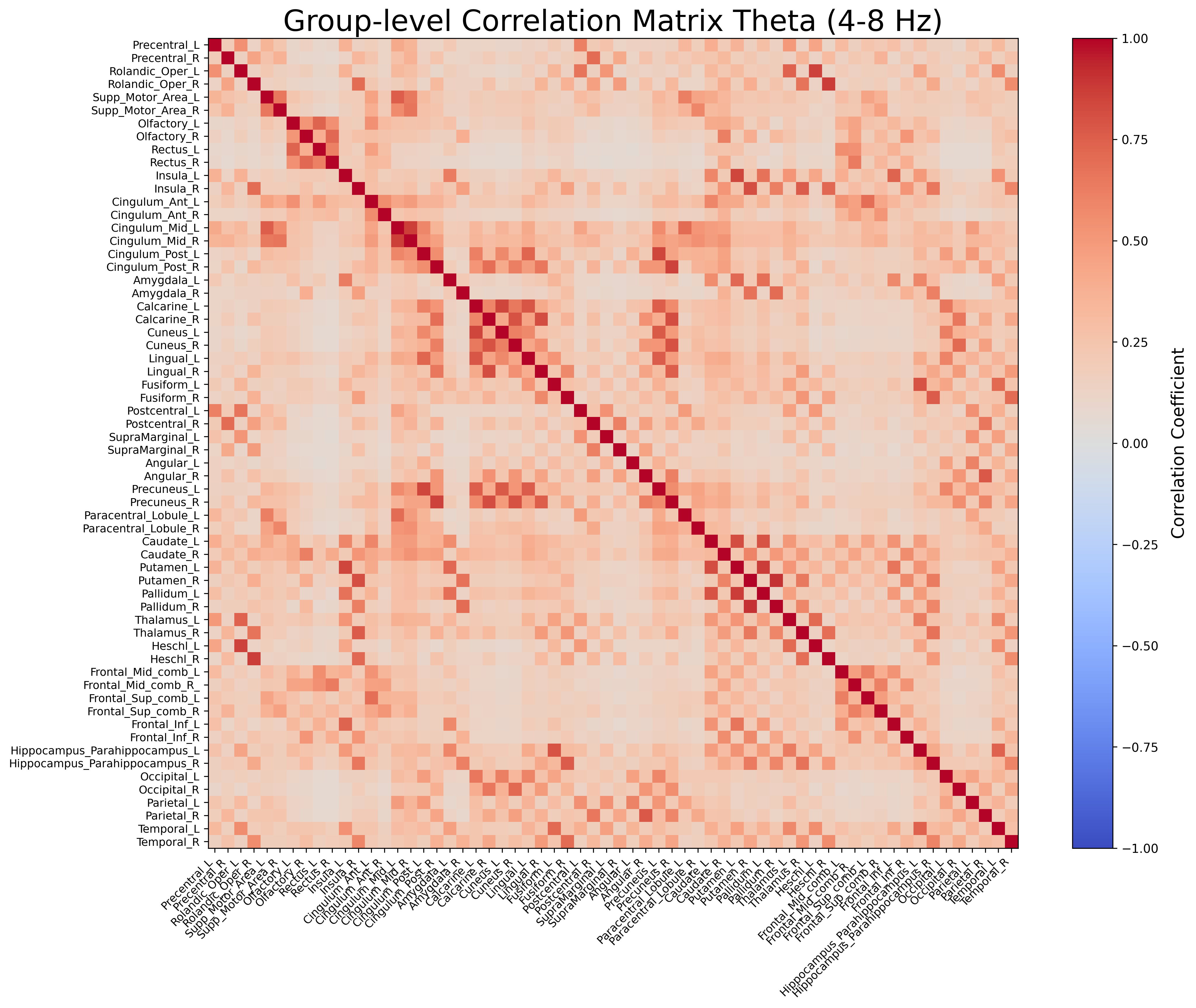


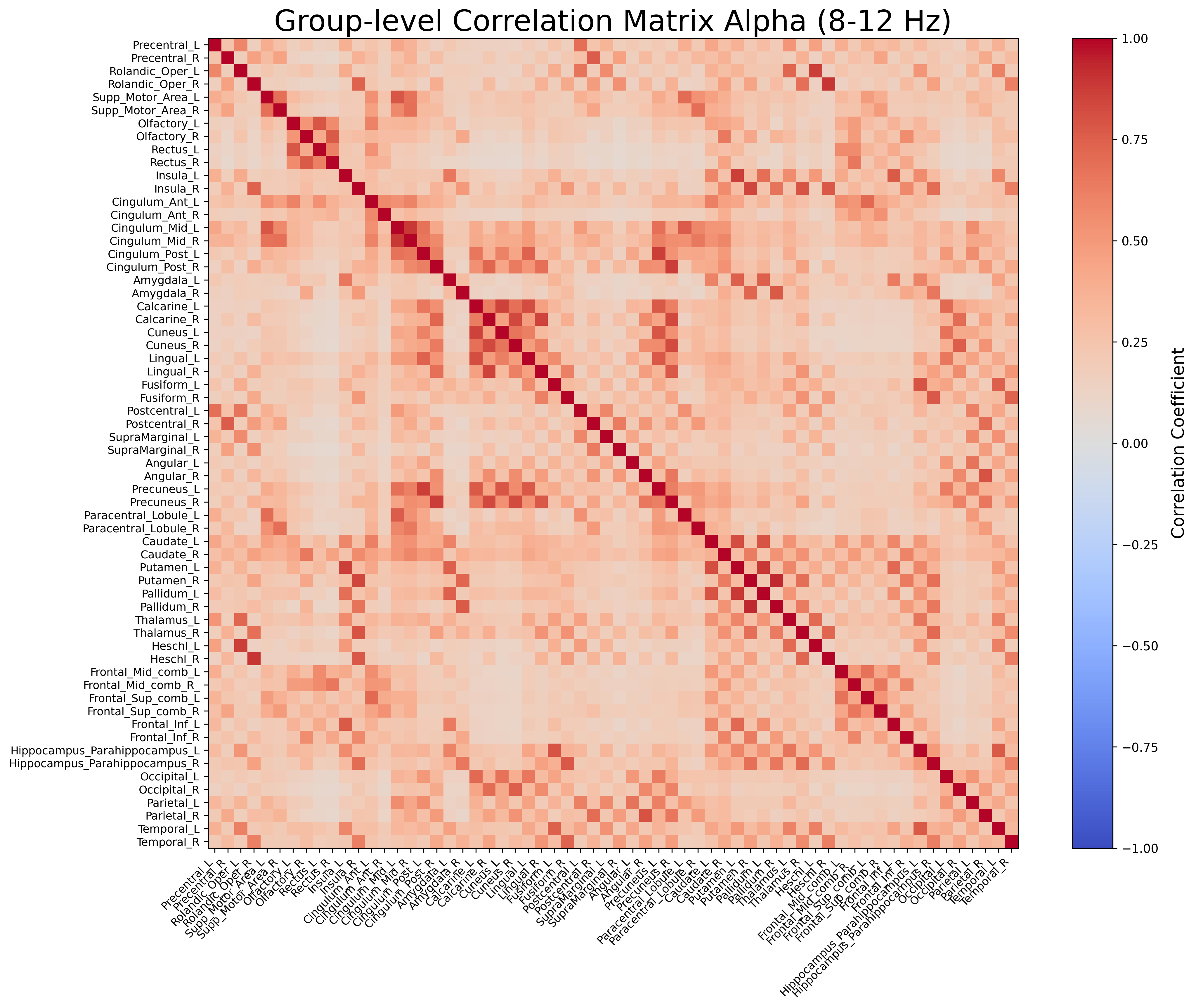


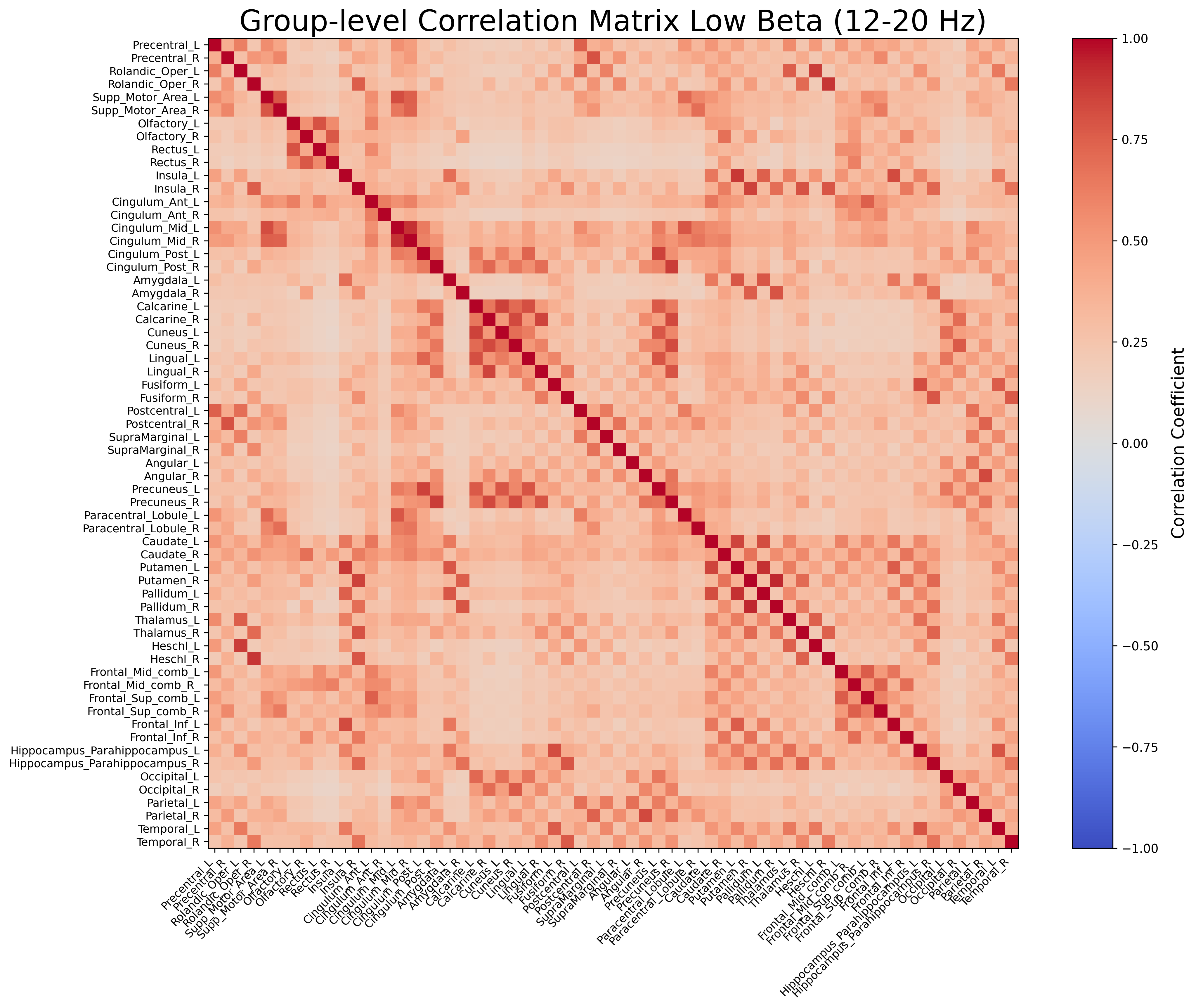


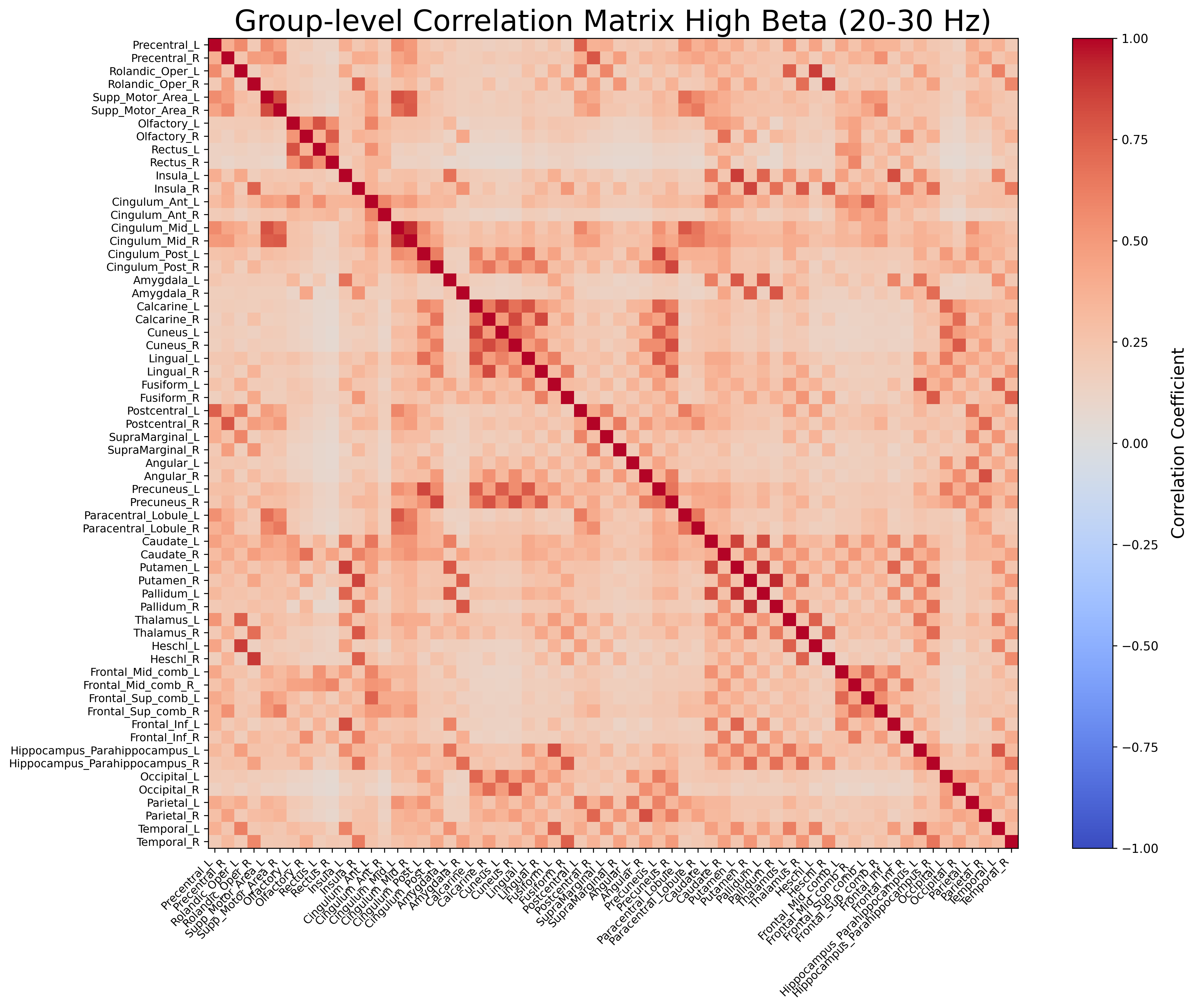


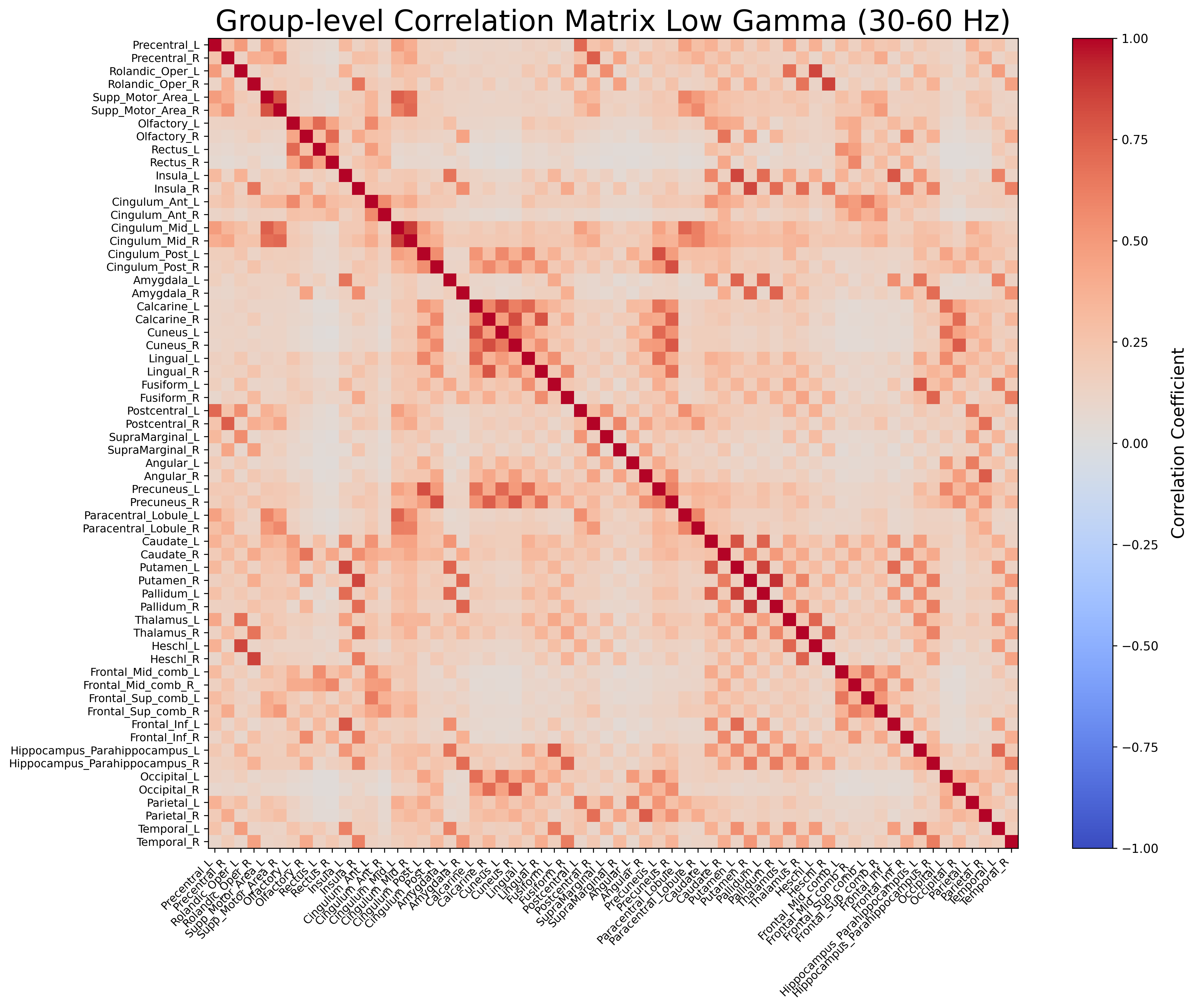


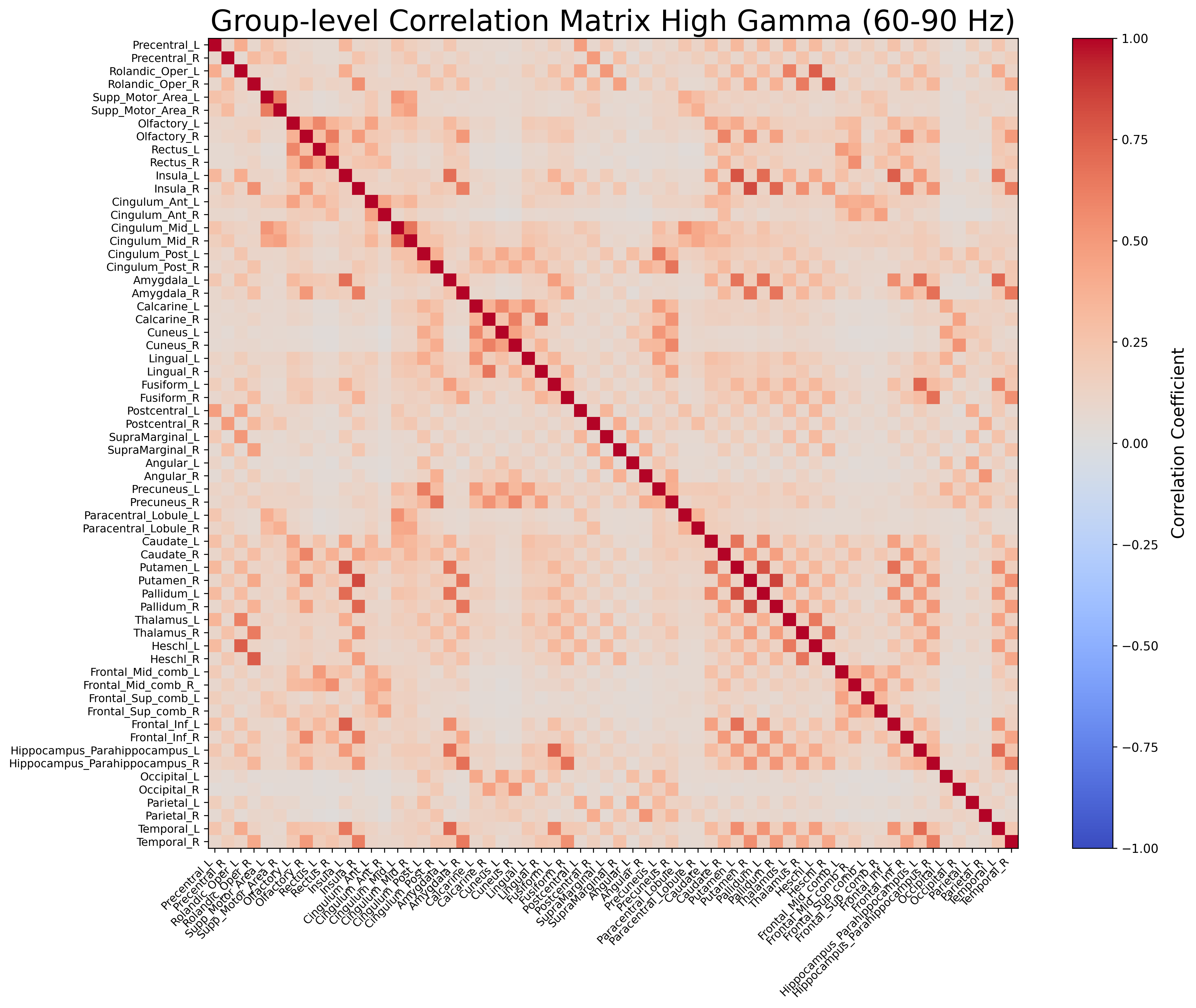


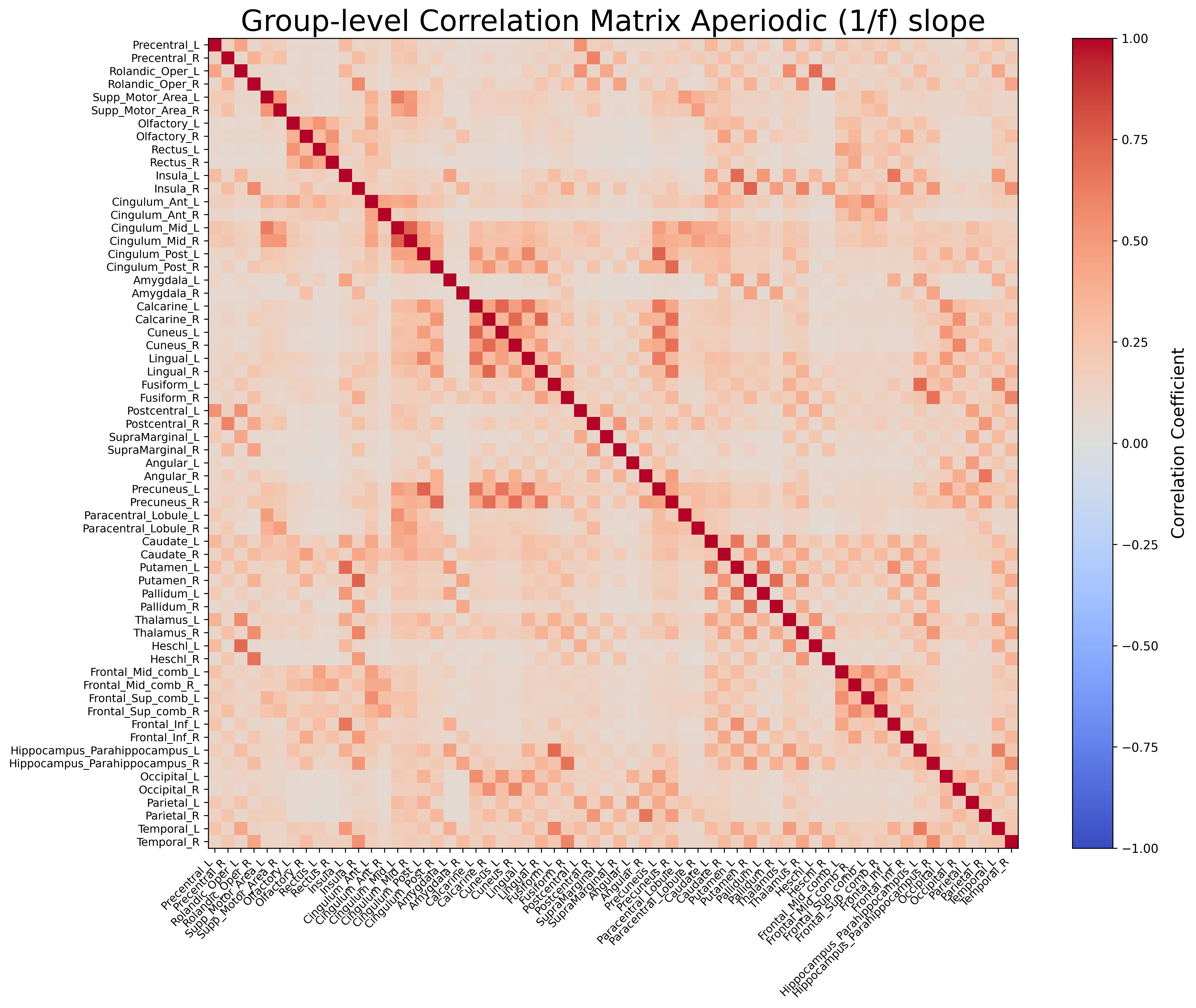

Supplement: Supplementary file 1 — Data S1: hbm70440‐sup‐0001‐Supplementary_materials.docx. [file HBM-47-e70440-s001.docx]
